# Photo-Cross-Linking Polymersome Nanoreactors with Size-Selective Permeability

**DOI:** 10.1021/acs.macromol.2c00248

**Published:** 2022-06-30

**Authors:** Sjoerd J. Rijpkema, Rik van Egeraat, Wei Li, Daniela A. Wilson

**Affiliations:** Institute for Molecules and Materials, Radboud University, Heyendaalseweg 135, Nijmegen, 6525 AJ, The Netherlands

## Abstract

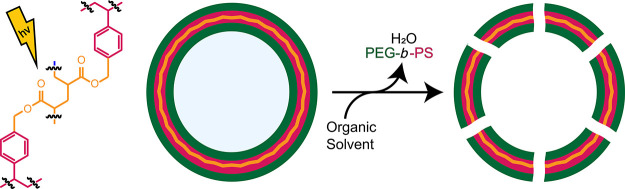

The design of stable, inert, and permeable nanoreactors remains a challenge due to the additives required to create a cross-linked network, limiting their potential for catalysis. Polymersomes are nanovesicles self-assembled from amphiphilic block copolymers that can act as nanoreactors by encapsulating catalysts. A major restriction toward their use is their stability and reduced permeability. In order to overcome this, polymersome membranes can be cross-linked to retain their shape and function. Here, we report the synthesis of a PEG-*b*-P(S-*co*-4-VBA) polymer, which can self-assemble into polymersomes and subsequently be cross-linked using UV light. We demonstrate that these polymersomes are stable over a long period of time in various organic solvents, that incorporation of functional handles on their surface is possible, and that they are able to undergo reactions. Additionally, we show that co-assembly with up to 40% PEG-*b*-PS present results in the formation of pores in the membrane structure, which allows for the structure to be used as a nanoreactor. By encapsulating a platinum nanocatalyst, we are able to catalyze the depropargylation of a small coumarin substrate, which was able to enter and leave the porous nanoreactor.

## Introduction

In nature, chemical conversions commonly take place in a confined environment to protect the catalyst and deter any unwanted side reaction. Inspired by this, scientists have developed a plethora of polymeric nanoreactors, vesicles that host a catalyst, which are thus able to protect it from a number of environmental influences.^1−5^ This can lead to both improved performance^6−9^ and more facile recovery^10−12^ of the catalyst. Various types of nanoreactors have been made, and polymersomes^13^ have shown to be promising candidates due to the versatility of their shape, size, and properties.^14,15^ The first polymersome used as a nanoreactor was published by Meier *et al*.^16^ Nowadays, a plethora of different polymers that form polymersomes are known.^15,17−19^ Specifically, amphipathic block copolymers like poly(ethylene glycol)-*b*-polystyrene (PEG-*b*-PS) are known to form spherical bilayered vesicles under osmotic stress, creating water-stable polymersome structures that can shape-transform.^17,20^ Despite being physically stable structures,^21^ due to their supramolecular nature, they are prone to disassemble under many conditions. This limits the use of polymersomes as nanoreactors and nanomotors to mild reaction conditions and polar solvents.^20,22^ To combat this issue, chemical cross-linking of the polymersome membrane can reinforce the structure and broaden the scope of its application.^23^

Various examples of cross-linked polymersomes are known in the literature, but many have significant drawbacks, which limits their function. For example, cross-linked vesicles have been made that also required monomers for pH-dependent loading and release of cargo, thus making it impossible to carry pH-responsive cargo.^14,24^ The use of noncovalent bonds to stabilize polymersomes is also well known.^25−27^ The downside to this type of cross-linking is that the structures are inherently less stable compared to covalent bonds and still prone to disassembly under harsh conditions. For covalent cross-linking, additives, usually metals, can be added in order to form the covalent bonds.^28−30^ Though robust, this method of cross-linking is rather complex and has significant drawbacks toward further application of the polymersome. This method requires the stoichiometric addition of small molecules or metals to cross-link the vesicle, of which traces may remain in the polymersome. These may interfere in the reaction mixture if polymersomes are used as a nanoreactor or with a loaded drug for drug delivery applications, which is detrimental to the desired function.^31^ These cross-linking reactions take multiple hours, not making them fast methods. For these reasons, a covalently cross-linked polymersome without the need for lingering reactive additives is highly desired to form a robust and functional structure. Examples are known in the literature, which circumvent additives by allowing for photo-cross-linking of the nanostructure, usually within minutes.^24,32,33^

This robustness however reduces the permeability of the polymersome for substrate molecules. Permeable membranes for polymersomes have mainly been reported using noncovalently bound polymersomes, where swelling of the membrane in aqueous media generates the permeability required for substrates to move through the membrane.^4,34,35^ When pores have to be created however, more complex solutions are used. This is mostly the incorporation of porogens, or pore generators, within the structure to afford membrane permeability. These porogens include bio- or DNA pores,^36−38^ synthetic materials responsive to light,^39−42^ or polymers that become soluble through chemical additives^29,43−45^ and have to be specifically embedded in the polymeric structure. (Semi)permeability has been added to polymersomes by allowing Irgacure 2959 fragments to recombine with radicals on the polymers, making the membrane less hydrophobic and thus allowing for the certain organic compounds to diffuse through.^33,46,47^ However, this method does not introduce pores in the membrane. A simple and versatile way to introduce and control permeability of cross-linked polymersomes using only conventional block copolymers without the requirement of additives has, to our knowledge, not been shown yet.

**Figure 1:**
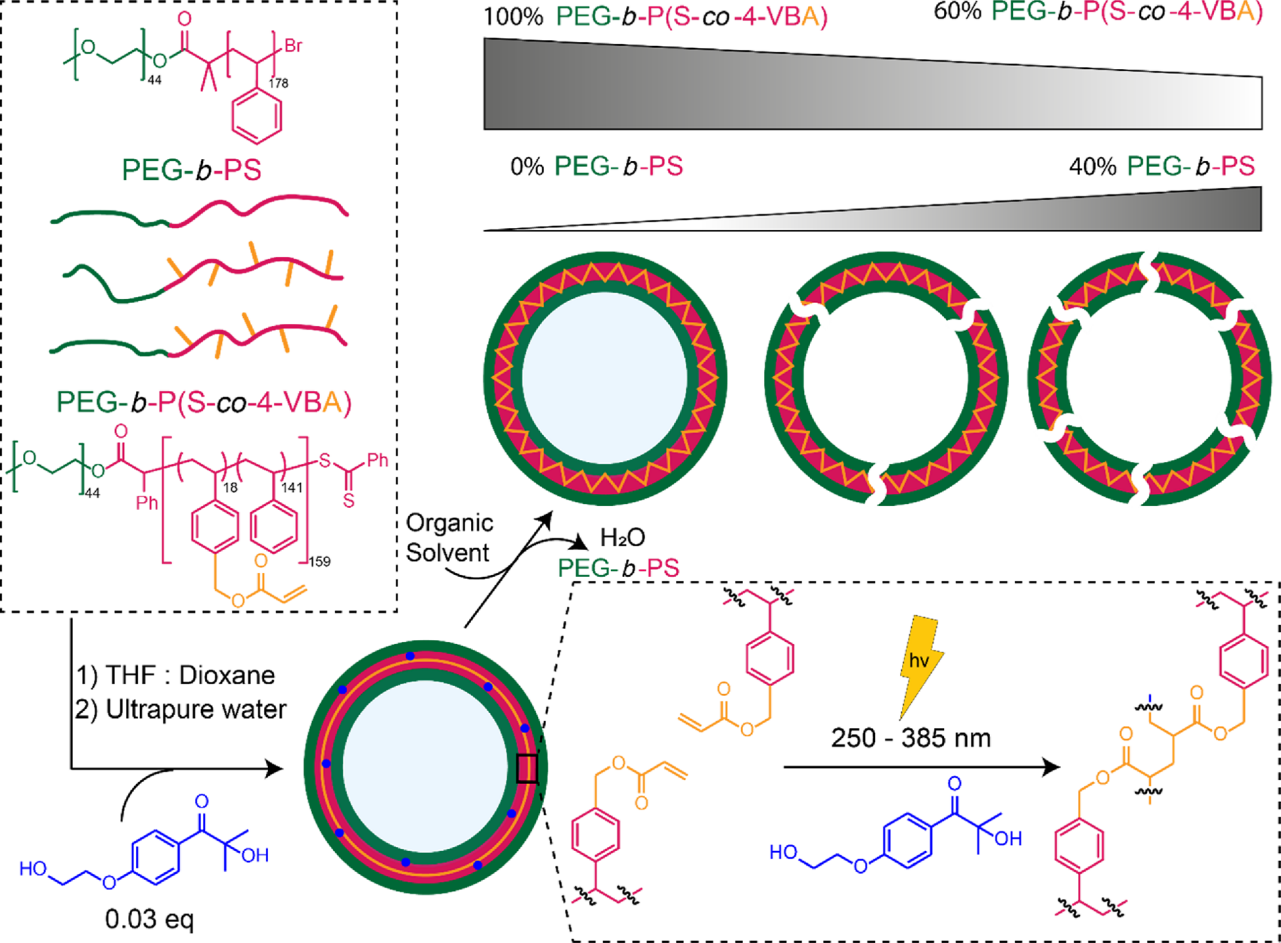
Schematic of the self-assembly of cross-linked polymersomes. First, polymersomes are made *via* self-assembly upon slow addition of water (0.5 mL, 1 mL h^–1^) to PEG-*b*-P(S-*co*-4-VBA) or a PEG-*b*-P(S-*co*-4-VBA) and PEG-*b*-PS mixture in THF/1,4-dioxane (4:1 v/v) with a minimal amount of Irgacure 2959. Then, using UV light, the polymersomes are cross-linked, after which they are centrifuged and washed with organic solvent to remove water and PEG-*b*-PS to yield cross-linked polymersomes with varying pore sizes.

Based on this, we have designed a novel cross-linking methodology to stabilize polymersome vesicles and gain control over the permeability of the membrane by generation of pores (Figure 1). For this, we have designed a cross-linkable polymer close in structure to the PEG-*b*-PS polymers utilized by our group,^20^ with an acrylate moiety present in the hydrophobic part. These are often used as cross-linkers for hydrogels and resins.^48,49^ By introducing multiple acrylate groups in the hydrophobic block, a cross-linked polymer network can be formed. We describe the successful cross-linking of poly(ethylene glycol)-*b*-poly(styrene-*co*-4-vinylbenzyl acrylate) (PEG-*b*-P(S-*co*-4-VBA)) polymersomes by using UV light and only a minimal addition of Irgacure 2959, a biomedically compatible photoinitiator.^50,51^ We demonstrate that the cross-linking is highly efficient and readily adaptable to include 10% w/w functionalized non-cross-linkable polymers, which stay attached to the membrane. Moreover, we show that incorporation of a higher amount of the simple and commonly used non-cross-linkable PEG-*b*-PS polymers leads to the formation of holes in the membrane simply by washing with organic solvent. Varying the ratio between these polymers also give rise to different pore sizes, making this process tunable. Finally, we present a proof-of-concept example in which cross-linked polymersomes with holes containing platinum nanoparticles are used for the catalytic depropargylation of a coumarin, demonstrating the use of this system as a nanoreactor.

## Experimental Section

### Materials

All PEG polymers with different functional end groups were obtained from AV Chemistry. All other reagents were obtained from commercial sources and were used without purification unless otherwise stated. Solvents were dried by passing over activated alumina columns in an MBraun MB SPS800 under a nitrogen atmosphere and stored under argon. Reactions were carried without the need for an inert atmosphere unless stated otherwise, in which case the reaction was performed under a dry atmosphere of argon. Standard syringe techniques were applied for the transfer of dry solvents and air- or moisture-sensitive reagents. Styrene was passed over alumina to remove the inhibitor 4-*tert*-butylcatechol. The inhibitors in 4-VBC (TBC + ONP + 2-nitro-*p*-cresol) were removed *via* extraction with diethylether and 0.5% NaOH in water, evaporating the organic layer.^52,53^ Ultrapure water was obtained from a QPOD MilliQ system.

### Instrumentation

Nuclear magnetic resonance (NMR) characterization was carried out on a Bruker AVANCE HD nanobay console with a 9.4 T Ascend magnet (400 MHz) and a Bruker AVANCE III console with a 11.7 T UltraShield Plus magnet (500 MHz) equipped with a Bruker Prodigy cryoprobe, in chloroform (CDCl_3_). NMR spectra were recorded at 298 K unless otherwise specified. Chemical shifts are given in parts per million (ppm) with respect to tetramethylsilane (TMS, δ 0.00 ppm) as the internal standard for ^1^H NMR. Coupling constants are reported as *J* values in Hz. Peak assignment is based on 2D gDQCOSY, ^1^H-^13^C gHSQCED, and ^1^H-^13^C gHMBC spectra. Side group and end of chain signals separated from the bulk polymer ^1^H signal are only reported when observed with clear s/n ratio and no overlap with polymer peaks, and may be (in)visible on other NMR spectrometers or with different concentrations. The degree of polymerization was determined by comparing the integral of PEG (3.64) to PS (6.82–6.19) and 4-VBC (4.60–4.40). Gel permeation chromatography (GPC) equipped with PL gel 5 μm mixed D column calibrated for polystyrene (580 to 377,400 g/mol) was carried out on a Shimadzu instrument with THF as the eluent using differential refractive index and UV (254 nm) detectors. Transmission electron microscopy (TEM) was carried out on a JEOL TEM 1400 equipped with CCD camera at 60 kV. Samples were prepared by drop casting 5 μL of appropriately diluted samples on a carbon-coated Cu grid (200 mesh) and dried overnight at room temperature. Cryogenic TEM was carried out with a JEOL TEM 2100. Malvern Zetasizer nano S was used for dynamic light scattering (DLS) measurements equipped with a He–Ne laser of wavelength 633 nm. Fluorescence was measured on a Tecan Spark 200. All image analyses were carried out using ImageJ, available in a public domain http://fiji.sc/.^54^ A 300 W xenon light source was purchased from Asahi Spectra, Japan (MAX-303) with a wavelength range of 250–385 nm.

### Synthesis of Polymers

#### α-Methoxy-ω-2-bromo-2-phenylacetate-poly(ethylene glycol) (**1**)

α-Methoxy-ω-hydroxy-poly(ethylene glycol) (8.0 g, 4.0 mmol, 1 equiv) was dissolved in dry toluene (20 mL) and dried by azeotropic distillation. The polymer was subsequently dissolved in DCM (75 mL), after which DMAP (98 mg, 0.80 mmol, 0.2 equiv), EDC·HCl (2.30 g, 12.0 mmol, 3 equiv), and α-bromophenylacetic acid (2.58 g, 12.0 mmol, 3 equiv) were added. The mixture was stirred for 3 h at 21 °C, after which water (75 mL) was added to quench the reaction. The organic layer was then washed with saturated aqueous NaHCO_3_ and saturated aqueous NH_4_Cl solution, respectively. The organic layer was collected and dried with MgSO_4_ and concentrated under reduced pressure. The polymer was precipitated in ice-cold diethyl ether (2×) and dried *in vacuo* overnight to yield **1** as a white powder (8.70 g, 99%). ^1^H NMR (500 MHz, CDCl_3_) δ 7.58–7.48 (m, 2H, Ph ortho), 7.41–7.31 (m, 3H, Ph meta and para), 5.39 (s, 1H, C*H*-Ph), 4.40–4.25 (m, 2H, C*H_2_*CH_2_OC(O)), 3.72–3.68 (m, 2H, CH_2_C*H_2_*OC(O)), 3.64 (br s, 170H, PEG), 3.57–3.53 (m, 2H, CH_3_OC*H_2_*), 3.38 (s, 3H, C*H_3_*OCH_2_). ^13^C NMR (125 MHz, CDCl_3_) δ 168.2 (O*C*(O)), 129.3 (Ph ipso), 128.8 (Ph meta and para), 128.7 (Ph ortho), 128.0 (Ph ortho), 71.9 (CH_3_O*C*H_2_), 70.6 (PEG), 68.7 (CH_2_*C*H_2_OC(O)), 65.5 (*C*H_2_CH_2_OC(O)), 59.0 (*C*H_3_OCH_2_), 46.5 (*C*H-Ph). *R*_f_ 0.36 (MeOH/DCM, 1:9 v/v).

#### α-Methoxy-ω-2-phenyl-2-(phenylcarbonothioyl)thioacetate-poly(ethylene glycol) (**2**)

A Schlenk tube was flame-dried under vacuum, loaded with magnesium turnings (292 mg, 12.0 mmol, 3 equiv), and evacuated for 15 min and refilled with argon (3×). Afterward, dry THF (30 mL) and an I_2_ crystal were added. A solution of bromobenzene (1.88 g, 12.0 mmol, 3 equiv) in dry THF (30 mL) was added dropwise, and the mixture was stirred at 50 °C for 1 h. Carbon disulfide (914 mg, 12.0 mmol, 3 equiv) was added, after which the mixture was stirred for another 30 min at 50 °C. A solution of **1** (8.7 g, 4.0 mmol, 1 equiv) in dry THF (20 mL) was added, and the dark red solution was refluxed for 16 h. The reaction mixture was filtered to remove the leftover magnesium, concentrated under reduced pressure, and subsequently purified by column chromatography on silica gel using MeOH/DCM (gradient to 1:9 v/v) as the eluent. The polymer was precipitated in ice-cold diethyl ether (2×) and dried *in vacuo* overnight to yield **2** as a pink powder (4.3 g, 47%) as a pink solid. ^1^H NMR (400 MHz, CDCl_3_) δ 8.03–7.98 (m, 2H, CTA Ph ortho), 7.57–7.51 (m, 1H, CTA Ph para), 7.51–7.46 (m, 2H, Ph ortho), 7.41–7.31 (m, 5H, Ph meta and para & CTA Ph meta), 5.73 (s, 1H, C*H*-Ph), 4.42–4.23 (m, 2H, C*H_2_*CH_2_OC(O)), 3.71–3.67 (m, 2H, CH_2_C*H_2_*OC(O)), 3.64 (br s, 170H, PEG), 3.58–3.52 (m, 2H, CH_3_OC*H_2_*), 3.38 (s, 3H, C*H_3_*OCH_2_). ^13^C NMR (101 MHz, CDCl_3_) δ 225.9 (S*C*(*S*)Ph), 168.8 (O*C*(O)), 143.9 (CTA Ph ipso), 133.2 (Ph ipso), 132.8 (CTA Ph para), 129.0 (Ph meta), 128.9 (Ph para and ortho), 128.4 (CTA Ph meta), 126.9 (CTA Ph ortho), 71.9 (CH_3_O*C*H_2_), 70.6 (PEG), 68.8 (CH_2_*C*H_2_OC(O)), 65.3 (*C*H_2_CH_2_OC(O)), 59.0 (*C*H_3_OCH_2_), 58.8 (*C*H-Ph). *R*_f_ 0.33 (MeOH/DCM, 1:9 v/v).

#### α-Methoxy-poly(ethylene glycol)-*b*-poly(styrene-*co*-4-vinylbenzyl chloride) (PEG_44_-*b*-P(S_138_-*co*-4-VBC_18_)) (**3**)

A flame-dried Schlenk tube equipped with a stirring bar was loaded with styrene (1.7 mL, 14 mmol, 288 equiv), purified 4-vinylbenzyl chloride (0.30 mL, 1.92 mmol, 32 equiv), **2** (100 mg, 0.05 mmol, 1 equiv), and AIBN (1.7 mg, 0.010 mmol, 0.2 equiv). Anisole (0.18 mL, 1.6 mmol, 32 equiv) was added as the internal standard. The mixture was then degassed for 15 min with argon. The Schlenk tube was then immersed in a preheated oil bath of 70 °C, and the polymerization was monitored by ^1^H NMR spectroscopy. When the required length was obtained after roughly 3–5 h, the polymerization was terminated by removing the Schlenk tube from the oil bath and diluting the mixture with DCM. The mixture was then concentrated under reduced pressure. The polymer was precipitated with ice-cold methanol (3×) and dried *in vacuo* overnight to yield **3** as a pink solid (0.88 g, 44%). ^1^H NMR (400 MHz, CDCl_3_) δ 7.22–6.85 (m, PS arom. meta and para), 6.82–6.19 (m, PS arom. ortho), 4.60–4.40 (br s, Ph-C*H_2_*-Cl), 3.64 (br s, 176H, PEG), 3.38 (s, 3H, C*H_3_*OCH_2_), 2.36–1.64 (m, PS backbone C*H*), 1.61–1.07 (m, PS backbone C*H_2_*). ^13^C NMR (101 MHz, CDCl_3_) δ 145.9 (PS arom. ipso), 128.0 (PS arom. meta), 127.7 (PS arom. ortho), 125.7 (PS arom. para), 70.6 (PEG), 59.0 (*C*H_3_OCH_2_), 54.6 (Ph-*C*H_2_-Cl), 43.2 (PS backbone *C*H_2_), 40.5 (PS backbone *C*H). *M*_w_/*M*_n_ 1.13.

#### α-Methoxy-poly(ethylene glycol)-*b*-poly(styrene-*co*-4-vinylbenzyl acrylate) (PEG_44_-*b*-P(S_138_-*co*-4-VBA_18_)) (**4**)

A flame-dried Schlenk tube equipped with a stirring bar was loaded with K_2_CO_3_ (150 mg, 1.1 mmol, 27 equiv) in DMF (4 mL) and cooled to 0 °C. Acrylic acid (76 μL, 1.1 mmol, 27 equiv) was added, and the mixture was stirred for 1 h at 0 °C. Then, **3** (0.80 g, 40 μmol, 1 equiv) was added and the mixture was stirred for 3 h at 80 °C. Afterward, the reaction mixture was diluted with DCM and extracted with water (2×) and brine (2×), concentrated under reduced pressure, and precipitated in ice-cold methanol (3×). If required, the polymer can subsequently be purified by column chromatography on silica gel using MeOH/DCM (5:95 v/v) as the eluent. The polymer was filtered and dried overnight *in vacuo* to yield **4** as a pink powder (0.66 g, 82%). ^1^H NMR (400 MHz, CDCl_3_) δ 7.23–6.84 (m, PS arom. meta and para), 6.82–6.26 (m, PS arom. ortho and acrylate C*H*CH_2_), 6.23–6.08 (m, acrylate CHC*H*_2_), 5.89–5.74 (m, acrylate CHC*H*_2_), 5.21–5.01 (br s, Ph-C*H_2_*-acrylate), 3.64 (br s, 176H, PEG), 3.38 (s, 3H, C*H_3_*OCH_2_), 2.36–1.64 (m, PS backbone C*H*), 1.61–1.07 (m, PS backbone C*H_2_*). ^13^C NMR (101 MHz, CDCl_3_) δ 145.3 (PS arom. ipso), 128.0 (PS arom. meta), 127.7 (PS arom. ortho), 125.6 (PS arom. para), 70.6 (PEG), 59.0 (*C*H_3_OCH_2_), 66.1 (Ph-*C*H_2_-acrylate), 43.2 (PS backbone *C*H_2_), 40.5 (PS backbone *C*H). *M*_w_/*M*_n_ 1.13.

#### α-Methoxy-ω-2-bromo-2-methylpropanoate-poly(ethylene glycol) (**5**)

α-Methoxy-ω-hydroxy-poly(ethylene glycol) (5.0 g, 2.5 mmol, 1 equiv) was dried by co-evaporation with dry toluene to remove excess water. The polymer was dissolved in dry THF (20 mL) followed by addition of trimethylamine (1.04 mL, 7.50 mmol, 3 equiv) in a flame-dried Schlenk flask, and the mixture was cooled to 0 °C. α-Bromoisobutyryl bromide (616 μL, 5.00 mmol, 2 equiv) was added dropwise, and the mixture was stirred for 24 h, slowly warming to 21 °C. After the reaction, the mixture was filtered and subsequently concentrated under reduced pressure. The polymer was precipitated in ice-cold diethyl ether (3×) and dried *in vacuo* overnight to yield **5** as a white powder (4.84 g, 90%). ^1^H NMR (400 MHz, CDCl_3_) δ 4.33 (m, 2H, C*H_2_*CH_2_OC(O)), 3.76 (m, 2H, CH_2_C*H_2_*OC(O)), 3.65 (br s, 170H, PEG), 3.57–3.53 (m, 2H, CH_3_OC*H_2_*), 3.38 (s, 3H, C*H_3_*OCH_2_), 1.94 (s, 6H, C(C*H_3_*)_2_Br) ppm. ^13^C NMR (101 MHz, CDCl_3_) δ 171.6 (O*C*(O)), 71.9 (CH_3_O*C*H_2_), 70.5 (PEG), 68.7 (*C*H_2_CH_2_OC(O)), 65.1 (CH_2_*C*H_2_OC(O)), 59.0 (*C*H_3_OCH_2_), 55.7 (Br*C*(CH_3_)_2_), 30.8 (BrC(*C*H_3_)_2_).

#### α-Methoxy-poly(ethylene glycol)-*b*-polystyrene (PEG_44_-*b*-PS_178_) (**6**)

A Schlenk tube was flame-dried under vacuum, charged with CuBr (90 mg, 0.64 mmol, 3.2 equiv), evacuated for 15 min, and refilled with argon (3×). PMDETA (132 μL, 0.64 mmol, 3.2 equiv) in anisole (1.0 mL) was added, after which the mixture was stirred vigorously for 15 min followed by addition of styrene (10 mL, 87.2 mmol, 436 equiv) in anisole (0.5 mL). The mixture was then degassed for 15 min with argon. The mixture was cooled to 0 °C, and **5** was added (430 mg, 0.2 mmol, 1 equiv) in anisole (2 mL) followed by another 15 min of degassing. The tube was transferred to a pre-heated oil bath at 90 °C, and the reaction was monitored with ^1^H NMR. Upon attainment of the required molecular weight after roughly 3–5 h, the solution was diluted with DCM and extracted with aqueous EDTA (65 mM) (3×) until the blue color from Cu disappeared. The organic layer was collected, dried with MgSO_4_, and concentrated under reduced pressure. The polymer was precipitated with ice-cold methanol (3×) and dried *in vacuo* overnight to yield **6** as a white powder (4.04 g, 95%). ^1^H NMR (400 MHz, CDCl_3_) δ 7.24–6.86 (m, PS arom. meta and para), 6.58–6.28 (m, PS arom. ortho), 3.64 (br s, 176H, PEG), 3.38 (s, 3H, OC*H*_3_), 2.30–1.70 (m, PS backbone C*H*), 1.70–1.17 (m, PS backbone C*H_2_*), 1.00–0.93 (m, 6H, *b*). ^13^C NMR (101 MHz, CDCl_3_) δ 177.2 (O*C*(O)), 145.9 (PS arom. ipso), 128.0 (PS arom. meta), 127.7 (PS arom. ortho), 125.7 (PS arom. para), 70.6 (PEG), 59.0 (*C*H_3_OCH_2_), 47.1–41.6 (PS backbone *C*H_2_), 41.7 (Br*C*(CH_3_)_2_), 40.4 (PS backbone *C*H). *M*_w_/*M*_n_ 1.05.

### Synthesis of Probe

#### Prop-2-yn-1-yl-(4-methyl-2-oxo-2H-chromen-7-yl)carbamate (Propargyl-carbamate Masked Coumarin) (**7**)

7-Amino-4-methylcoumarin (99.2 mg, 0.567 mmol, 1 equiv) and pyridine (52.4 μL, 0.65 mmol, 1.15 equiv) were suspended in DCM (3 mL), after which propargyl chloroformate (68.1 μL, 0.70 mmol, 1.23 equiv) was added to the dark yellow suspension. The mixture was stirred at 0 °C for 16 h, after which it turned bright yellow. Then, 0.5 M HCl (40 mL) was added, and the mixture was subsequently filtered and washed with diethyl ether (2x). The resulting solid was dried *in vacuo* overnight to yield **7** as a yellow powder (119 mg, 82%). ^1^H NMR (500 MHz, CDCl_3_) δ 7.56 (d, *J* = 8.6 Hz, 1H, H9), 7.46 (d, *J* = 2.2 Hz, 1H, H6), 7.39 (dd, *J* = 8.7, 2.2 Hz, 1H, H8), 6.93 (s, 1H, N*H*), 6.23 (q, *J* = 1.3 Hz, 1H, H13), 4.82 (d, *J* = 2.5 Hz, 2H, H3), 2.56 (t, *J* = 2.4 Hz, 1H, H1), 2.49 (d, *J* = 1.3 Hz, 3H, H12). ^13^C NMR (125 MHz, CDCl_3_) δ 160.9 (C14), 159.2 (C7), 152.0 (C11), 151.9 (C4), 140.9 (C5), 125.4 (C9), 115.9 (C10), 114.4 (C8), 113.5 (C13), 106.1 (C6), 77.2 (C2), 75.4 (C1), 52.9 (C3), 18.6 (C12). *R*_f_ 0.32 (MeOH/DCM, 1:9 v/v).

### General Preparation of Polymersomes

Modified from a previous report,^55^ a general procedure is described: PEG-*b*-PS polymer **6** (10 mg) was dissolved in a mixture of THF and 1,4-dioxane (1 mL, 4:1 v/v) in a 15 mL vial with a magnetic stir bar. After dissolving the polymer for 0.5 h at 21 °C, a syringe pump equipped with a syringe and a needle was used to deliver ultrapure water with a rate of 1 mL/h for 0.5 h *via* a rubber septum while vigorously stirring the mixture (900 rpm). Upon finishing the water addition, 8.0 mL of ultrapure water was added to the suspension to rapidly quench the polymersomes. The polymersomes were spun down using a centrifuge (10 min, 13.000 rpm) and washed with ultrapure water a total of three times.

### General Preparation of Cross-Linked Polymersomes

Cross-linkable polymer **4** (10 mg) was dissolved in a mixture of THF and 1,4-dioxane (1 mL, 4:1 v/v) in a 15 mL aluminum foil-wrapped vial with a magnetic stirring bar. 0.028 equiv compared to the number of 4-VBA groups in the polymer was added from a stock solution. For our polymer, this was 40 μL of a 2.4 mg/mL solution of Irgacure in THF/1,4-dioxane 4:1 (v/v). After dissolving the polymer for 0.5 h at 21 °C, a syringe pump equipped with a syringe and a needle was used to deliver ultrapure water with a rate of 1 mL/h for 0.5 h *via* a rubber septum, while vigorously stirring the mixture (900 rpm). Upon finishing the water addition, the polymersome mixture was degassed by flushing the solution with argon for 5 min, after which the vial is immediately placed under a UV lamp to start the cross-linking process. The polymersome suspension was irradiated for 5 min at 60% power with the full wavelength range. 8.0 mL of ultrapure water was then added to quench the polymersomes. The polymersomes were spun down using a centrifuge (10 min, 13.000 rpm) and washed with ultrapure water a total of three times.

### General Preparation of Cross-Linked Polymersomes with Functional Handles

A similar procedure as above was used except substituting cross-linkable polymer **4** (10 mg) for cross-linkable polymer **4** (9 mg) and handle-PEG-*b*-PS (1 mg).^56^

### General Procedure of Resuspending Cross-Linked Polymersomes in Organic Solvent

The cross-linked polymersomes in ultrapure water were spun down using a centrifuge (10 min, 13.000 rpm) after which the supernatant was removed. The pellet was then resuspended in the organic solvent and washed with organic solvent a total of three times.

### Click Reaction on DBCO-PEG-*b*-PS Cross-Linked Polymersomes

2 × 300 μL of THF suspended cross-linked polymersomes with 10% DBCO-handle were added to two Eppendorf tubes. To one of these tubes, 0.50 mg of 3-azido-7-hydroxycoumarin^57^ was added. The polymersomes in the other tube were washed with THF five times and washed back into water. After this, 0.50 mg of 3-azido-7-hydroxycoumarin was added to the second tube. Both tubes were shaken using an Eppendorf thermomixer for 1 h. The fluorescence was measured using an excitation wavelength of 485 nm (bandwidth 20 nm) and detection wavelength of 535 nm (bandwidth 20 nm).

### General Preparation of Cross-Linked Polymersomes with Holes

A similar procedure to the general preparation of cross-linked polymersomes was used, except substituting cross-linkable polymer **4** (10 mg) for cross-linkable polymer **4** (10 – *x* mg) and PEG-*b*-PS (*x* mg). The number *x* ranges from 1 to 8, forming the 90%–20% cross-linked polymersomes.

### Preparation of Pt NPs with PVP Coating

K_2_PtCl_4_ solution in water (4 mL, 20 mM) was added into PVP (40 mg) followed by 48 h stirring. After that, *L*(+)-ascorbic acid (35 mg) in water (1 mL) was added into the solution. The resulting solution was sonicated at 21 °C for 1 h to obtain Pt NPs.

### General Preparation of Cross-Linked Polymersomes with Pt NPs

Encapsulation of PtNP was performed using the general method for preparation of cross-linked polymersomes, with the modification of adding a dispersion of Pt NPs in ultrapure water (0.5 mL) instead of only ultrapure water. To remove the excess PtNPs, the polymersomes were washed three times using a 0.22 μm centrifugal filter (10 min, 13.000 rpm) after the standard washing procedure.

### Catalytic Essay Using (Encapsulated) Pt NPs

Cross-linked MeO-PEG_44_-*b*-P(S_141_-*co*-4-VBA_18_)_159_ polymersomes (100% and 60%) and free Pt NPs were diluted 50× from regular concentration in a well plate (100 μL). **7** (1 mg) was added, and the plate was shaken for 15 min at 37 °C. The samples were then immediately measured on a plate reader. Excitation was performed using a filter for 365 nm (20 nm bandwidth), and emission was measured with a filter for 440 nm (20 nm bandwidth).

## Results and Discussion

### Synthesis of the Cross-Linkable Polymer

**Figure 2:**
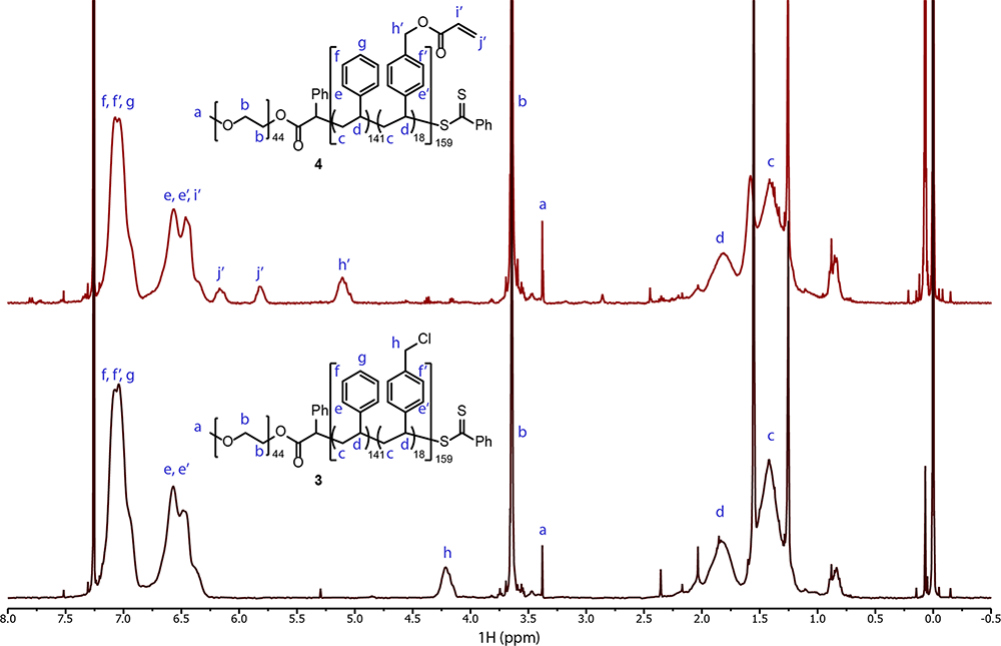
Characterization of PEG_44_-*b*-P(S_138_-*co*-4-VBC_18_) **3** and PEG_44_-*b*-P(S_138_-*co*-4-VBA_18_) **4** polymers *via*^1^H-NMR (400 MHz, CDCl_3_).

**Scheme 1:**
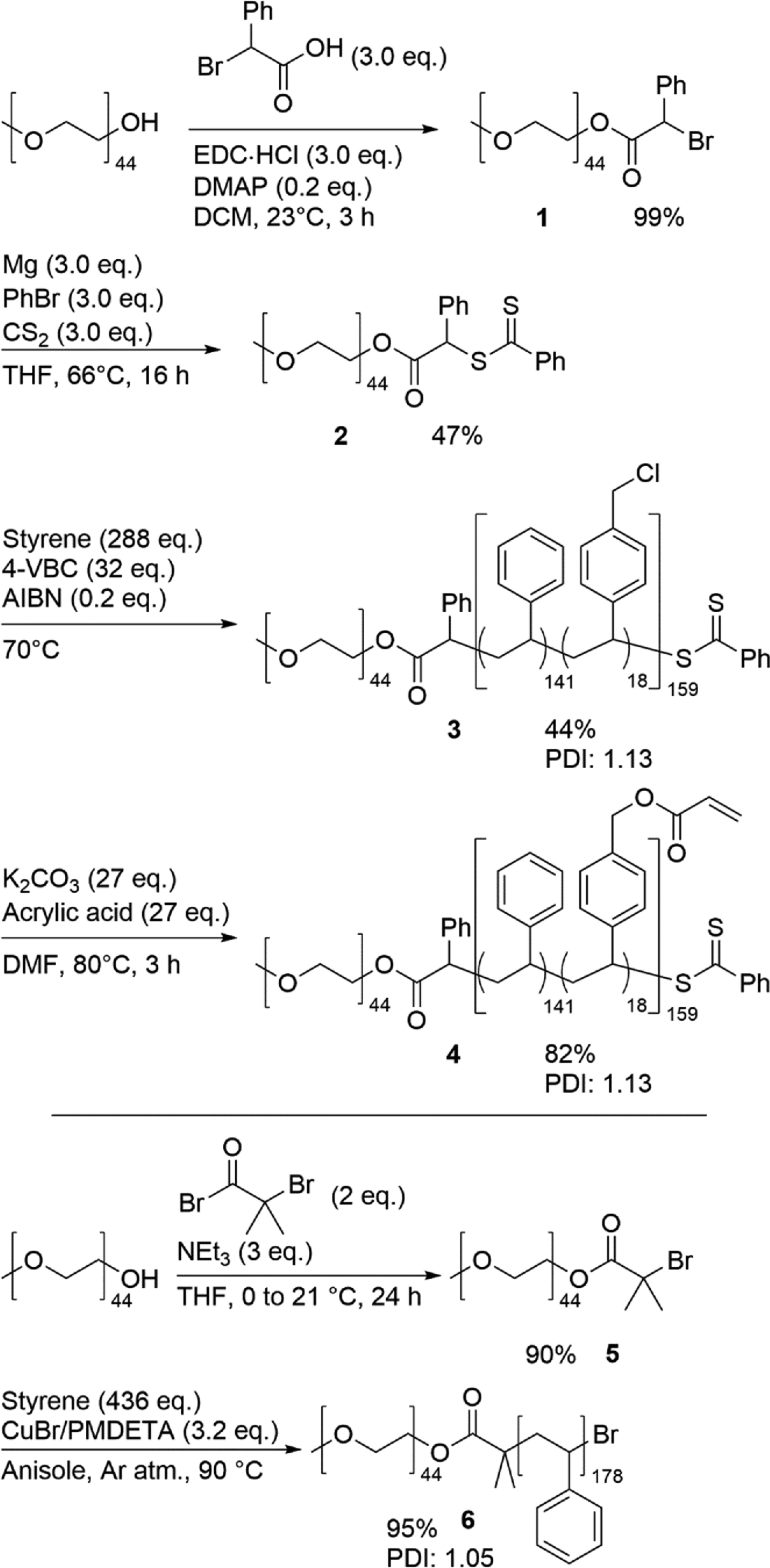
Strategy for the Synthesis of PEG-*b*-P(S-*co*-4-VBA) and PEG-*b*-PS Polymers

The cross-linkable polymer PEG_44_-*b*-P(S_138_-*co*-4-VBA_18_) **4** (Scheme 1) was synthesized *via* reversible addition-fragmentation chain transfer (RAFT) polymerization. Because direct incorporation of an acrylic moiety into the hydrophobic polystyrene block would lead to cross-linking during the synthesis of the block copolymer, we decided to incorporate this group later into the synthesis. To start, a chain transfer agent (CTA) compound was prepared, first *via* a condensation reaction of MeO-PEG_44_-OH with α-bromophenylacetic acid to give **1**, after which the final MeO-PEG_44_-CTA **2** was made by a nucleophilic substitution of the product of a Grignard reaction between carbon disulfide, phenyl bromide, and magnesium. Immediately after purification, **2** was then used for the copolymerization of styrene and 4-vinylbenzyl chloride (4-VBC) (9:1) using RAFT to create the pink solid (PEG-*b*-P(S-*co*-4-VBC)) **3** with an average hydrophobic block length of 159. This length was confirmed by ^1^H-NMR, and GPC analysis showed a PDI of 1.13. RAFT was chosen due to the incorporation of 4-VBC as it would be incompatible with ATRP polymerization. Then, a nucleophilic substitution using potassium carbonate and acrylic acid in DMF introduced the acrylate groups onto the hydrophobic block benzyl chloride to yield the pink final product **4** (PEG-*b*-P(S-*co*-4-VBA)). Full substitution after 3 h was confirmed *via*^1^H-NMR by the shift of the benzylic protons and the inclusion of broad vinyl proton signals (Figure 2). Quenching this reaction on time proved essential to the synthesis, as long reaction times would slowly cause the ester bond to cleave under the reaction conditions, breaking the bridge between the hydrophilic and hydrophobic blocks. A reaction time of 3 h was enough to fully substitute the polymer while avoiding this side reaction, as was shown by GPC analysis, which was virtually unchanged from the starting material. In cases of cleavage, purification by column chromatography was used to purify the polymer. Finally, in order to test compatibility of the cross-linkable polymer with non-cross-linkable polymer, regular MeO-PEG-*b*-PS was synthesized according to the previous literature.^58^ MeO-PEG-OH was reacted in a condensation reaction with α-bromoisobutyryl bromide to give the ATRP initiator **5**, after which styrene was polymerized to create the white block copolymer PEG-*b*-PS **6** with an average length of 178 and PDI of 1.05.

### Self-Assembly of the Polymersome

Various lengths and compositions of PEG-*b*-P(S-*co*-4-VBA) were initially synthesized as the difference in molecular structure could influence the self-assembly process, with **4** forming a monodisperse distribution of polymersomes. After the RAFT polymerization reaction to synthesize **4**, it was determined *via*^1^H-NMR that roughly 14% of the hydrophobic block consisted of 4-VBC. This ratio was determined to be optimal for later self-assembly of the system, as a lower incorporation would not be sufficient to cross-link the polymersome structure, and a higher incorporation would lead to the polymer self-assembling into other structures than desired. Similarly, the length of the block copolymer had to be optimized, as the length of 180 for the hydrophobic block commonly used in our group^22^ resulted in different morphologies being formed. The slightly shorter length of 159 resulted in the desired uniform formation of polymersome structures.

Several methods have been reported to prepare polymersomes out of block copolymers.^59,60^ For the cross-linkable polymersomes, we used the co-solvent approach, where the polymers are initially dissolved in an organic solvent mixture, after which water is slowly introduced to the system. This results in self-assembly by phase separation of the hydrophobic block due to the slow increase in hydrophilicity of the mixture.^61^ During the self-assembly process, a minimal amount of Irgacure 2959 was added. By testing a range of concentrations, the minimum amount of initiator required to create fully integer polymersomes was found to be 0.028 equiv compared to the number of 4-VBA groups in the polymer (Figures S1–S11). After dissolving polymer **4** and Irgacure 2959 in 1 mL of organic solvent (4:1 THF/1,4-dioxane), 0.5 mL of ultrapure water was added slowly at 1 mL/h, inducing self-assembly into spherical polymersomes. After the self-assembly, the mixture was irradiated for 5 min with UV light. Finally, the cross-linked PEG-*b*-P(S-*co*-4-VBA) polymersomes were quenched by addition of 8 mL ultrapure water, after which the suspension was transferred to a centrifuge tube. The suspension was purified with ultrapure water 3× by centrifugation to remove organic solvent and finally suspended in 1 mL ultrapure water as a 10 mg/mL suspension. The integrity of the polymersomes after cross-linking was verified by transmission electron microscopy (TEM) and cryo-TEM.

**Figure 3:**
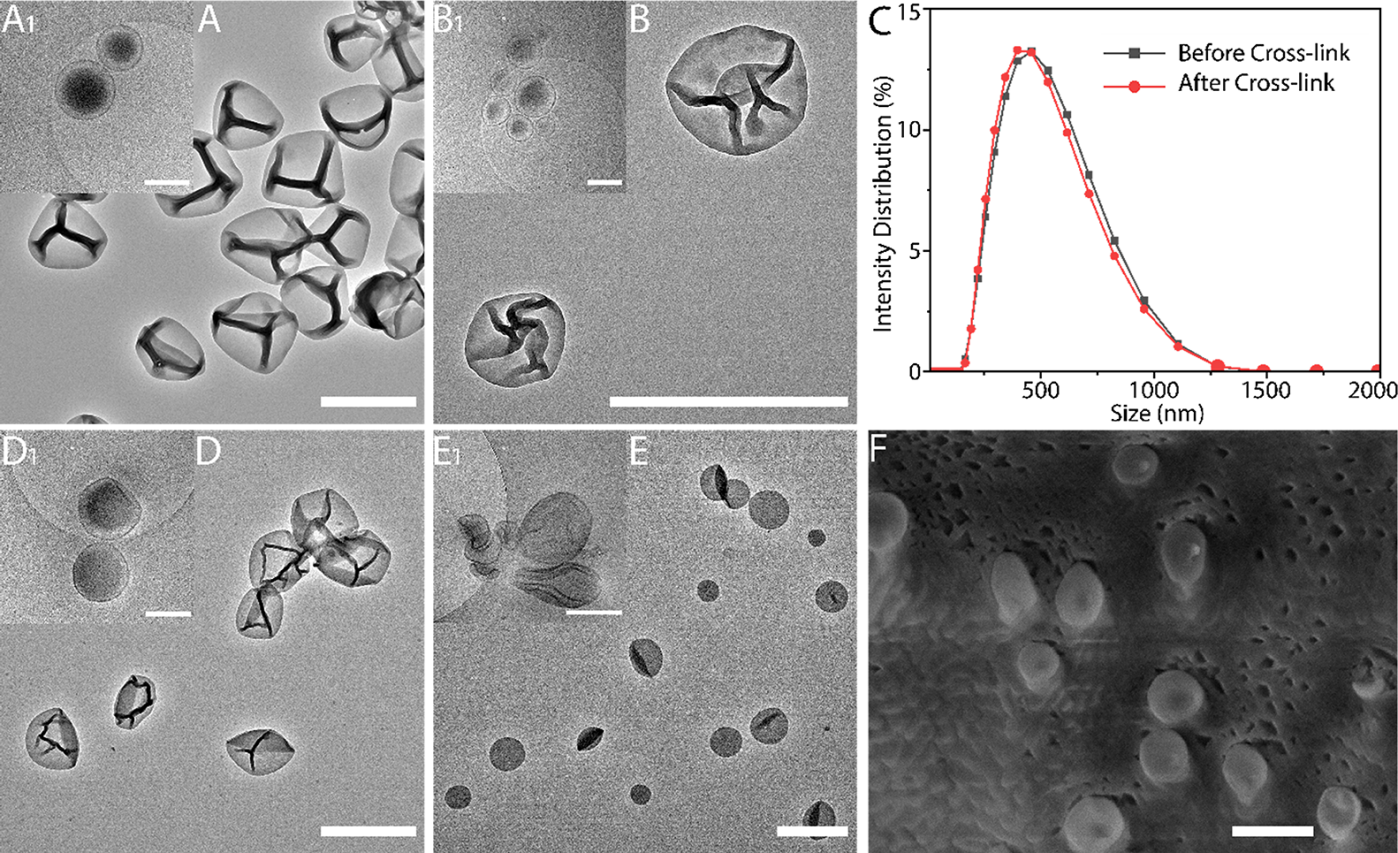
TEM image and cryo-TEM image of MeO-PEG_44_-*b*-P(S_141_-*co*-4-VBA_18_)_159_ polymersomes in water before (A,A1) and after (B,B1) cross-linking. (C) Size distribution of MeO-PEG-*b*-P(S_141_-*co*-4-VBA_18_)_159_ polymersomes before and after cross-linking from DLS data. TEM image and cryo-TEM image in THF (D,D1) and DCM (E,E1). Cryo-TEM images were made after resuspension in water. (F) Cryo-SEM image of spherical MeO-PEG-*b*-P(S_141_-*co*-4-VBA_18_)_159_ polymersomes. Scale bar: 1000 nm (TEM and Cryo-SEM), 500 nm (Cryo-TEM).

Before cross-linking, a neat distribution of polymersomes was obtained (Figure 3A). After irradiation with UV light, the cross-linked particles were analyzed and shown to have retained their characteristic polymersome shape (Figure 3B). Cryo-TEM shows the undisturbed polymersome structure, both before and after cross-linking (Figure 3A1,B1). Consequently, the polymersomes did not suffer from structural damages upon UV radiation and the cross-linking process. The average diameters of the spherical polymersomes were determined by dynamic light scattering (DLS) and were around 460 nm, showing no significant change upon cross-linking (Figure 3C).^62^ The samples remained cloudy upon transfer to various organic solvents, and the structures were confirmed with TEM, both for polymersome suspensions miscible (THF, Figure 3D) and immiscible (DCM, Figure 3 E) with water. We noticed that the shape of the structures dried from THF on TEM remained similar to those dried from water, while those dried from DCM showed a coffee bean-like structure and change in their shape. Cryo-TEM confirmed a difference in shape as the THF polymersomes were still round (Figure 3D1) while those from DCM had folded (Figure 3E1) into a disc shape.^55^ Since DCM is immiscible with water, this may cause a situation in which the disc-shape is thermodynamically favorable over the spherical polymersome shape. To get a better view of these shapes, we analyzed them at different viewing angles *via* cryo-TEM (Figures S12–S14). Since TEM and cryo-TEM are 2D techniques, we used cryo-SEM to further confirm the structures and determine their overall 3D shape. Similar spherical and slightly dented morphologies are observed by cryo-SEM (Figure 3F) (Figures S15 and S16).

### Solvent and Stability Tests

Having shown that the polymersomes are stable in basic organic solvents, we were interested in whether they would retain their shape over a longer period of time or whether the acrylate group would wear down, diminishing the cross-link density and solvating the polymersome. In order to test whether the cross-linked polymersomes are stable over time, they were suspended in various (organic) solvents. Their structures were validated at different points in time. The results of these experiments are shown in Figure 4 (Figures S17–S36). As a control, un-irradiated polymersomes were also washed with organic solvent and immediately dissolved, leaving no nanostructure to be observed.

**Figure 4:**
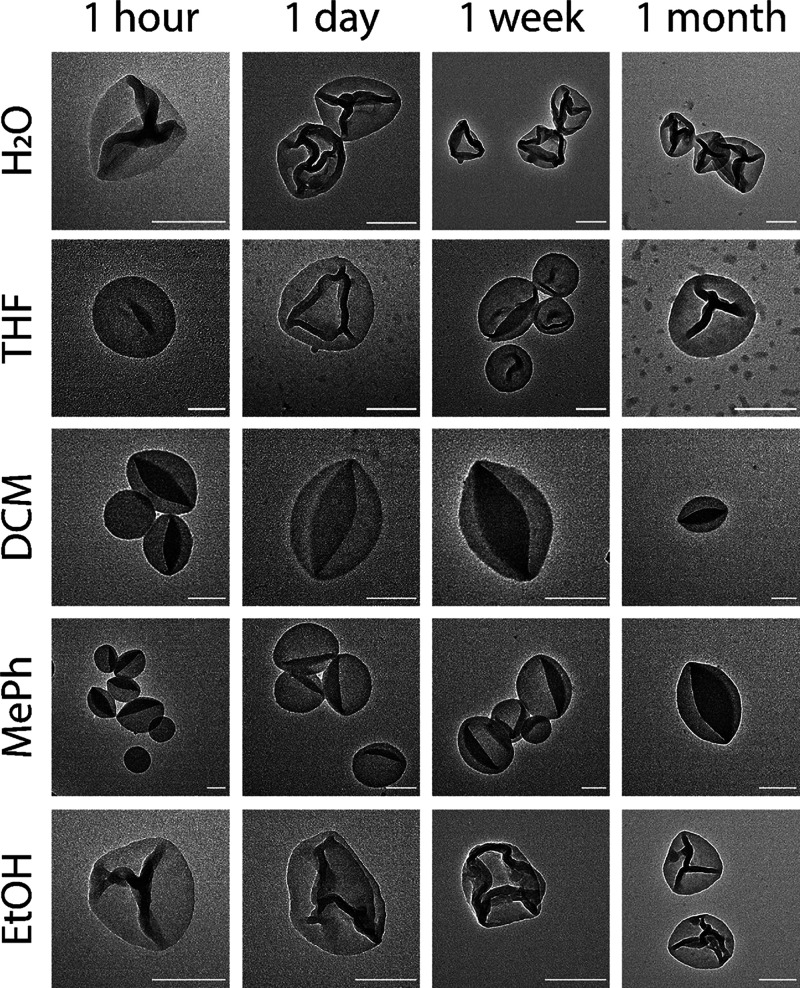
Representative TEM images of MeO-PEG_44_-*b*-P(S_141_-*co*-4-VBA_18_)_159_ polymersomes in water, THF, DCM, toluene, and ethanol over various periods of time. Samples have been measured 1 h, 1 day, 1 week, and 1 month after resuspension in the respective solvent. No significant changes in the volume and shape of the polymersomes were detected. Scale bar: 250 nm.

Toluene, DCM, ethanol, and THF were chosen as solvents to test the stability of the cross-linked polymersomes, together with water as a control.^63^ They were chosen because they represent the four commonly used classes of organic solvents: hydrocarbons, chlorinated solvents, alcohols, and ethers.^64^ Again, we found that toluene, non-miscible with water, showed a similar structure to those dried from DCM, while those in ethanol had a similar dried structure to those from water. From the TEM, we were able to see no significant change in the volume or shape of the polymersomes. DLS measurements of the polymersomes support this as the size distributions stay the same over time and no clustering was observed (Figures S37–S40). The polymersomes that were dispersed in toluene did form a small number of aggregates immediately, as evidenced by the DLS size distribution (Figures S41 and S42). The difference in density between toluene and the water inside the polymersomes could be the cause of the aggregates forming. Toluene is less dense than water, which may, during the solvent change, cause the highly concentrated polymersomes in water to cluster and aggregate. We also found that during the solvent exchange, polymersomes tended to stay afloat in DCM while instantly sinking in toluene, indicating the presence of water in these particles. From this experiment, we concluded that the cross-linked polymersomes are stable in a variety of solvents for a longer period of time.

### Functional Handle Incorporation

Having shown that the polymersomes are stable in basic organic solvents, we were interested in incorporating functional handles into the system. In our previous work, we established a modular approach toward functionalization of non-cross-linkable polymersomes.^56^ We hypothesized that a relatively low incorporation of these handles would be able to remain inside the structure if the cross-link density was high enough. To test this, a cross-linked polymersome was synthesized using 90% MeO-PEG_44_-*b*-P(S-*co*-4-VBA)_159_ as the main component, adding 10% DBCO-PEG_44_-*b*-PS_189_ polymer. This DBCO-group can be reacted with 3-azido-7-hydroxycoumarin to become fluorescent.^65^ The decorated polymers are slightly longer than unreactive polymers to ensure availability on the surface. Self-assembly of this system is the same as described above. Spherical polymersome structures were confirmed *via* cryo-TEM (Figure S43). To confirm the presence of the DBCO moiety, two samples of 50× diluted polymersomes were reacted with 3-azido-7-hydroxycoumarin in THF overnight. One sample was washed with THF five times, while the other was reacted with 3-azido-7-hydroxycoumarin without washing. The cycloaddition reaction was shown to have succeeded by fluorescence spectroscopy, showing a significant increase in fluorescence compared to the blank. No significant difference was found between the two samples, indicating that DBCO-PEG-*b*-PS remained within the polymersome structure (Figure S44).

### Porous Polymersomes

Although we found that 10% incorporation of non-cross-linkable polymer led to stable structures, we presumed that lowering the amount of cross-link polymer **4** and increasing the amount of PEG-*b*-PS **6** could lead to a porous structure. Despite the similarity in structure between **4** and **6**, small differences in polarity can lead to immiscibility between polymers.^66^ In this case, we hypothesized that by using a mixture of **4** and **6** for the preparation of our polymersomes, the more nonpolar PS block of **6** and the more polar P(S-*co*-4-VBA) block of **4** would readily undergo phase separation upon formation of the bilayer. This implies that using various amounts of **6**, phase-separated PS domains would be dispersed through the membrane of the polymersome, which could then be readily extracted from the matrix by washing with organic solvent.^67^ Because only **6** should dissolve in organic solvent, the structural integrity of the resulting polymersomes would be preserved when **6** is a minor component. This implies that the generated permeability could be controlled simply by changing the ratio between **4** and **6** during self-assembly.

**Figure 5:**
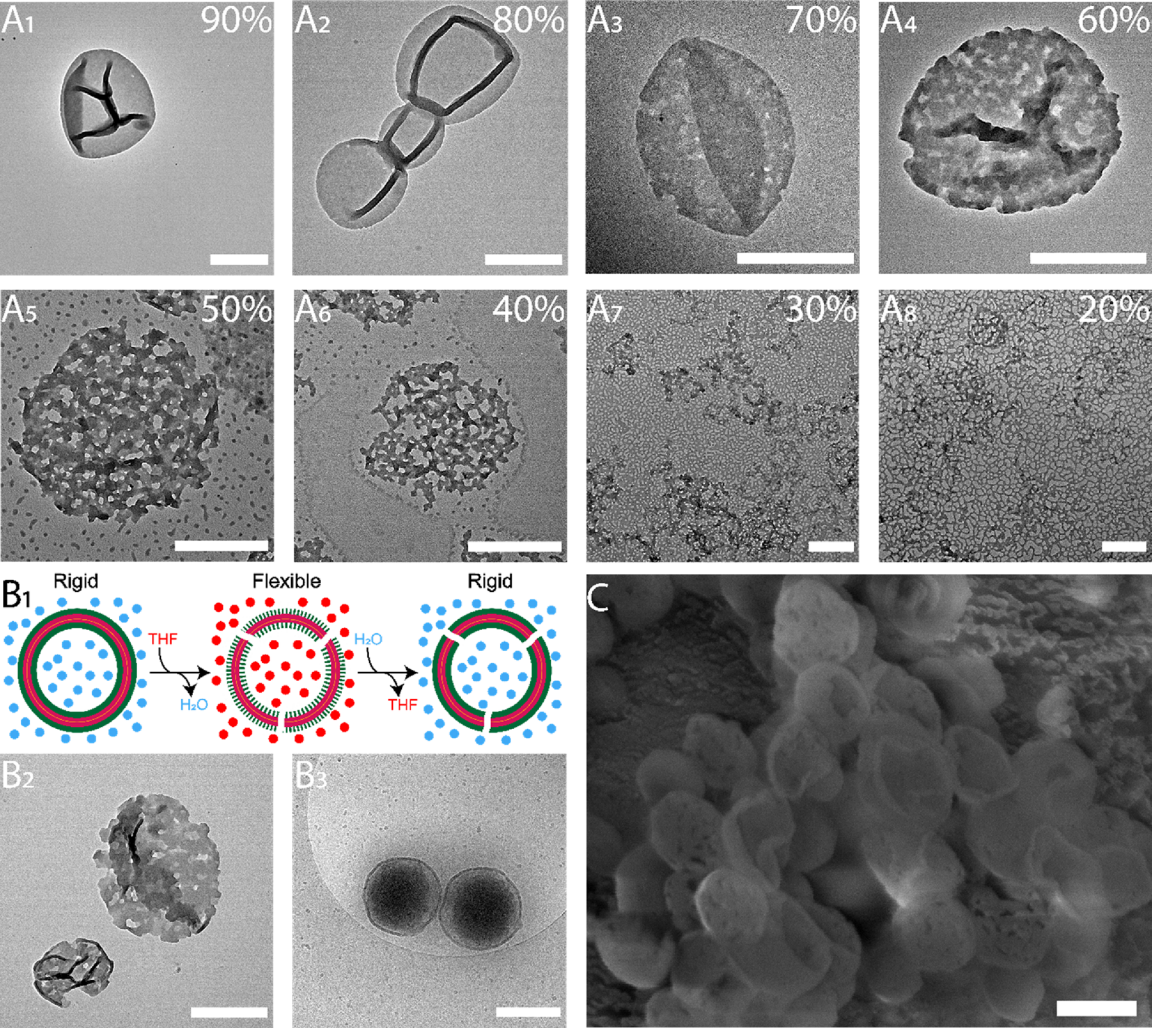
TEM images (A) of porous polymersomes from THF made from MeO-PEG_44_-*b*-P(S_141_-*co*-4-VBA_18_)_159_ and MeO-PEG_44_-*b*-PS_179_ mixtures ranging from 90% to 20% (A_1_–A_8_) cross-linkable polymer. The structure of the membrane in THF is retained in water due to the membrane becoming inflexible upon addition of water (B_1_). TEM (B_2_) and cryo-TEM (B_3_) images of the 60% polymersome after resuspension in water. Cryo-SEM (C) image of porous 60% MeO-PEG-*b*-P(S_141_-*co*-4-VBA_18_)_159_ polymersomes. Scale bar: 500 nm.

To examine the validity of our idea, we prepared multiple homogeneous mixtures of PEG-P(S-*co*-4-VBA) **4** and PEG-*b*-PS **6** in 4:1 THF/1,4-dioxane with various mixing ratios (90–20%, corresponding to the weight percent of **4**) and were all successfully self-assembled into polymersomes (Figures S45–S60). After washing 3× with THF, porous nanostructures were obtained (Figure 5A). As expected, when 90% of the structure consisted of the cross-linkable **4**, no porous structures were obtained because the network is strong enough to trap the PEG-*b*-PS (Figure 5A1). At lower amounts though, pores have started to become visible on TEM. We have measured the size of the pores from the TEM images and calculated the average diameter (Table S1). At 80%, a fraction of polymersomes started to show small specs in their structure, indicating that small holes are formed (Figure 5A2). With even higher incorporation of **6** in the structure, more observable holes were found, with up to a 60/40 mixture of **4** and **6** still forming nanostructures that retained the polymersome shape (Figure 5A4). We presume that these holes in the polymersomes are areas previously occupied by domains of **6**, which left pores upon disassembly due to resolvation. When the ratio is tipped even further toward **6**, the polymersome structure can still be observed, but the particles have become more like a mesh (Figure 5A5,A6), and going even further leaves only a cross-linked grid instead of distinguishable particles (Figure 5A7,A8). We observe that the pore sizes increase seemingly exponentially with the increase of **6** incorporation (Figure S61). For 20–40% incorporation, the pores in the membrane roughly had the same size per sample. The average sizes were 14.7, 19.5, and 31.0 nm, respectively. For 50 and 60% however, we noticed a larger distribution of pore sizes, indicating that the polymers form clusters during the self-assembly. The average sizes were 43.1 and 78.6 nm, respectively. These observations suggest that the optimal mixing ratio between the two block copolymers is at 60/40 **4**/**6**, so that the resulting polymersomes possess evenly distributed pores in the matrix.

To prove that the removal of **6** introduced permeability, we analyzed the structures further. A sample containing 60% of **4** was prepared in water and subsequently washed with THF to wash out **6** and create the pores. The addition of organic solvent makes the membrane flexible again, which could have an influence on the shape of the nanostructure. To confirm this is not the case, the sample was re-dispersed in water, instantly turning the membrane glassy and thus retaining the shape it had in THF (Figure 5B1).^68^ The porous structure was still visible on TEM (Figure 5B2), and cryo-TEM confirmed the structures were still spherical (Figure 5B3). Finally, cryo-SEM was used to get a more detailed image of the pores created, and holes can clearly be seen in the membrane (Figure 5C) (Figures S62–S65) compared to the fully cross-linked polymersomes (Figure 3F).

**Figure 6:**
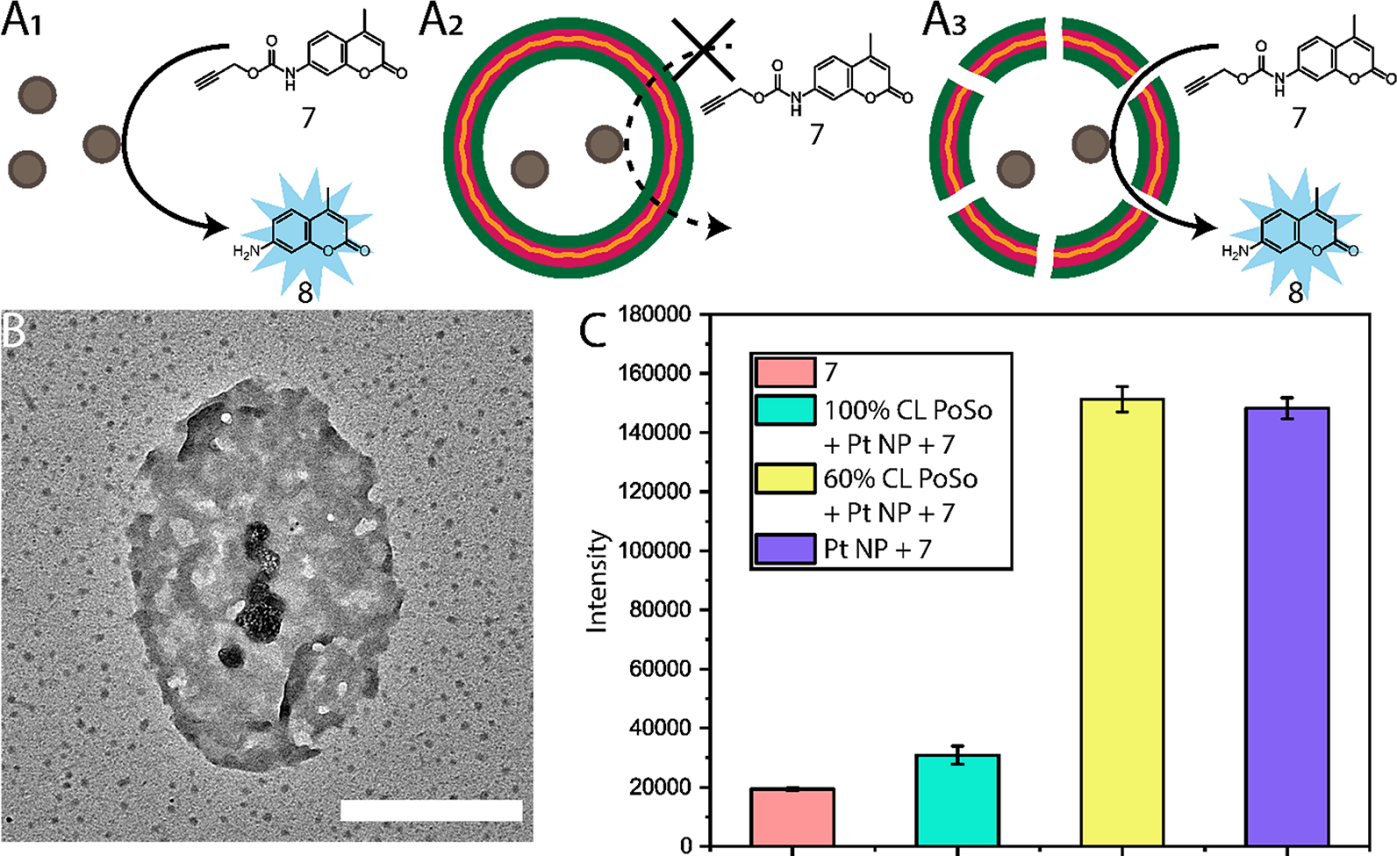
Free nanoparticles in solution can catalyze the reaction (A_1_), while encapsulated nanoparticles are not available to the substrate (A_2_). In porous polymersomes, these are available making them able to catalyze the reaction (A_3_). TEM image (B) of porous polymersomes from THF made from a 60% MeO-PEG_44_-*b*-P(S_141_-*co*-4-VBA_18_)_159_ and 40% MeO-PEG_44_-*b*-PS_179_ mixture containing platinum nanoparticles. Fluorescence essay (C) of these reactions comparing **7** without additions, with addition of free Pt NPs, 100% cross-linked polymersomes, and 60% cross-linked polymersomes containing Pt NPs. Scale bar: 500 nm.

To prove that the pores introduced selective permeability, we performed a catalytic reaction with them. Palladium and platinum nanoparticles are known to be able to depropargylate various compounds, so we intended to use this system to deprotect a propargyl-carbamate masked coumarin **7**.^69^ This would result in the formation of a fluorescent coumarin **8**, which could be followed by fluorescence spectroscopy. Free Pt NPs in solution would readily provide this reaction while a fully cross-linked polymersome with Pt NPs encapsulated would not be available as the substrate cannot enter the reactor. The molecular substrate can however enter the porous polymersomes, allowing the platinum to depropargylate the structure (Figure 6A). First, we made PVP-capped platinum nanoparticles (Pt NPs) as previously reported,^58^ with an average diameter of 60 nm as confirmed by DLS. We then encapsulated these in the inner compartment of the porous polymersomes (60%). This was done by adding a suspension of Pt NPs into the water during the self-assembly process and subsequently removing all platinum that was not encapsulated by centrifugal washing. The encapsulated nanoparticles could not escape the polymersome due to their size and remained inside after thorough washing with THF, as can be seen on TEM (Figure 6B). This confirmed that the polymersomes remained intact. After having demonstrated that the permeable polymersomes with an encapsulated catalyst can be prepared, we pursued to use these nanoreactors in a catalytic reaction. As shown in Figure 6C, the fully cross-linked polymersome with Pt NPs showed virtually no catalytic activity as the fluorescence is similar to **7** in contrast to free Pt NPs and porous nanoreactors. These final two show similar results after 15 min of incubation at 37 °C, showing a fivefold increase in fluorescence, suggesting the full availability of the platinum in the porous polymersomes. These results were in compliance with blank experiments of all separate components (Figure S66). This indicates that after washing out **6**, the voids left in the structure permit transmembrane diffusion of small substrates.

## Conclusions

In conclusion, we have developed cross-linked polymersomes and successfully introduced controlled permeability in the membrane *via* solvation of non-cross-linkable block copolymers that are embedded in polymersome membranes. For this, a polymer capable of cross-linking, PEG-*b*-P(S-*co*-4-VBA) was synthesized *via* RAFT polymerization and subsequent introduction of the active group onto the hydrophobic block. Covalently cross-linked polymersomes were prepared on the basis of a photo-initiated polymerization reaction of acrylate groups within the membrane, requiring no external additives save for a minimal amount of photoinitiator. A general co-solvent method ensured smooth self-assembly of the functionalized polymers into monodisperse polymersomes. The cross-linking reaction was successfully performed by irradiation with UV light. The stability of the cross-linked polymersomes was confirmed over the period of a month for a variety of different solvents. Inclusion of a functional DBCO group was demonstrated, which was subsequently reacted with the fluorescent azidocoumarin, proving the availability of the functional group in this system. By mixing PEG-*b*-P(S-*co*-4-VBA) with non-cross-linkable PEG-*b*-PS in various ratios, polymersomes with different levels of porosity were made and shown to retain their polymersome structure. The relevance of these porous cross-linked polymersomes was demonstrated by encapsulating Pt NPs, providing them with a protective shell, and performing a depropargylation reaction with them with a similar efficiency to free catalysts in solution.

## Supporting Information Available

TEM, cryo-TEM and cryo-SEM images of polymersomes, DLS data of polymersomes, fluorescence data and NMR of all synthesized compounds (PDF)
