# Supplementary material for: Photo-Cross-Linking Polymersome Nanoreactors with Size-Selective Permeability
Source: Macromolecules. 2022 Jun 30;55(13):5744–55. doi: 10.1021/acs.macromol.2c00248 (PMC9281476; doi:10.1021/acs.macromol.2c00248)
Supplement: Supplementary file 1 — ma2c00248_si_001.pdf [file ma2c00248_si_001.pdf]

Supporting Information

Photo-Cross-linking Polymersome Nanoreactors with Size-Selective Permeability

Sjoerd J. Rijpkema, Rik van Egeraat, Wei Li and Daniela A. Wilson*

Institute for Molecules and Materials, Radboud University, Heyendaalseweg 135, 6525 AJ, Nijmegen, The Netherlands

**e-mail:* d.wilson@science.ru.nl

Table of contents

1. Materials, Instrumentation and Methods 3
2. Synthesis of block co-polymers 4
3. TEM images of Irgacure 2959 Optimisation 10
4. Cryo-TEM images of Polymersomes under Various Angles 16
5. Cryo-SEM image of Cross-Linked Polymersomes 17
6. TEM images of Polymersome Solvent Stability Tests 18
7. DLS data of Polymersome Solvent Stability Tests 28
8. Cryo-TEM images of DBCO-containing Cross-Linked Polymersomes 31
9. Fluorescence data of DBCO + 3-azido-7-hydroxycoumarin Click Reaction 32
10. TEM images of Polymersomes with Holes 33
11. Pore Size of Cross-Linked Polymersomes with Holes 41
12. Cryo-SEM image of Cross-Linked Polymersomes with Holes 43
13. Fluorescence data of Pt NP + Propargyl-carbamate Masked Coumarin Reaction 45
14. NMR and GPC spectra of synthesized polymers 46
15. References 60

1. Materials, instrumentation and methods

All PEG polymers with different functional end groups were obtained from AV Chemistry. All other reagents and chemicals were purchased from Sigma Aldrich, unless and otherwise stated. All other reagents were obtained from commercial sources and were used without purification unless otherwise stated. Solvents were dried by passing over activated alumina columns in a MBraun MB SPS800 under a nitrogen atmosphere and stored under argon. Reactions were carried without the need for an inert atmosphere unless stated otherwise, in which case the reaction was performed under a dry atmosphere of argon. Standard syringe techniques were applied for the transfer of dry solvents and air- or moisture sensitive reagents. Styrene was passed over alumina to remove the inhibitor 4-tert-butylcatechol. The inhibitors in 4-VBC (TBC + ONP + 2-Nitro-p-cresol) were removed via extraction with diethylether and 0.5% NaOH in water, evaporating the organic layer. [1, 2] Ultrapure MilliQ water was obtained from QPOD MilliQ system.

Nuclear Magnetic resonance (NMR) characterization was carried out on a Bruker AVANCE HD nanobay console with a 9.4 T Ascend magnet (400 MHz) and a Bruker AVANCE III console with a 11.7 T UltraShield Plus magnet (500 MHz) equipped with a Bruker Prodigy cryoprobe, in chloroform ($CDCl_3$). NMR spectra were recorded at 298 K unless otherwise specified. Chemical shifts are given in parts per million (ppm) with respect to tetramethylsilane (TMS, δ 0.00 ppm) as internal standard for ^{1}H NMR. Coupling constants are reported as J-values in Hz. Peak assignment is based on 2D gDQCOSY, ^{1}H-^{13}C gHSQCED, and ^{1}H-^{13}C gHMBC spectra. Side groups and end of chain signals separated from the bulk polymer ^{1}H signal are only reported when observed with clear s/n ratio and no overlap with polymer peaks, and may be (in)visible on other NMR spectrometers or with different concentrations. Gel permeation chromatography (GPC) equipped with PL gel 5 μm mixed D column calibrated for polystyrene (580 to 377,400 g/mol) was carried out on a Shimadzu instrument with THF as eluent using differential refractive index and UV (254 nm) detectors. Transmission electron microscopy (TEM) was carried out on a JEOL TEM 1400 equipped with CCD camera at 60 kV. Samples were prepared by drop casting 5 μL of appropriately diluted samples on a carbon coated Cu grid (200 mesh) and dried overnight at room temperature. Cryogenic TEM was carried out with a JEOL TEM 2100. Malvern Zetasizer nano S was used for dynamic light scattering (DLS) measurements equipped with He-Ne laser of wavelength 633 nm. Fluorescence was measured on a Tecan Spark 200. All images analysis was carried out using ImageJ, available in a public domain http://fiji.sc/ [3]. 300 W xenon light source was purchased from Asahi Spectra, Japan (MAX-303) with a wavelength range of 385-740 nm.

2. Synthesis of block co-polymers

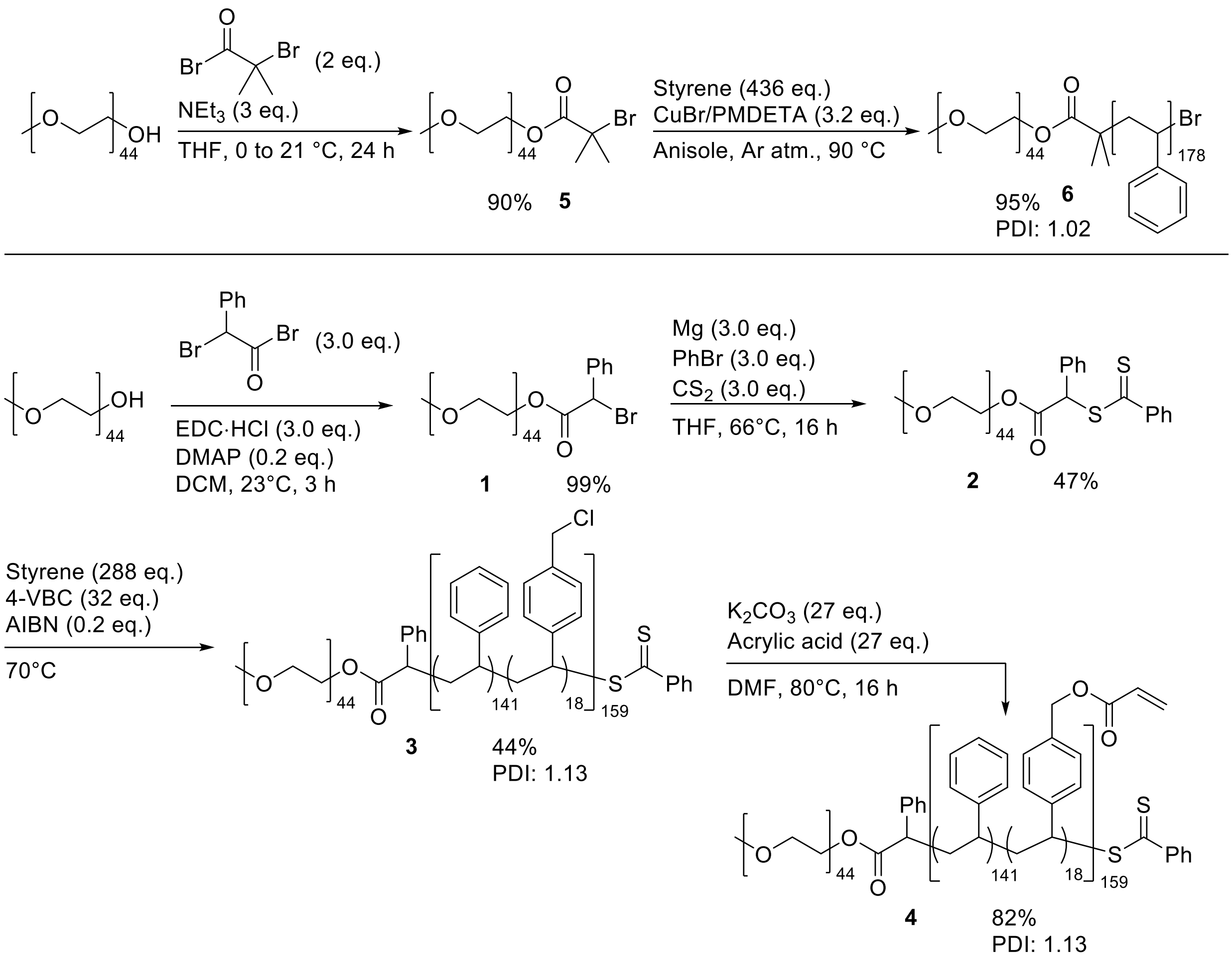

Scheme S1. Synthetic route for block copolymer poly(ethylene glycol)-*b*-polystyrene **6** and α-methoxy-poly(ethylene glycol)-*b*-poly(styrene-*co*-4-vinylbenzyl acrylate) **4**

2.1 α-methoxy-poly(ethylene glycol) ATRP macromolecular initiator (5)

α-methoxy-ω-hydroxy-poly(ethylene glycol) (5.0 g, 2.5 mmol, 1 eq.) was dried by co-evaporation with toluene to remove excess water. The polymer was dissolved in distilled THF (20 mL) followed by addition of trimethylamine (1.04 mL, 7.50 mmol, 3 eq.) in a flame-dried Schlenk flask and the mixture was cooled to 0 °C. α-bromoisobutyryl bromide (616 μL, 5.00 mmol, 2 eq.) was added dropwise and the mixture was stirred for 24 h, slowly warming to 21 °C. After the reaction, the mixture was filtered and subsequently concentrated under reduced pressure. The polymer was precipitated in ice-cold diethyl ether (3x) and dried *in vacuo* overnight to yield **5** as a white powder (4.84 g, 90%). **^{1}H NMR** (400 MHz, $CDCl_3$) δ 4.33 (m, 2H, $C\mathit{H_2}CH_2OC(O)$), 3.76 (m, 2H, $CH_2C\mathit{H_2}OC(O)$), 3.65 (br.s, 170H, PEG), 3.57−3.53 (m, 2H, $CH_3OC\mathit{H_2}$), 3.38 (s, 3H, $C\mathit{H_3}OCH_2$), 1.94 (s, 6H, $C(C\mathit{H_3})_2Br$) ppm. **^{13}C NMR** (101 MHz, $CDCl_3$) δ 171.6 ($O\mathit{C}(O)$), 71.9 ($CH_3O\mathit{C}H_2$), 70.5 (PEG), 68.7 ($\mathit{C}H_2CH_2OC(O)$, 65.1 ($CH_2\mathit{C}H_2OC(O)$), 59.0 ($\mathit{C}H_3OCH_2$), 55.7 ($Br\mathit{C}(CH_3)_2$), 30.8 ($BrC(\mathit{C}H_3)_2$).

2.2 α-methoxy-poly(ethylene glycol)-*b*-polystyrene (6)

A Schlenk tube was flame-dried under vacuum, charged with CuBr (90 mg, 0.64 mmol, 3.2 eq.) , and evacuated for 15 min and refilled with argon (3x). PMDETA (132 µL, 0.64 mmol, 3.2 eq.) in anisole (1.0 mL) was added, after which the mixture was stirred vigorously for 15 min, followed by addition of styrene (10 mL, 87.2 mmol, 436 eq.) in anisole (0.5 mL). The mixture was then degassed for 15 min with argon. The mixture was cooled to 0 °C and **5** was added (430 mg, 0.2 mmol, 1 eq.) in anisole (2 mL), followed by another 15 min of degassing. The tube was transferred to a pre-heated oil bath at 90 °C and the reaction was monitored with ^{1}H NMR. Upon attainment of the required molecular weight, the solution was diluted with DCM and extracted with aqueous EDTA (65 mM) (3x), until the blue color from Cu disappeared. The organic layer was collected and dried with $MgSO_4$, and concentrated under reduced pressure. The polymer was precipitated with ice cold methanol (3x) and dried *in vacuo* overnight to yield **6** as a white powder (4.04 g, 95%). **^{1}H NMR** (400 MHz, $CDCl_3$) δ 7.24−6.86 (m, PS arom. meta and para), 6.58−6.28 (m, PS arom. ortho), 3.64 (br s, 176H, PEG), 3.38 (s, 3H, OCH_3), 2.30−1.70 (m, PS backbone C*H*), 1.70−1.17 (m, PS backbone CH_2), 1.00−0.93 (m, 6H, *b*). **^{13}C NMR** (101 MHz, $CDCl_3$) δ 177.2 (O*C*(O)), 145.9 (PS arom. ipso), 128.0 (PS arom. meta), 127.7 (PS arom. ortho), 125.7 (PS arom. para), 70.6 (PEG), 59.0 (*C*H_3OCH_2), 47.1−41.6 (PS backbone *C*H_2), 41.7 (Br*C*(CH_3)$_2$), 40.4 (PS backbone *C*H). M_w/M_n 1.05.

2.3 α-methoxy-ω-2-bromo-2-phenylacetate-poly(ethylene glycol) (1)

α-methoxy-ω-hydroxy-poly(ethylene glycol) (8.0 g, 4.0 mmol, 1 eq.) was dissolved in toluene (20 mL) and dried by azeotropic distillation. The polymer was subsequently dissolved in DCM (75 mL), after which DMAP (98 mg, 0.80 mmol, 0.2 eq.), EDC·HCl (2.30 g, 12.0 mmol, 3 eq.) and α-bromophenylacetic acid (2.58 g, 12.0 mmol, 3 eq.) were added. The mixture was stirred for 3 h at 21 °C, after which water (75 mL) was added to quench the reaction. The organic layer was then washed with saturated aqueous $NaHCO_3$ and saturated aqueous NH_4Cl solution, respectively. The organic layer was collected and dried with $MgSO_4$, and concentrated under reduced pressure. The polymer was precipitated in ice-cold diethyl ether (2x) and dried *in vacuo* overnight to yield **1** as a white powder (8.70 g, 99%). **^{1}H NMR** (500 MHz, $CDCl_3$) δ 7.58−7.48 (m, 2H, Ph ortho), 7.41−7.31 (m, 3H, Ph meta and para), 5.39 (s, 1H, C*H*-Ph), 4.40−4.25 (m, 2H, C$H_2$$CH_2$OC(O)), 3.72−3.68 (m, 2H, CH_2CH_2OC(O)), 3.64 (br.s, 170H, PEG), 3.57−3.53 (m, 2H, CH_3OCH_2), 3.38 (s, 3H, CH_3OCH_2). **^{13}C NMR** (125 MHz, $CDCl_3$) δ 168.2 (O*C*(O)), 129.3 (Ph ipso), 128.8 (Ph meta and para), 128.7 (Ph ortho), 128.0 (Ph ortho), 71.9 (CH_3O*C*H_2), 70.6 (PEG), 68.7 (CH_2*C*H_2OC(O)), 65.5 (*C*$H_2$$CH_2$OC(O)), 59.0 (*C*$H_3OCH_2$), 46.5 (*C*H-Ph). ***R*$_f$** 0.36 (MeOH/DCM, 1:9 v/v).

2.4 α-methoxy-ω-2-phenyl-2-(phenylcarbonothioyl)thio)acetate-poly(ethylene glycol) (2)

A Schlenk tube was flame-dried under vacuum, and loaded with magnesium turnings (292 mg, 12.0 mmol, 3 eq.), and evacuated for 15 min and refilled with argon (3x). Afterwards, dry THF (30 mL) and an I_2 crystal were added. A solution of bromobenzene (1.88 g, 12.0 mmol, 3 eq.) in dry THF (30 mL) was added dropwise and the mixture was stirred at 50 ˚C for 1 h. Carbon disulfide (914 mg, 12.0 mmol, 3 eq.) was added, after which the mixture was stirred for another 30 min at 50 ˚C. A solution of **1** (8.7 g, 4.0 mmol, 1 eq.) in dry THF (20 mL) was added and the dark red solution was refluxed for 16 h. The reaction mixture was filtered to remove the left-over magnesium and concentrated under reduced pressure, and subsequently purified by column chromatography on silica gel using MeOH/DCM (gradient to 1:9 v/v) as eluent. The polymer was precipitated in ice-cold diethyl ether (2x) and dried *in vacuo* overnight to yield **2** as a pink powder (4.3 g, 47%) as a pink solid. **^{1}H NMR** (400 MHz, $CDCl_3$) δ 8.03–7.98 (m, 2H, CTA Ph ortho), 7.57−7.51 (m, 1H, CTA Ph para), 7.51−7.46 (m, 2H, Ph ortho), 7.41−7.31 (m, 5H, Ph meta and para & CTA Ph meta), 5.73 (s, 1H, C*H*-Ph), 4.42–4.23 (m, 2H, C$H_2$$CH_2$OC(O)), 3.71−3.67 (m, 2H, CH_2CH_2OC(O)), 3.64 (br.s, 170H, PEG), 3.58−3.52 (m, 2H, CH_3OCH_2), 3.38 (s, 3H, CH_3OCH_2). **^{13}C NMR** (101 MHz, $CDCl_3$) δ 225.9 (S*C*(S)Ph), 168.8 (O*C*(O)), 143.9 (CTA Ph ipso), 133.2 (Ph ipso), 132.8 (CTA Ph para), 129.0 (Ph meta), 128.9 (Ph para and ortho), 128.4 (CTA Ph meta), 126.9 (CTA Ph ortho), 71.9 (CH_3O*C*H_2), 70.6 (PEG), 68.8 (CH_2*C*H_2OC(O)), 65.3 (*C*$H_2$$CH_2$OC(O)), 59.0 (*C*$H_3OCH_2$), 58.8 (*C*H-Ph). $\boldsymbol{R_f}$ 0.33 (MeOH/DCM, 1:9 v/v).

2.5 α-methoxy-poly(ethylene glycol)-*b*-poly(styrene-*co*-4-vinylbenzyl chloride) (PEG$_{44}$-*b*-P(S$_{138}$-*co*-4-VBC$_{18}$)) (3)

A flame-dried Schlenk tube equipped with a stirring bar was loaded with styrene (1.7 mL, 14 mmol, 288 eq.), purified 4-vinylbenzyl chloride (326 mg, 1.92 mmol, 32 eq.), **2** (100 mg, 0.05 mmol, 1 eq.) and AIBN (1.7 mg, 0.010 mmol, 0.2 eq.). Anisole (0.18 mL, 1.6 mmol, 32 eq.) was added as internal standard. The mixture was then degassed for 15 min with argon. The Schlenk tube was then immersed in a preheated oil bath of 70 °C and the polymerization was monitored by ^{1}H NMR spectroscopy. When the required length was obtained, the polymerization was terminated by removing the Schlenk tube from the oil bath and diluting the mixture with DCM. The mixture was then concentrated under reduced pressure. The polymer was precipitated with ice cold methanol (3x) and dried *in vacuo* overnight to yield **3** as a pink solid (0.88 g, 44%). **^{1}H NMR** (400 MHz, $CDCl_3$) δ 7.22−6.85 (m, PS arom. meta and para), 6.82−6.19 (m, PS arom. ortho), 4.60–4.40 (br s, Ph-CH_2-Cl), 3.64 (br s, 176H, PEG), 3.38 (s, 3H, CH_3OCH_2), 2.36−1.64 (m, PS backbone C*H*), 1.61−1.07 (m, PS backbone CH_2). **^{13}C NMR** (101 MHz, $CDCl_3$) δ 145.9 (PS arom. ipso), 128.0 (PS arom. meta), 127.7 (PS arom.

ortho), 125.7 (PS arom. para), 70.6 (PEG), 59.0 (CH_3OCH_2), 54.6 (Ph-CH_2-Cl), 43.2 (PS backbone CH_2), 40.5 (PS backbone *C*H). M_w/M_n 1.13.

2.6 α-methoxy-poly(ethylene glycol)-*b*-poly(styrene-*co*-4-vinylbenzyl acrylate) (PEG_{44}-*b*-P(S_{138}-*co*-4-VBA_{18})) (4)

A flame-dried Schlenk tube equipped with a stirring bar was loaded with K_2CO_3 (150 mg, 1.1 mmol, 27 eq.) in DMF (4 mL) and cooled to 0 °C. Acrylic acid (76 µL, 1.1 mmol, 27 eq.) was added and the mixture was stirred for 1 h at 0 °C. Then, **3** (0.80 g, 40 µmol, 1 eq.) was added and the mixture was stirred for 3 h at 80 °C. Afterwards, the reaction mixture was diluted with DCM and extracted with water (2x) and brine (2x), concentrated under reduced pressure and precipitated in ice cold methanol (3x). If required, the polymer can subsequently purified by column chromatography on silica gel using MeOH/DCM (5:95 v/v) as eluent. The polymer was filtered and dried overnight *in vacuo* to yield **4** as a pink powder (0.66 g, 82%). **^{1}H NMR** (400 MHz, $CDCl_3$) δ 7.23−6.84 (m, PS arom. meta and para), 6.82−6.26 (m, PS arom. ortho and acrylate C*H*CH_2), 6.23–6.08 (m, acrylate CHCH_2), 5.89–5.74 (m, acrylate CHCH_2), 5.21–5.01 (br s, Ph-CH_2-acrylate), 3.64 (br s, 176H, PEG), 3.38 (s, 3H, CH_3OCH_2), 2.36−1.64 (m, PS backbone C*H*), 1.61−1.07 (m, PS backbone CH_2). **^{13}C NMR** (101 MHz, $CDCl_3$) δ 145.3 (PS arom. ipso), 128.0 (PS arom. meta), 127.7 (PS arom. ortho), 125.6 (PS arom. para), 70.6 (PEG), 59.0 (CH_3OCH_2), 66.1 (Ph-CH_2-acrylate), 43.2 (PS backbone CH_2), 40.5 (PS backbone *C*H). M_w/M_n 1.13.

2.7 Synthesis of probe. Prop-2-yn-1-yl (4-methyl-2-oxo-2H-chromen-7-yl)carbamate (Propargyl-carbamate masked coumarin) (7). 7-Amino-4-methylcoumarin (99.2 mg, 0.567 mmol, 1 eq.) and pyridine (52.4 µL, 0.65 mmol, 1.15 eq.) were suspended in DCM (3 mL), after which propargyl chloroformate (68.1 µL, 0.70 mmol, 1.23 eq.) was added to the dark yellow suspension. The mixture was stirred at 0 °C for 16 h, after which it turned bright yellow. Then 0.5 M HCl (40 mL) was added, and the mixture was subsequently filtered and washed with diethyl ether (2x). The resulting solid was dried *in vacuo* overnight to yield **7** as a yellow powder (119 mg, 82%). **^{1}H NMR** (500 MHz, $CDCl_3$) δ 7.56 (d, *J*=8.6 Hz, 1H, H9), 7.46 (d, *J*=2.2 Hz, 1H, H6), 7.39 (dd, *J*=8.7, 2.2 Hz, 1H, H8), 6.93 (s, 1H, N*H*), 6.23 (q, *J*=1.3 Hz, 1H, H13), 4.82 (d, *J*=2.5 Hz, 2H, H3), 2.56 (t, *J*=2.4 Hz, 1H, H1), 2.49 (d, *J*=1.3 Hz, 3H, H12). **^{13}C NMR** (125 MHz, $CDCl_3$) δ 160.9 (C14), 159.2 (C7), 152.0 (C11), 151.9 (C4), 140.9 (C5), 125.4 (C9), 115.9 (C10), 114.4 (C8), 113.5 (C13), 106.1 (C6), 77.2 (C2), 75.4 (C1), 52.9 (C3), 18.6 (C12). ***R*$_f$** 0.32 (MeOH/DCM, 1:9 v/v).

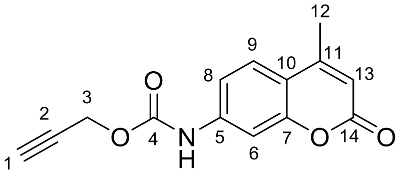

2.8 Self-assembly procedures

2.8.1 General synthesis of polymersomes

PEG-*b*-PS polymer **6** (10 mg) was dissolved in a mixture of THF and 1,4-dioxane (1 mL, 4:1 v/v) in a 15 mL vial with a magnetic stir bar. After dissolving the polymer for 0.5 h at 21 °C, a syringe pump equipped with a syringe and a needle was used to deliver ultrapure water with a rate of 1 mL/h for 0.5 h via a rubber septum, while vigorously stirring the mixture (900 rpm). Upon finishing the water addition, 8.0 mL of ultrapure water was added to the suspension to quench the polymersomes. The polymersomes were spun down using a centrifuge (10 min, 13.000 rpm) and washed with ultrapure water a total of three times.

2.8.2 General synthesis of cross-linked polymersomes

Cross-linkable polymer **4** (10 mg) was dissolved in a mixture of THF and 1,4-dioxane (1 mL, 4:1 v/v) in a 15 mL aluminum foil wrapped vial with a magnetic stir bar. 0.5 equivalents of Irgacure 2959 were added from a stock solution. After dissolving the polymer for 0.5 h at 21 °C, a syringe pump equipped with a syringe and a needle was used to deliver ultrapure water with a rate of 1 mL/h for 0.5 h via a rubber septum, while vigorously stirring the mixture (900 rpm). Upon finishing the water addition, the polymersome mixture was degassed by flushing the solution with argon for 5 minutes, after which the vial is immediately placed under a UV-lamp to start the crosslinking process. The polymersome suspension was irradiated for 5 minutes at 60% power with the full wavelength range. 8.0 mL of ultrapure water was then added to quench the polymersomes. The polymersomes were spun down using a centrifuge (10 min, 13.000 rpm) and washed with ultrapure water a total of three times.

2.8.3 General procedure of resuspending cross-linked polymersomes in organic solvent

The cross-linked polymersomes in ultrapure water were spun down using a centrifuge (10 min, 13.000 rpm) after which the supernatant was removed. The pellet was then resuspended in the organic solvent, and washed with organic solvent a total of three times.

2.8.4 Synthesis of cross-linked polymersomes with functional handles.

Similar procedure as **2.8.2**, substituting cross-linkable polymer **4** (10 mg) for cross-linkable polymer **4** (9 mg) and handle-PEG-*b*-PS (1 mg). [4]

2.8.5 Click reaction of DBCO-handle and 3-azido-7-hydroxycoumarin

2 x 300 µL of THF suspended cross-linked polymersomes with 10% DBCO-handle were added to two Eppendorf tubes. To one of these tubes, 0.50 mg of 3-azido-7-hydroxycoumarin was added. The polymersomes in the other tube were washed with THF three times and washed back into water. After this, 0.50 mg of 3-azido-7-hydroxycoumarin was added to the second tube. Both tubes were shaken using an Eppendorf thermomixer for 1 hour. The fluorescence was measured using an excitation wavelength of 485 nm (bandwidth 20 nm) and a detection wavelength of 535 nm (bandwidth 20 nm). [4]

2.8.6 Synthesis of cross-linked polymersomes with pores

Similar procedure as **2.8.2**, substituting cross-linkable polymer **4** (10 mg) for cross-linkable polymer **4** (10-x mg) and PEG-*b*-PS (x mg). The number x ranges from 1 to 8, forming the 90%-20% cross-linked polymersomes.

2.8.7 Synthesis of cross-linked polymersomes with Pt NP

Similar procedure as **2.8.2** or **2.8.6**, with the modification of adding a dispersion of Pt NPs in ultrapure water instead of only ultrapure water. To remove the excess PtNPs, the polymersomes were washed three times using a 0.22 µm centrifugal filter (10 min, 13.000 rpm) after the standard washing procedure.

3. TEM images of Irgacure 2959 Optimisation

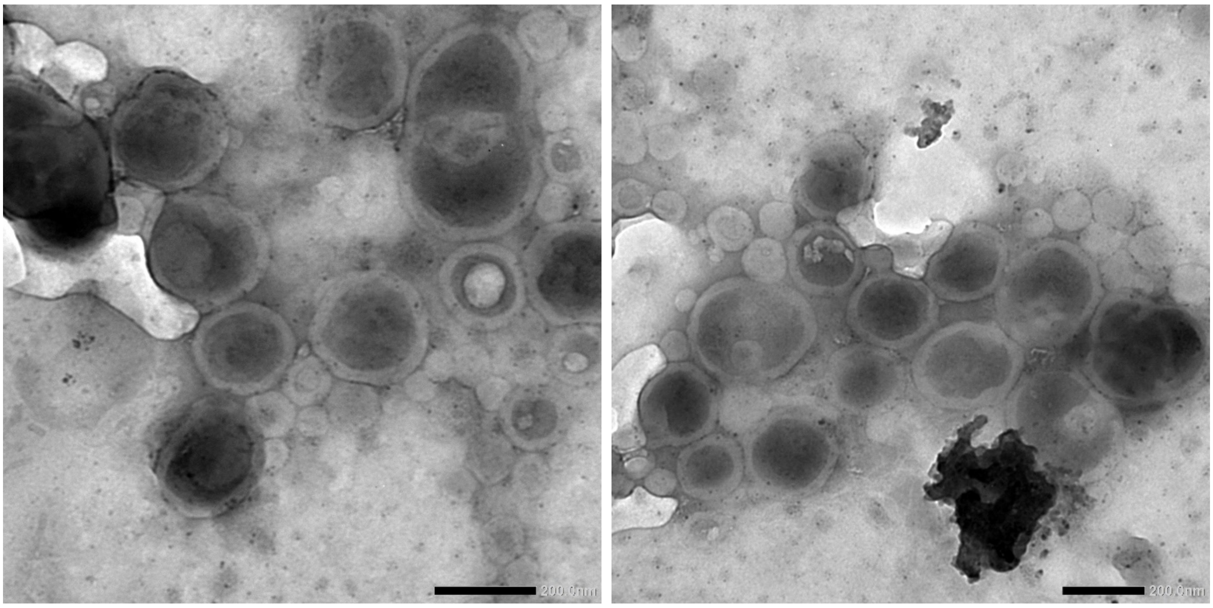

Figure S1: TEM images from THF of MeO-PEG$_{44}$-*b*-P(S$_{141}$-*co*-4-VBA$_{18}$)$_{159}$ polymersomes cross-linked using 0.011 eq. Irgacure 2959 compared to the amount of 4-VBA incorporated in the polymer. Cross-linking was not sufficient to create stable polymersomes. Scale bar: 200 nm

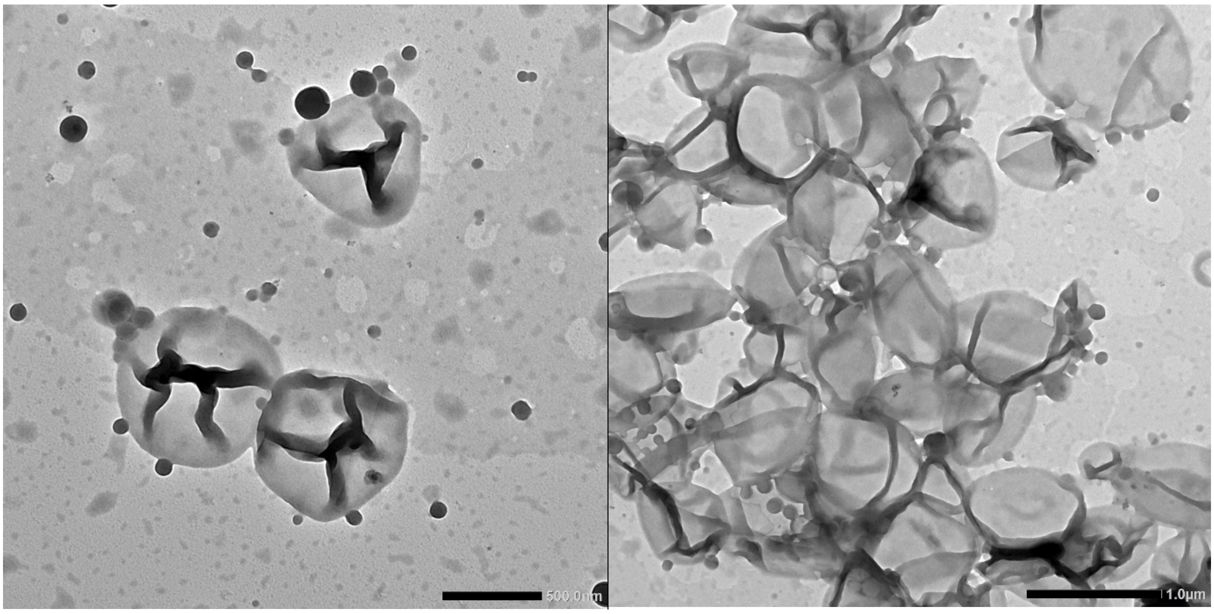

Figure S2: TEM images from THF of MeO-PEG$_{44}$-*b*-P(S$_{141}$-*co*-4-VBA$_{18}$)$_{159}$ polymersomes cross-linked using 0.111 eq. Irgacure 2959 compared to the amount of 4-VBA incorporated in the polymer. Cross-linking was sufficient to create stable polymersomes. Scale bar: 500 nm (left), 1000 nm (right)

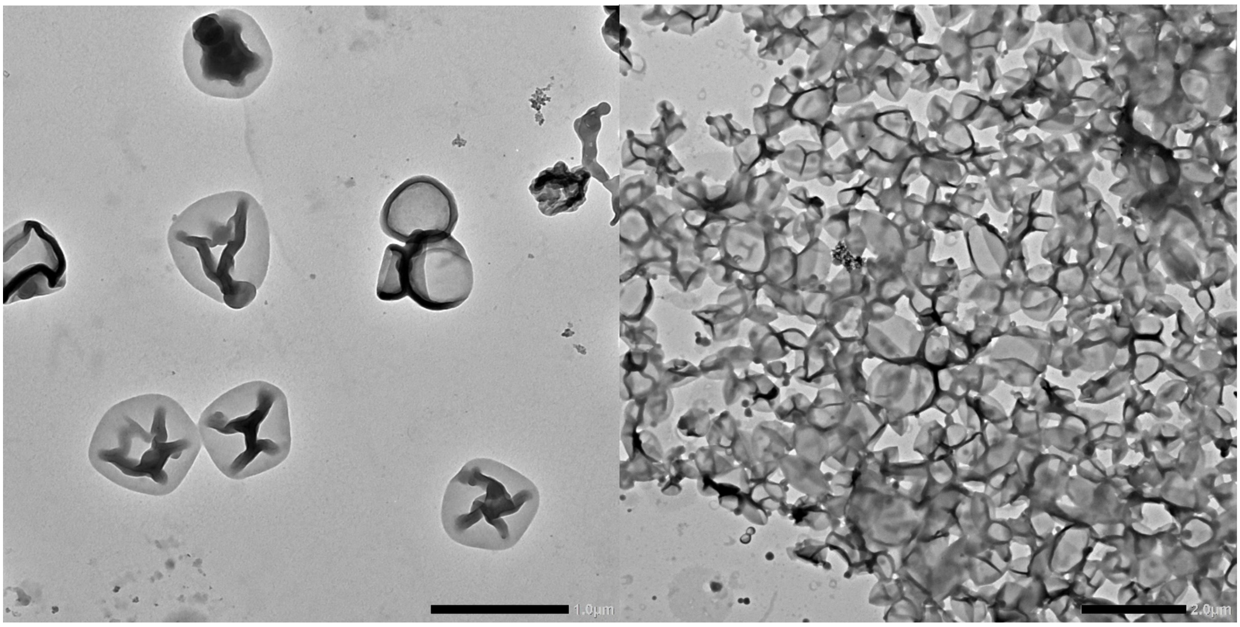

Figure S3: TEM images from THF of MeO-PEG$_{44}$-*b*-P(S$_{141}$-*co*-4-VBA$_{18}$)$_{159}$ polymersomes cross-linked using 1.111 eq. Irgacure 2959 compared to the amount of 4-VBA incorporated in the polymer. Cross-linking was sufficient to create stable polymersomes. Scale bar: 1000 nm (left), 2000 nm (right)

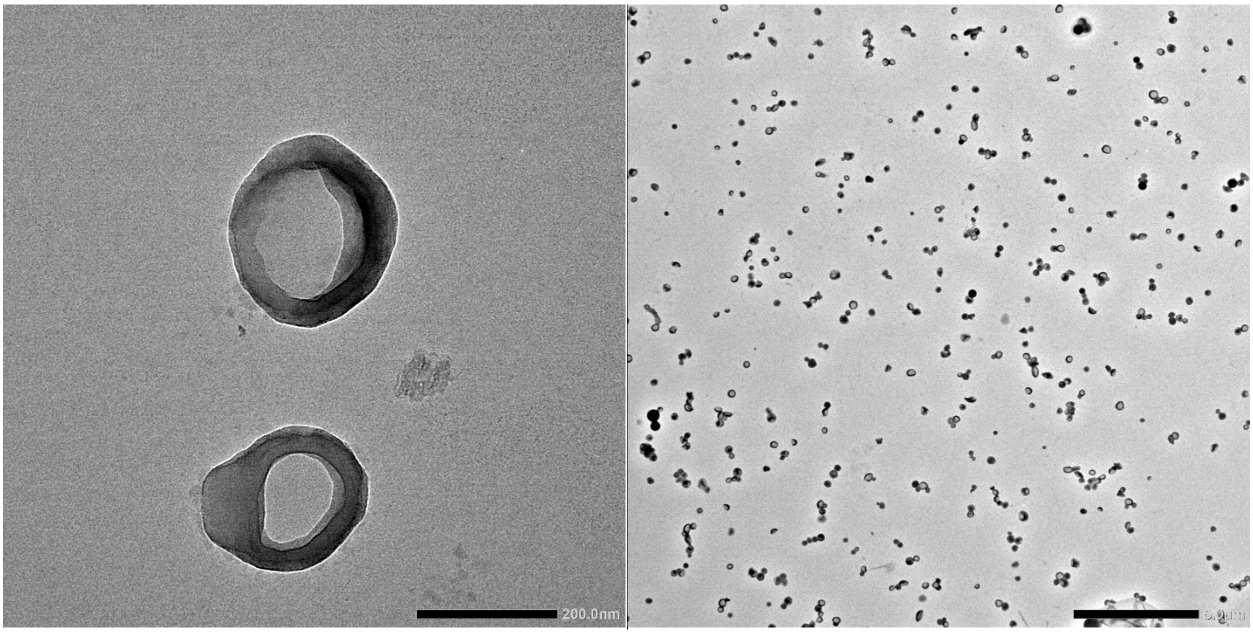

Figure S4: TEM images from water of MeO-PEG$_{44}$-*b*-P(S$_{141}$-*co*-4-VBA$_{18}$)$_{159}$ polymersomes cross-linked using 0.014 eq. Irgacure 2959 compared to the amount of 4-VBA incorporated in the polymer. Scale bar: 200 nm (left), 5000 nm (right)

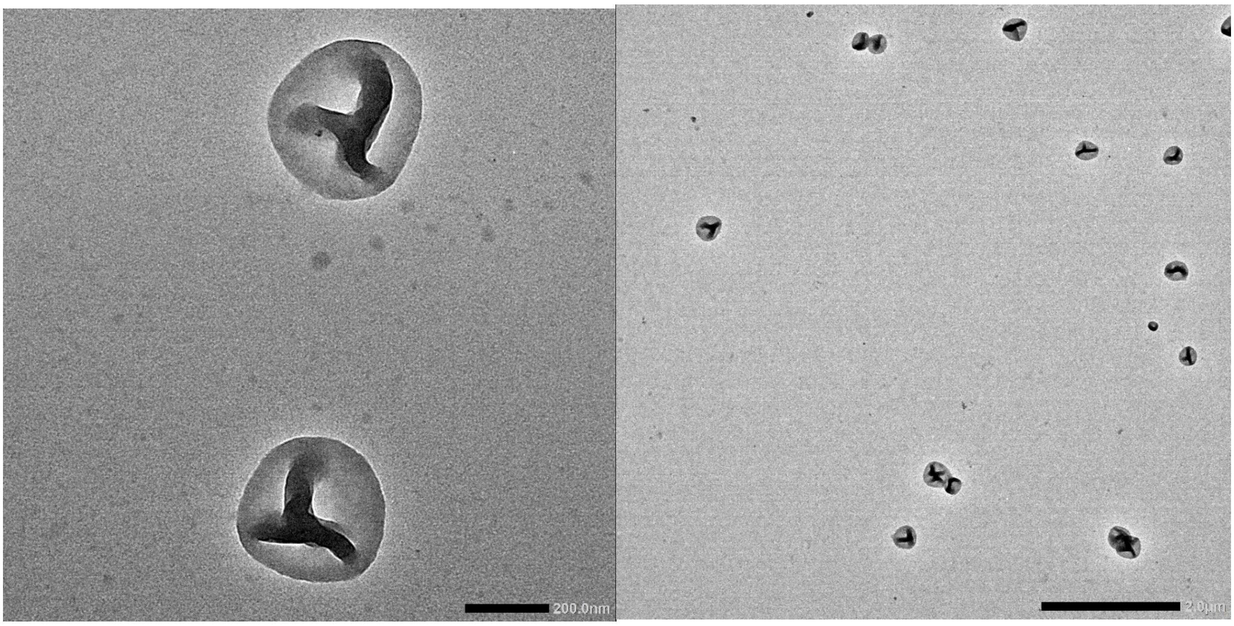

Figure S5: TEM images from THF of MeO-PEG_{44}-*b*-P(S_{141}-*co*-4-VBA_{18})$_{159}$ polymersomes cross-linked using 0.014 eq. Irgacure 2959 compared to the amount of 4-VBA incorporated in the polymer. Cross-linking was sufficient to create stable small polymersomes. Scale bar: 200 nm (left), 2000 nm (right)

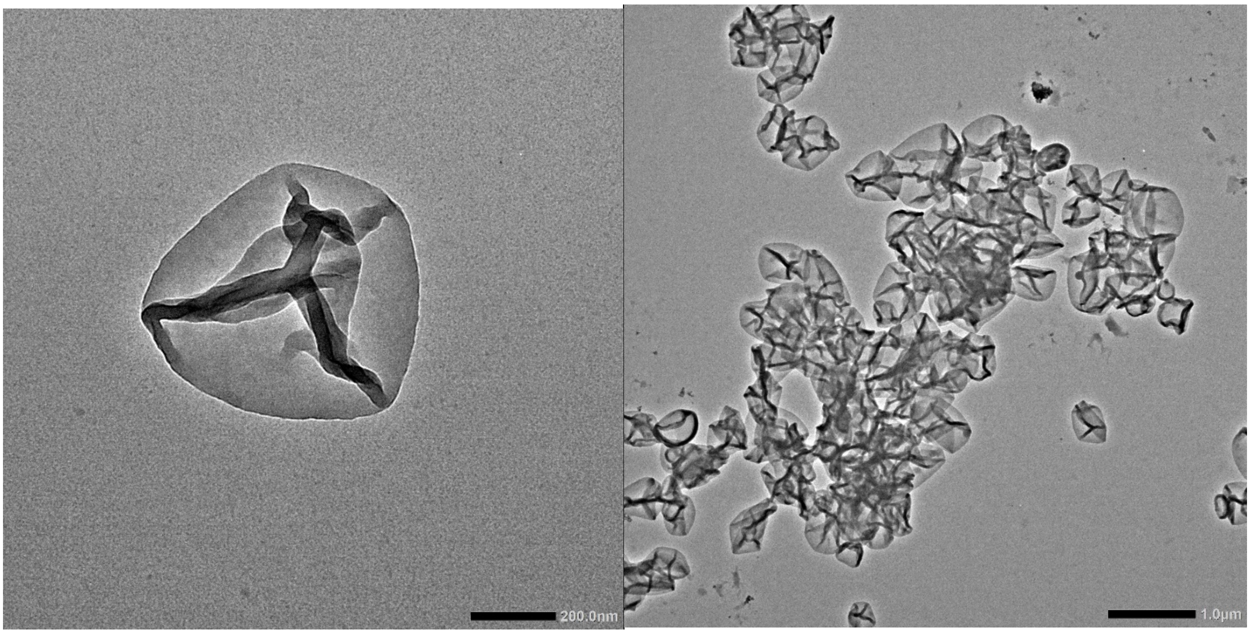

Figure S6: TEM images from water of MeO-PEG_{44}-*b*-P(S_{141}-*co*-4-VBA_{18})$_{159}$ polymersomes cross-linked using 0.028 eq. Irgacure 2959 compared to the amount of 4-VBA incorporated in the polymer. Scale bar: 200 nm (left), 1000 nm (right)

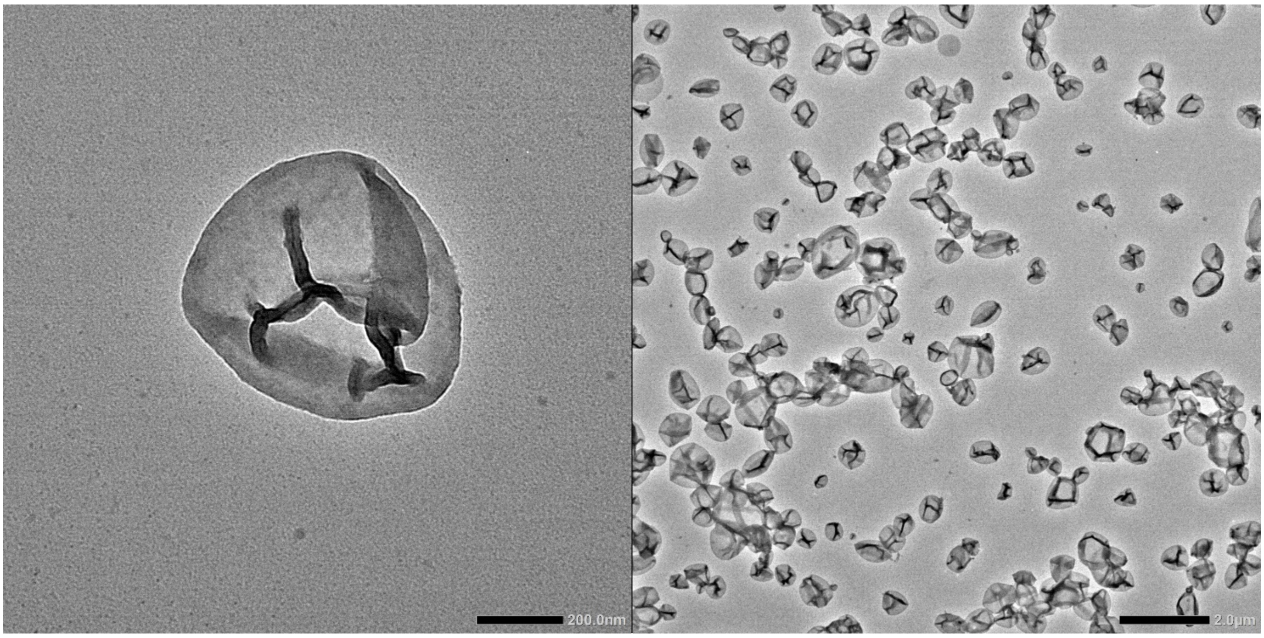

Figure S7: TEM images from THF of MeO-PEG$_{44}$-*b*-P(S$_{141}$-*co*-4-VBA$_{18}$)$_{159}$ polymersomes cross-linked using 0.028 eq. Irgacure 2959 compared to the amount of 4-VBA incorporated in the polymer. Cross-linking was sufficient to create stable polymersomes. Scale bar: 200 nm (left), 2000 nm (right)

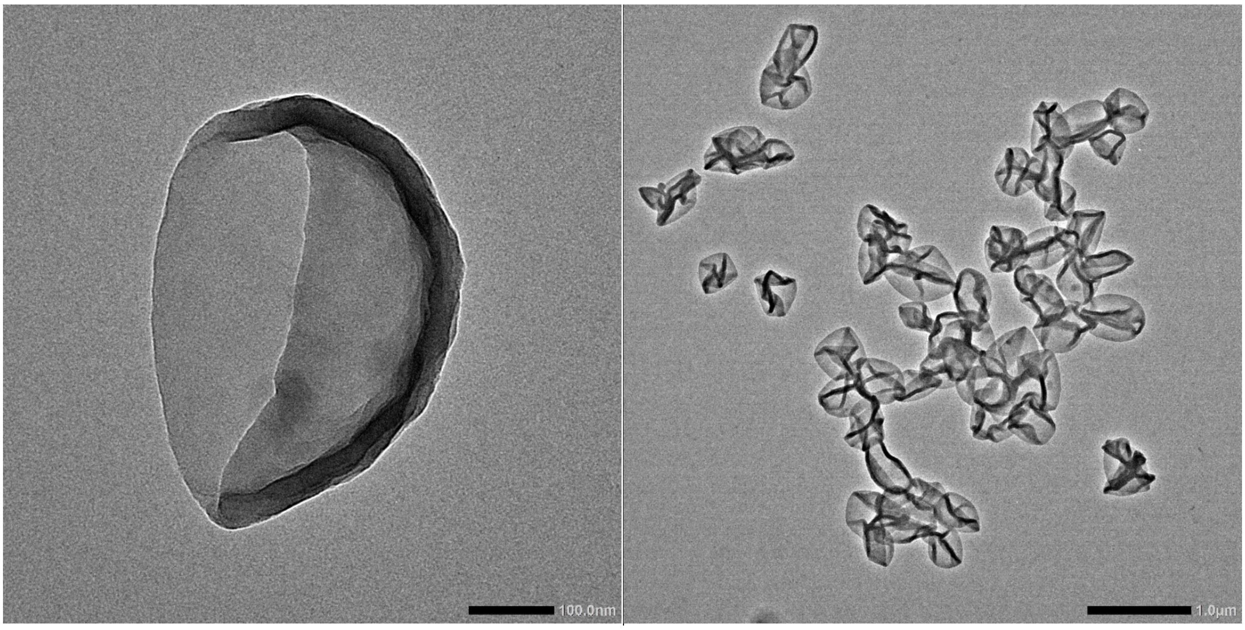

Figure S8: TEM images from water of MeO-PEG$_{44}$-*b*-P(S$_{141}$-*co*-4-VBA$_{18}$)$_{159}$ polymersomes cross-linked using 0.056 eq. Irgacure 2959 compared to the amount of 4-VBA incorporated in the polymer. Scale bar: 100 nm (left), 1000 nm (right)

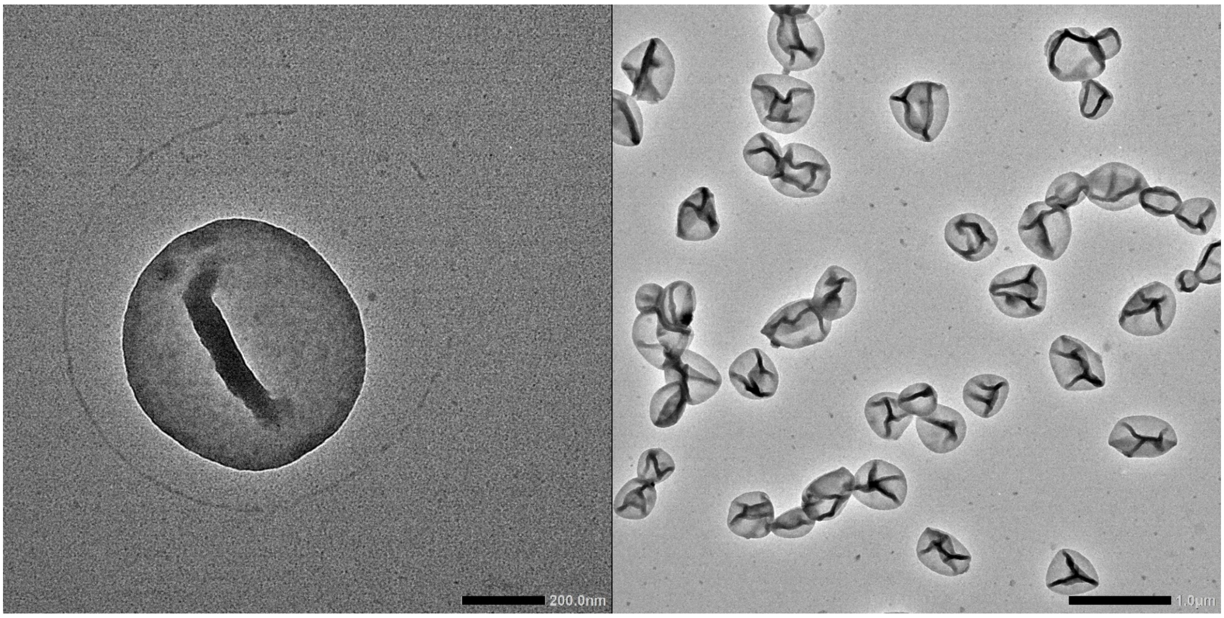

Figure S9: TEM images from THF of MeO-PEG$_{44}$-*b*-P(S$_{141}$-*co*-4-VBA$_{18}$)$_{159}$ polymersomes cross-linked using 0.056 eq. Irgacure 2959 compared to the amount of 4-VBA incorporated in the polymer. Cross-linking was sufficient to create stable polymersomes. Scale bar: 200 nm (left), 1000 nm (right)

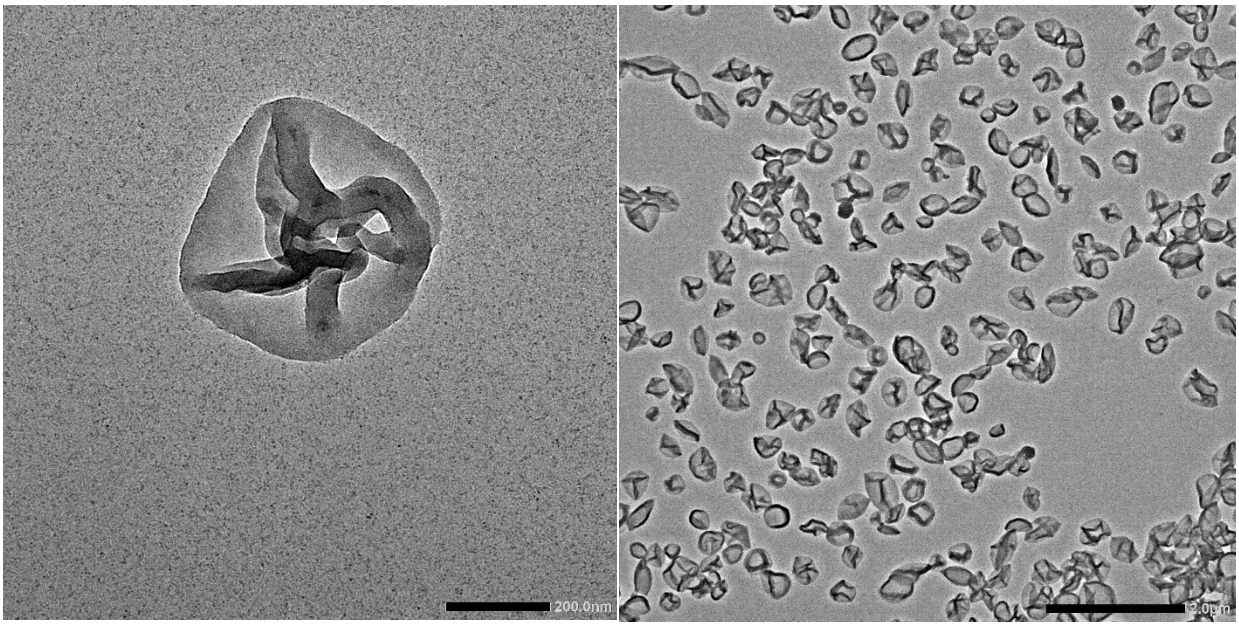

Figure S10: TEM images from water of MeO-PEG$_{44}$-*b*-P(S$_{141}$-*co*-4-VBA$_{18}$)$_{159}$ polymersomes cross-linked using 0.083 eq. Irgacure 2959 compared to the amount of 4-VBA incorporated in the polymer. Scale bar: 200 nm (left), 2000 nm (right)

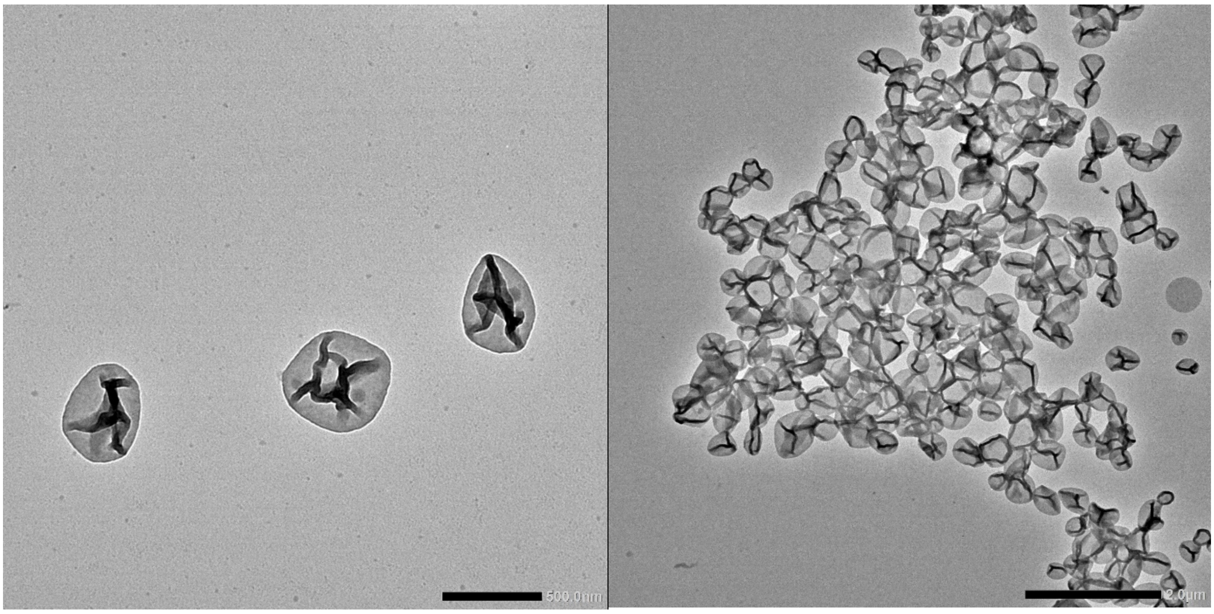

Figure S11: TEM images from THF of MeO-PEG$_{44}$-*b*-P(S$_{141}$-*co*-4-VBA$_{18}$)$_{159}$ polymersomes cross-linked using 0.083 eq. Irgacure 2959 compared to the amount of 4-VBA incorporated in the polymer. Cross-linking was sufficient to create stable polymersomes. Scale bar: 500 nm (left), 2000 nm (right)

4. Cryo-TEM image of Polymersomes under Various Angles

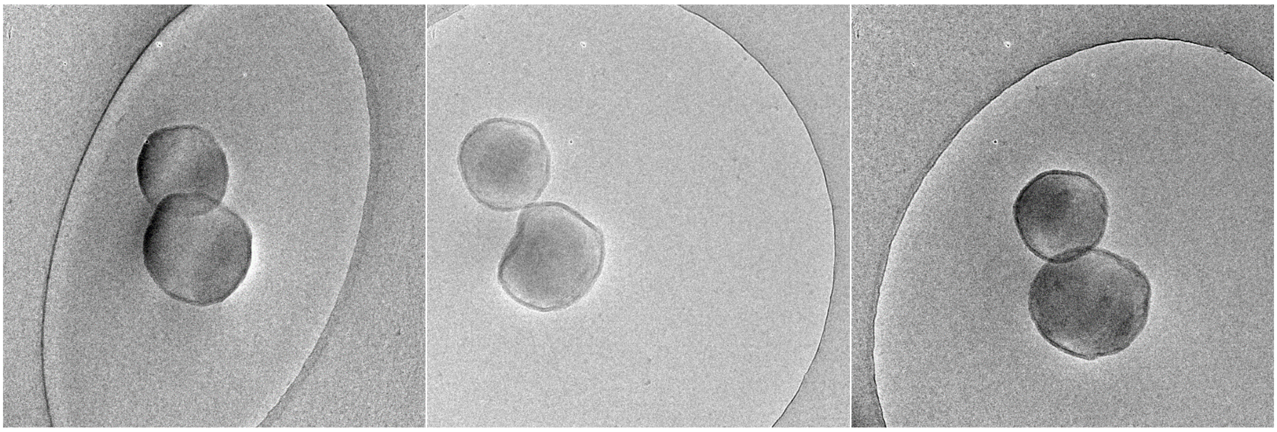

Figure S12: Cryo-TEM images from water of MeO-PEG$_{44}$-*b*-P(S$_{141}$-*co*-4-VBA$_{18}$)$_{159}$ polymersomes taken from various angles. Left: -45 degrees. Middle: 0 degrees. Right: +45 degrees.

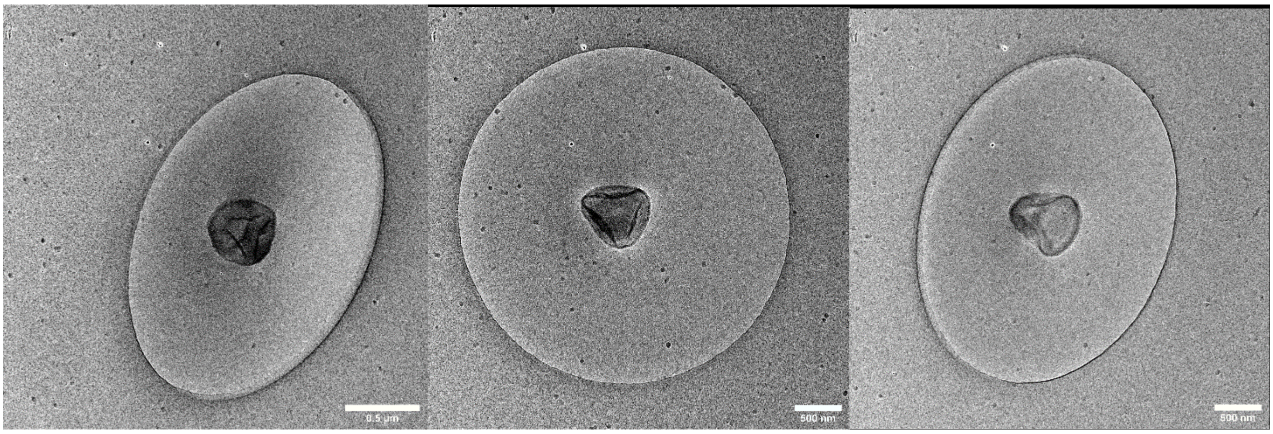

Figure S13: Cryo-TEM images from THF to water of MeO-PEG$_{44}$-*b*-P(S$_{141}$-*co*-4-VBA$_{18}$)$_{159}$ polymersomes taken from various angles. Left: -45 degrees. Middle: 0 degrees. Right: +45 degrees.

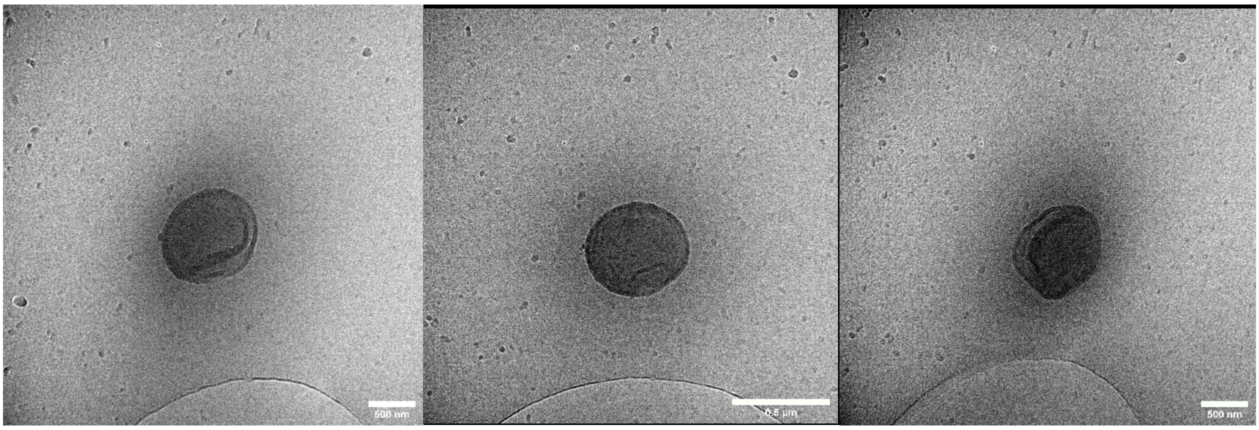

Figure S14: Cryo-TEM images from DCM to water of MeO-PEG$_{44}$-*b*-P(S$_{141}$-*co*-4-VBA$_{18}$)$_{159}$ polymersomes taken from various angles. Left: -45 degrees. Middle: 0 degrees. Right: +45 degrees.

5. Cryo-SEM image of Cross-Linked Polymersomes

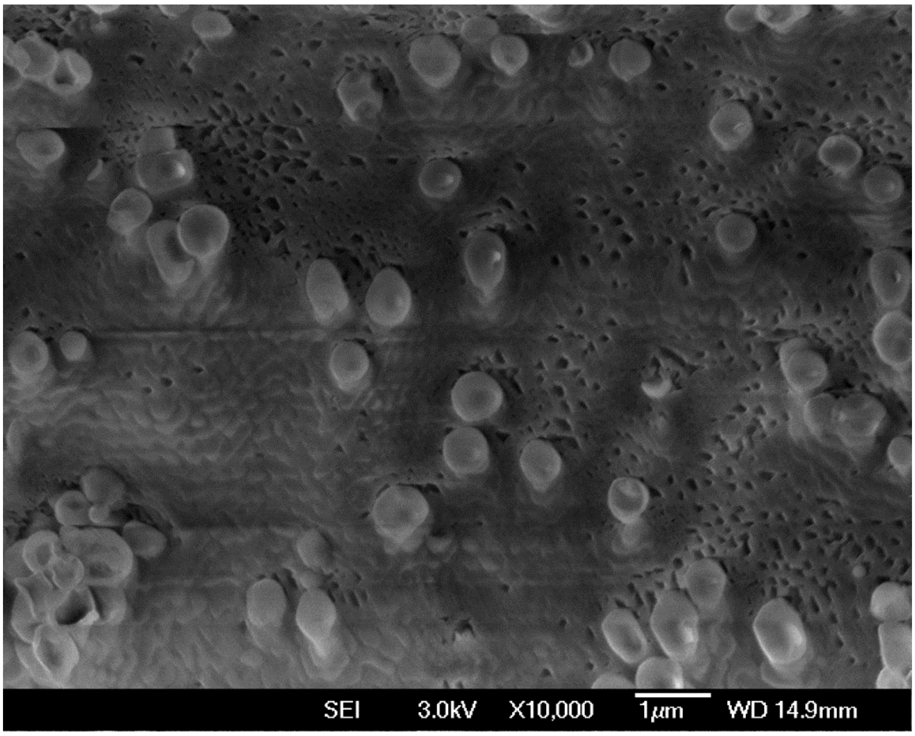

Figure S15: Cryo-SEM image of spherical MeO-PEG-*b*-P(S_{141}-*co*-4-VBA_{18})$_{159}$ polymersomes. Full version of Figure 3F. Scale bar: 1000 nm

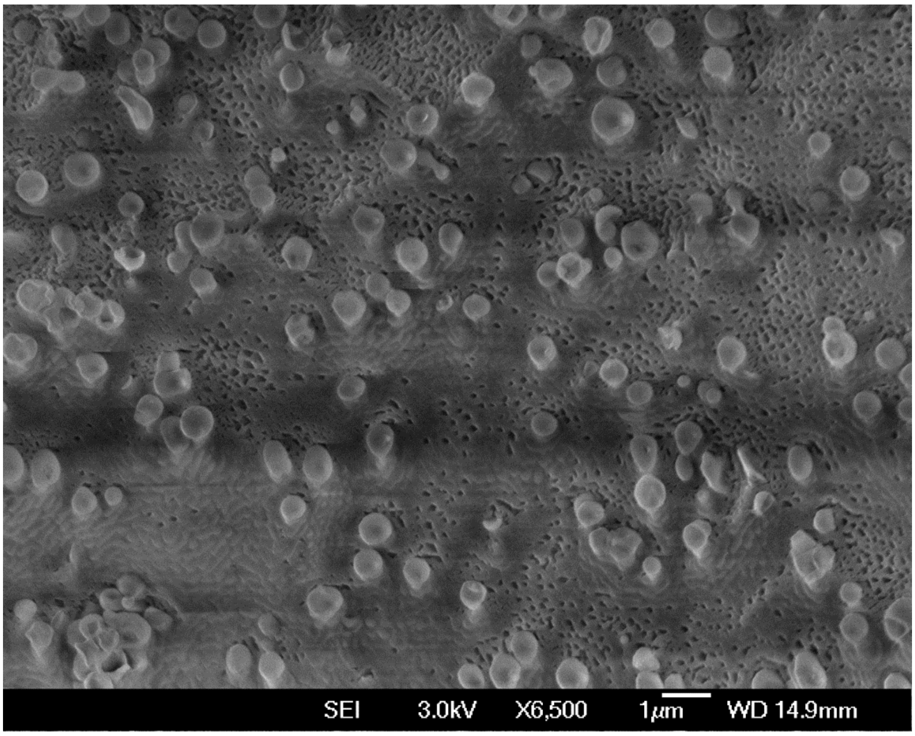

Figure S16: Cryo-SEM image of spherical MeO-PEG-*b*-P(S_{141}-*co*-4-VBA_{18})$_{159}$ polymersomes. Scale bar: 1000 nm

6. TEM images of Polymersome Solvent Stability Tests

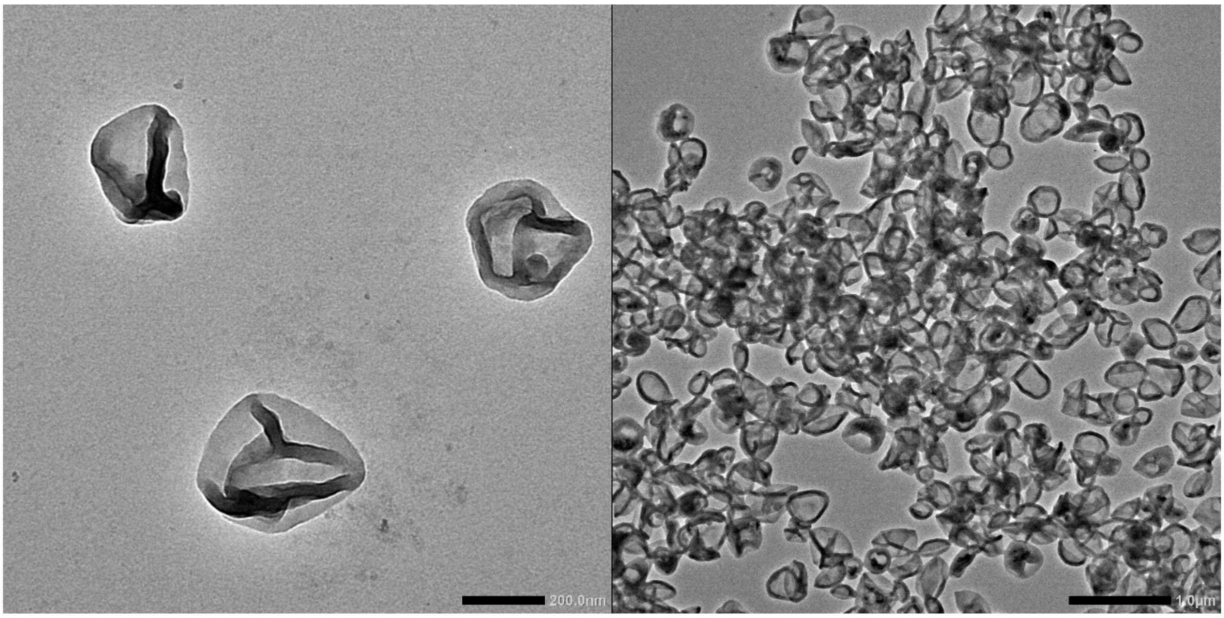

Figure S17: TEM images from water of MeO-PEG$_{44}$-*b*-P(S$_{141}$-*co*-4-VBA$_{18}$)$_{159}$ cross-linked polymersomes after 1 hour. Scale bar: 200 nm (left), 1000 nm (right)

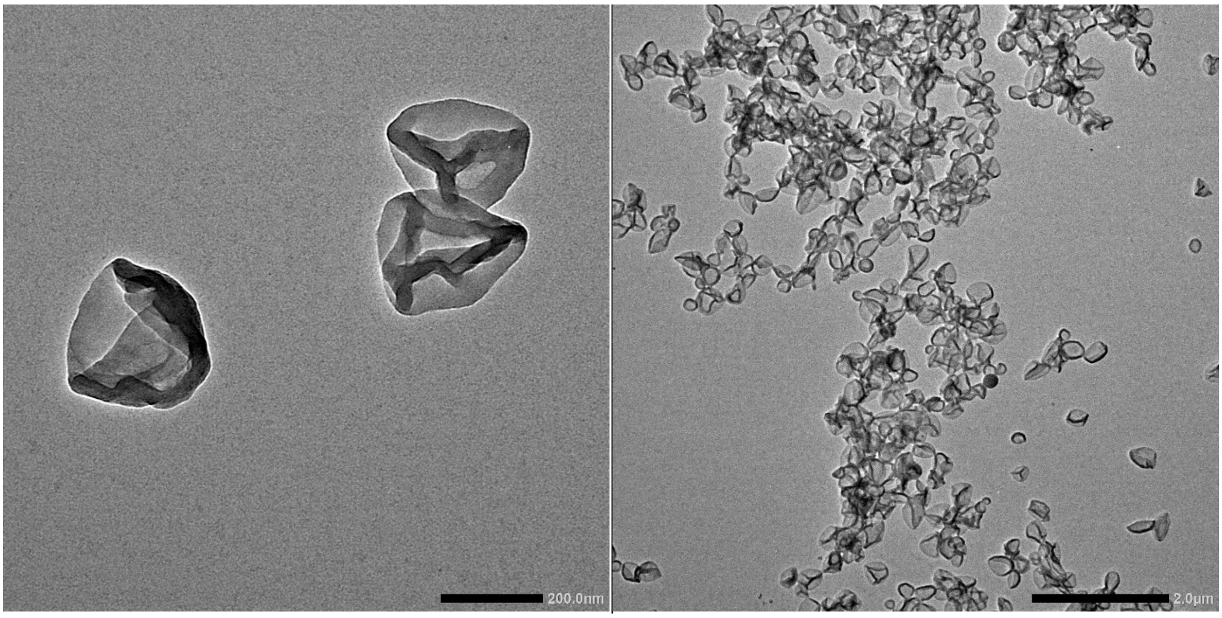

Figure S18: TEM images from water of MeO-PEG$_{44}$-*b*-P(S$_{141}$-*co*-4-VBA$_{18}$)$_{159}$ cross-linked polymersomes after 1 day. Scale bar: 200 nm (left), 2000 nm (right)

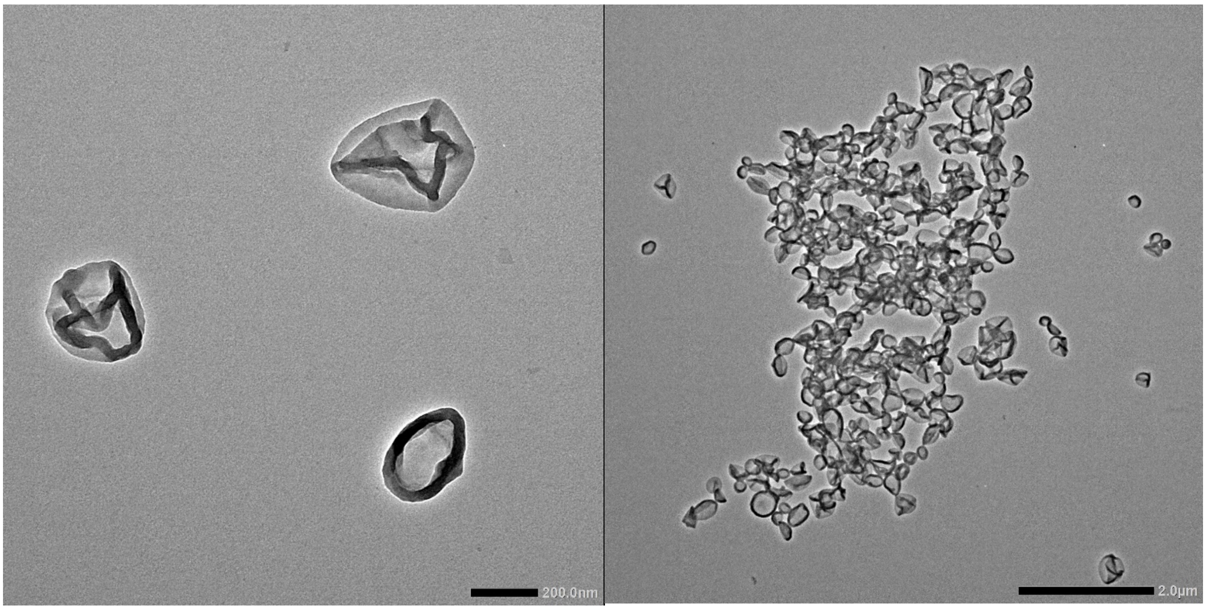

Figure S19: TEM images from water of MeO-PEG$_{44}$-*b*-P(S$_{141}$-*co*-4-VBA$_{18}$)$_{159}$ cross-linked polymersomes after 1 week. Scale bar: 200 nm (left), 2000 nm (right)

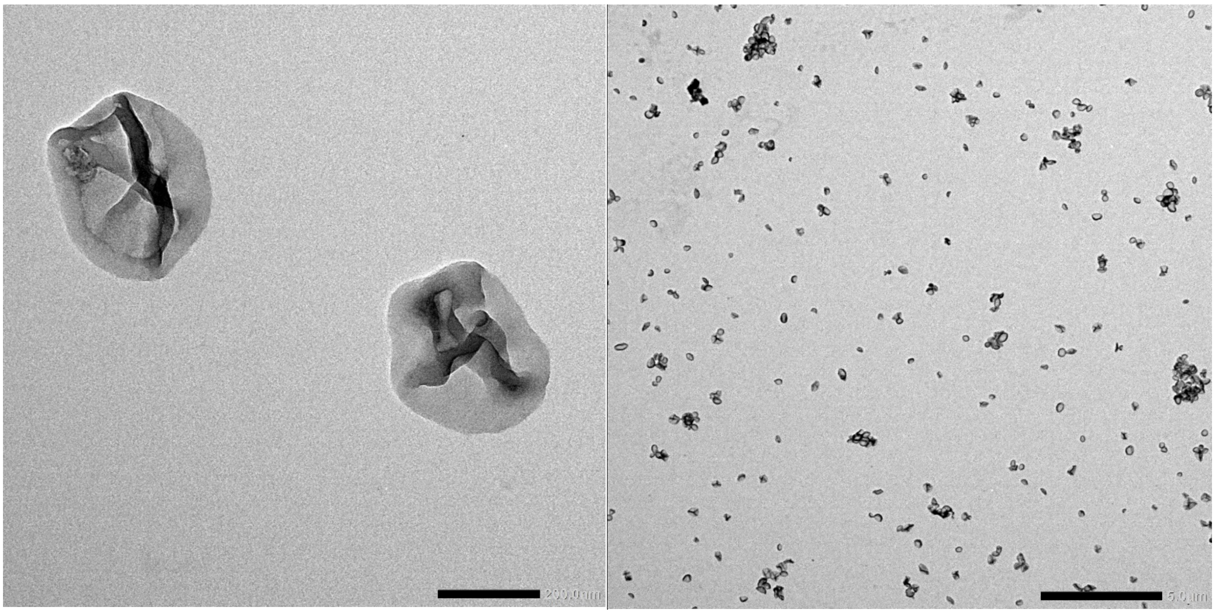

Figure S20: TEM images from water of MeO-PEG$_{44}$-*b*-P(S$_{141}$-*co*-4-VBA$_{18}$)$_{159}$ cross-linked polymersomes after 1 month. Scale bar: 200 nm (left), 5000 nm (right)

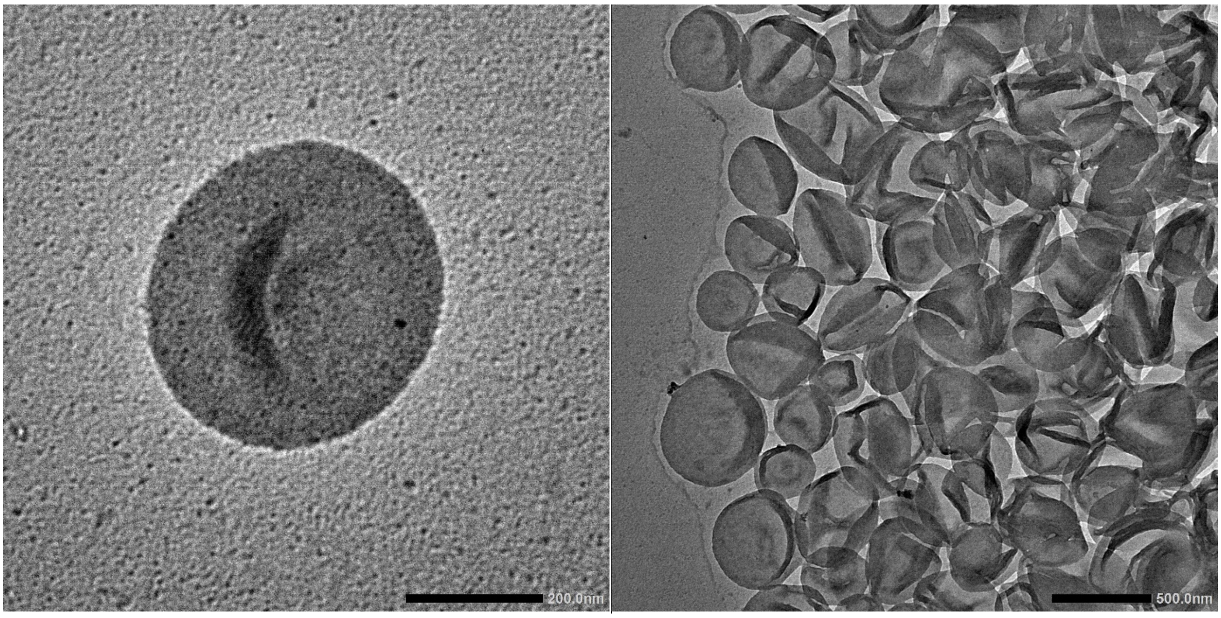

Figure S21: TEM images from THF of MeO-PEG$_{44}$-*b*-P(S$_{141}$-*co*-4-VBA$_{18}$)$_{159}$ cross-linked polymersomes after 1 hour. Scale bar: 200 nm (left), 500 nm (right)

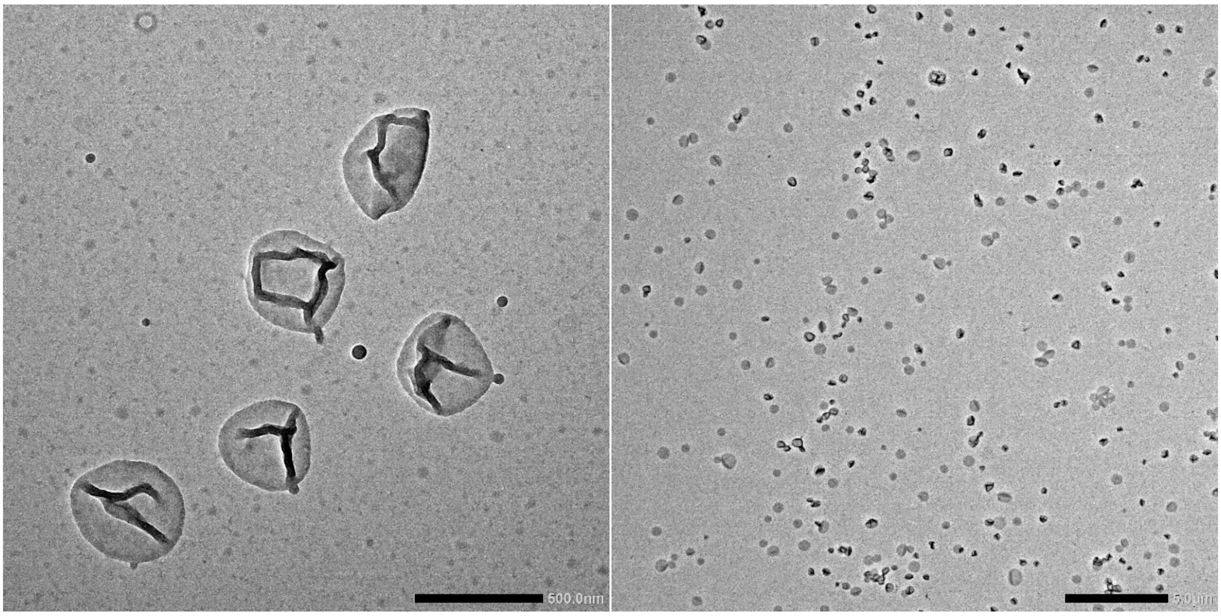

Figure S22: TEM images from THF of MeO-PEG$_{44}$-*b*-P(S$_{141}$-*co*-4-VBA$_{18}$)$_{159}$ cross-linked polymersomes after 1 day. Scale bar: 500 nm (left), 5000 nm (right)

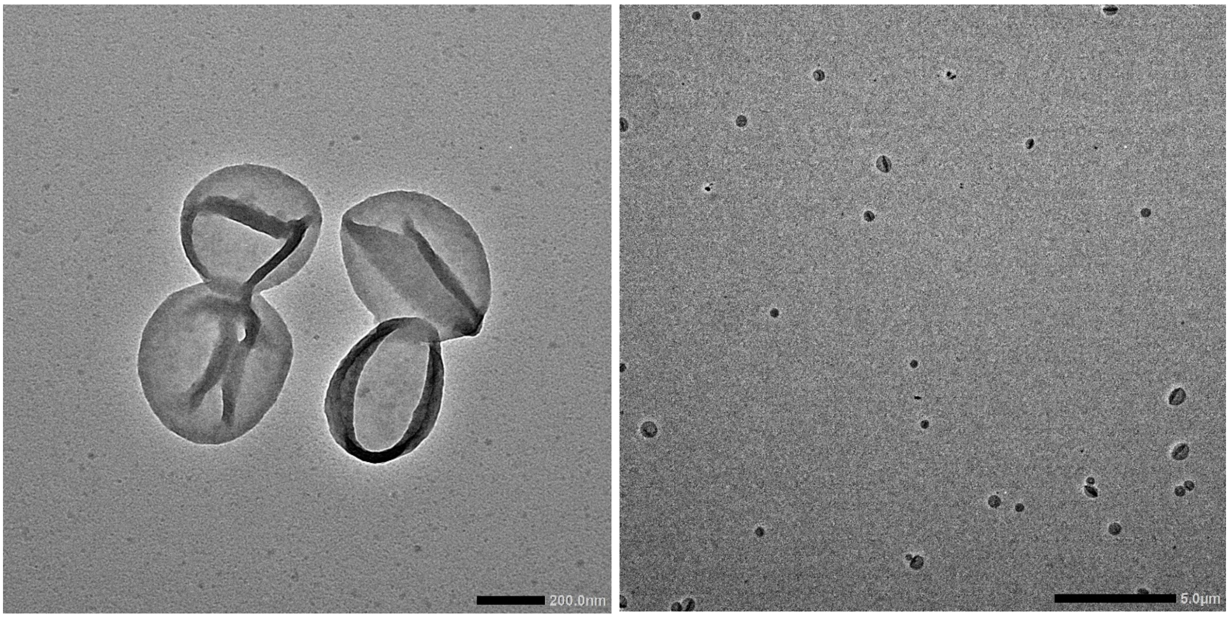

Figure S23: TEM images from THF of MeO-PEG$_{44}$-*b*-P(S$_{141}$-*co*-4-VBA$_{18}$)$_{159}$ cross-linked polymersomes after 1 week. Scale bar: 200 nm (left), 5000 nm (right)

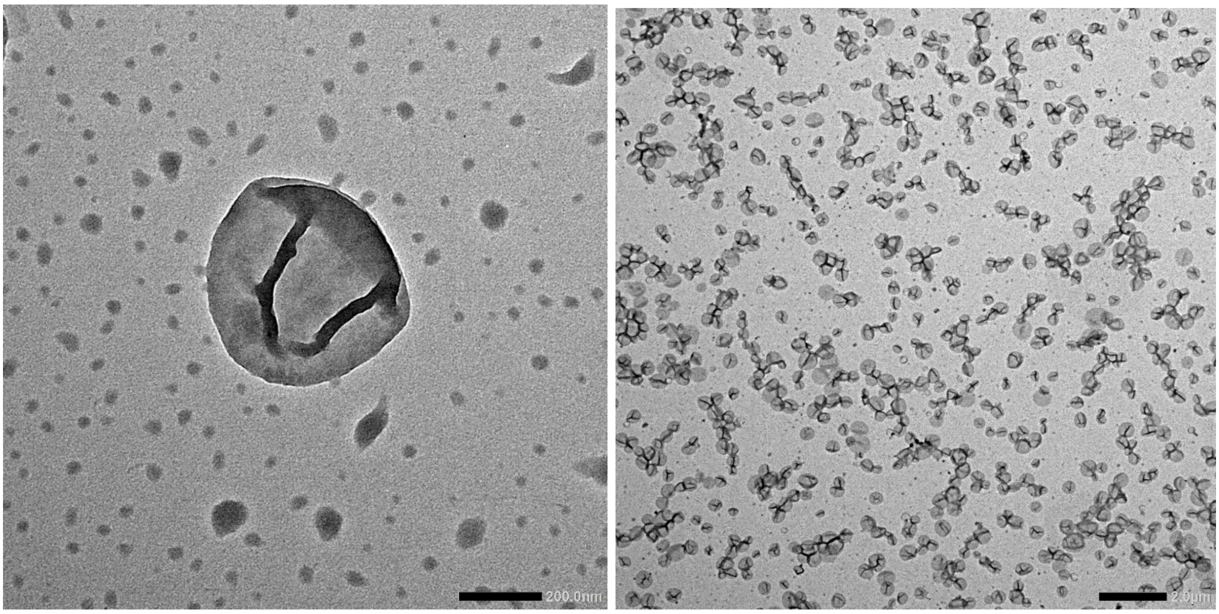

Figure S24: TEM images from THF of MeO-PEG$_{44}$-*b*-P(S$_{141}$-*co*-4-VBA$_{18}$)$_{159}$ cross-linked polymersomes after 1 month. Scale bar: 200 nm (left), 2000 nm (right)

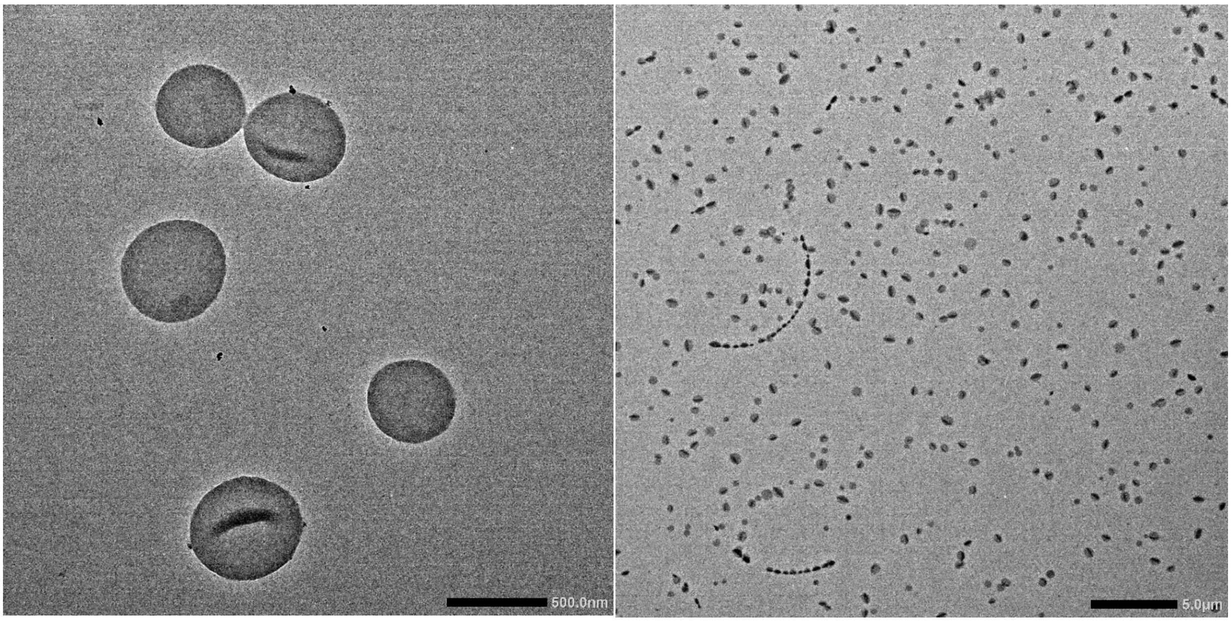

Figure S25: TEM images from DCM of MeO-PEG$_{44}$-*b*-P(S$_{141}$-*co*-4-VBA$_{18}$)$_{159}$ cross-linked polymersomes after 1 hour. Scale bar: 500 nm (left), 5000 nm (right)

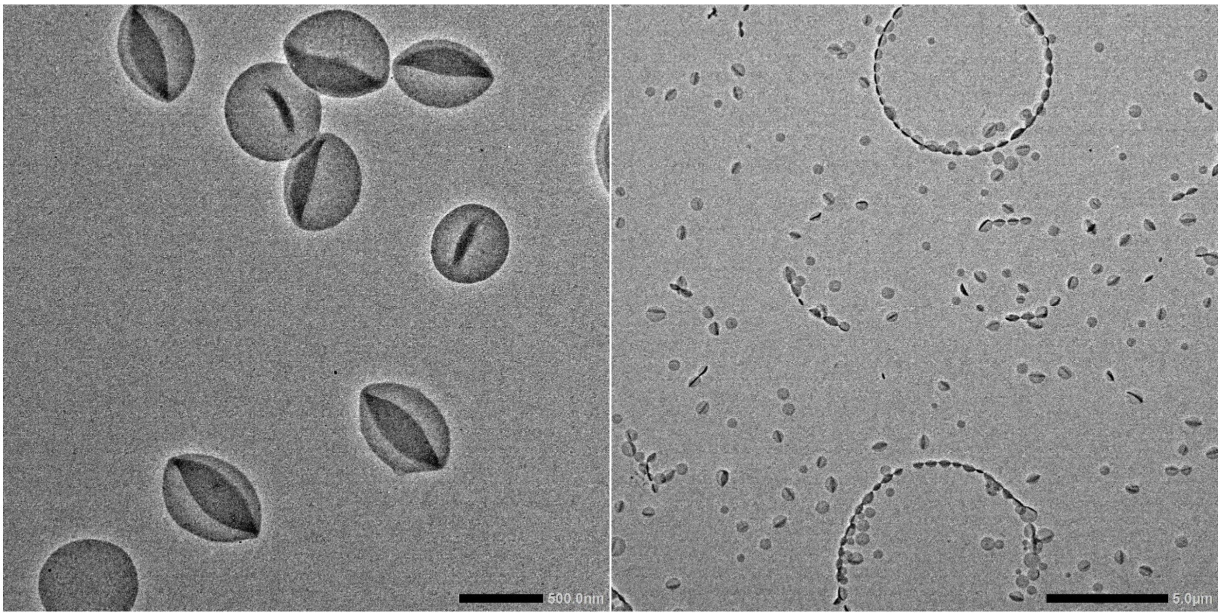

Figure S26: TEM images from DCM of MeO-PEG$_{44}$-*b*-P(S$_{141}$-*co*-4-VBA$_{18}$)$_{159}$ cross-linked polymersomes after 1 day. Scale bar: 500 nm (left), 5000 nm (right)

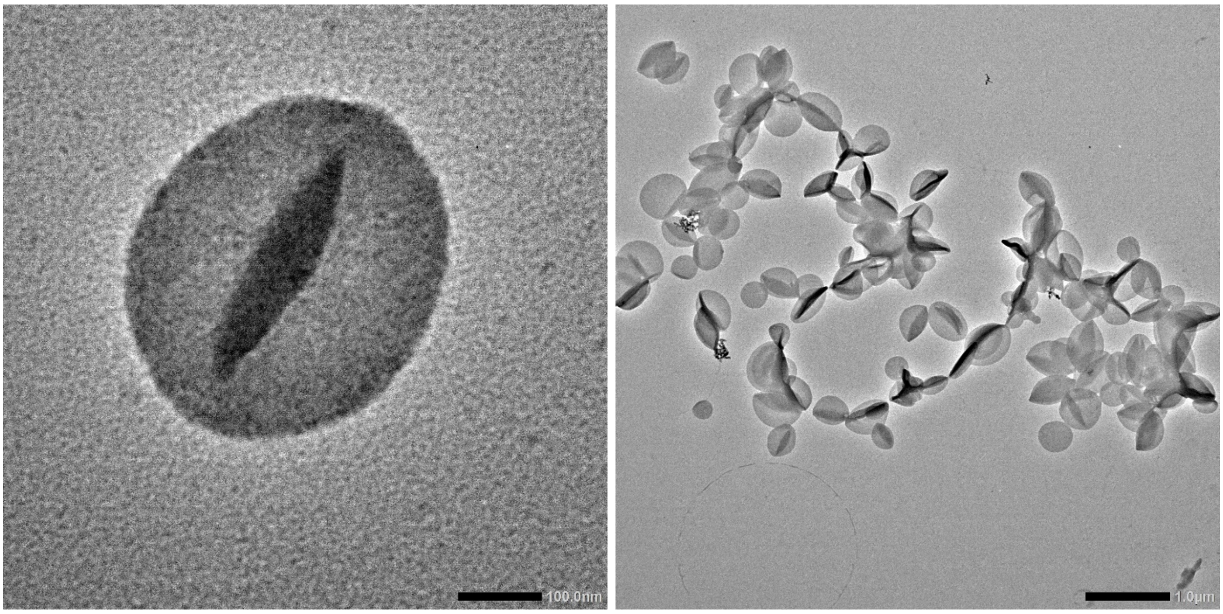

Figure S27: TEM images from DCM of MeO-PEG$_{44}$-*b*-P(S$_{141}$-*co*-4-VBA$_{18}$)$_{159}$ cross-linked polymersomes after 1 week. Scale bar: 500 nm (left), 1000 nm (right)

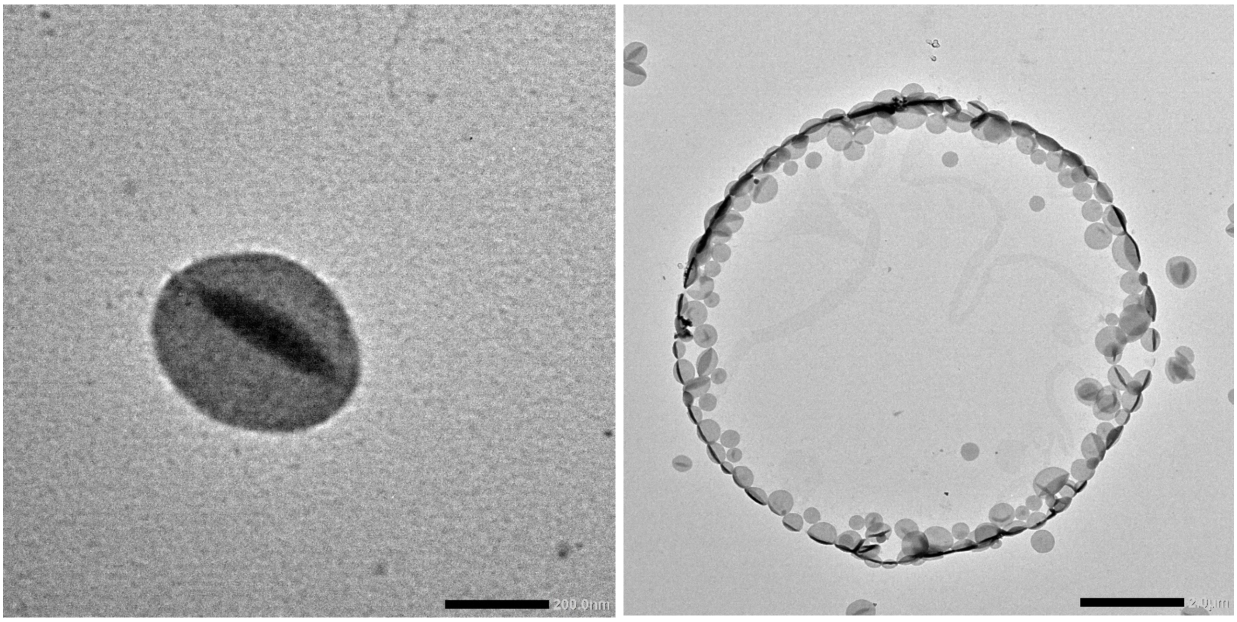

Figure S28: TEM images from DCM of MeO-PEG$_{44}$-*b*-P(S$_{141}$-*co*-4-VBA$_{18}$)$_{159}$ cross-linked polymersomes after 1 month. Scale bar: 200 nm (left), 2000 nm (right)

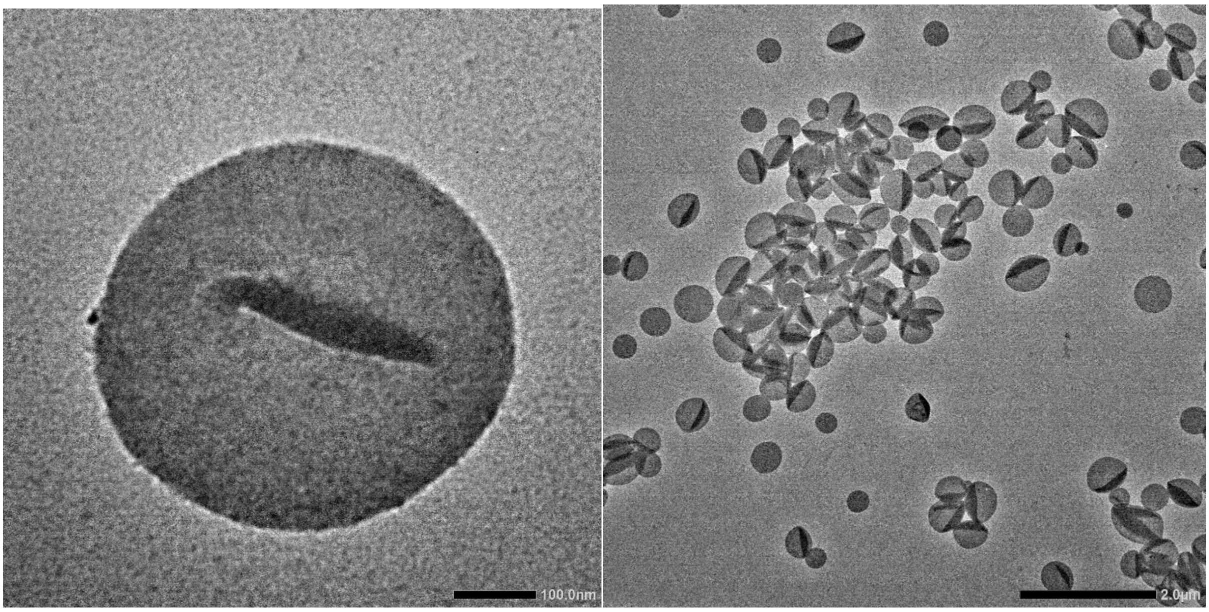

Figure S29: TEM images from toluene of MeO-PEG$_{44}$-*b*-P(S$_{141}$-*co*-4-VBA$_{18}$)$_{159}$ cross-linked polymersomes after 1 hour. Scale bar: 100 nm (left), 2000 nm (right)

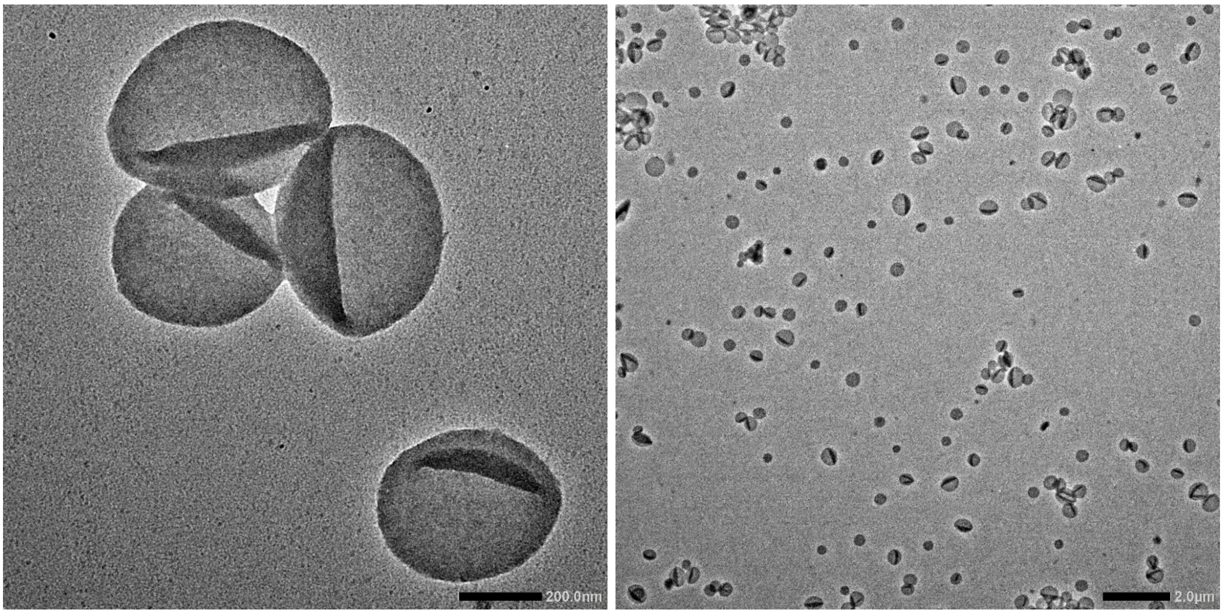

Figure S30: TEM images from toluene of MeO-PEG$_{44}$-*b*-P(S$_{141}$-*co*-4-VBA$_{18}$)$_{159}$ cross-linked polymersomes after 1 day. Scale bar: 200 nm (left), 2000 nm (right)

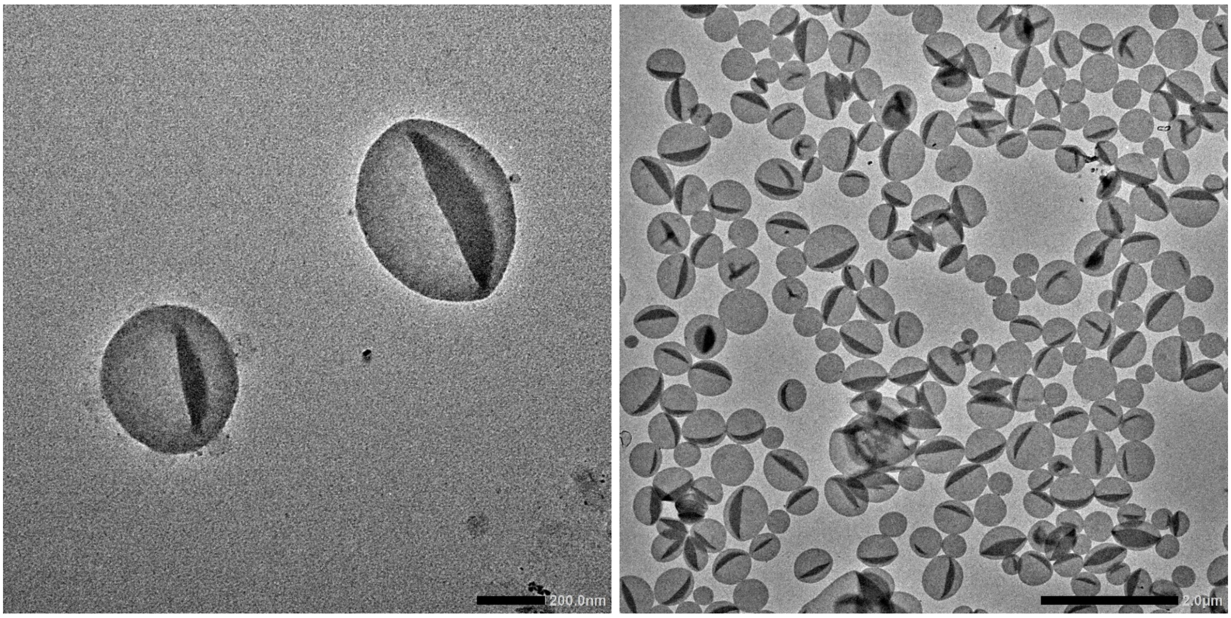

Figure S31: TEM images from toluene of MeO-PEG$_{44}$-*b*-P(S$_{141}$-*co*-4-VBA$_{18}$)$_{159}$ cross-linked polymersomes after 1 week. Scale bar: 200 nm (left), 2000 nm (right)

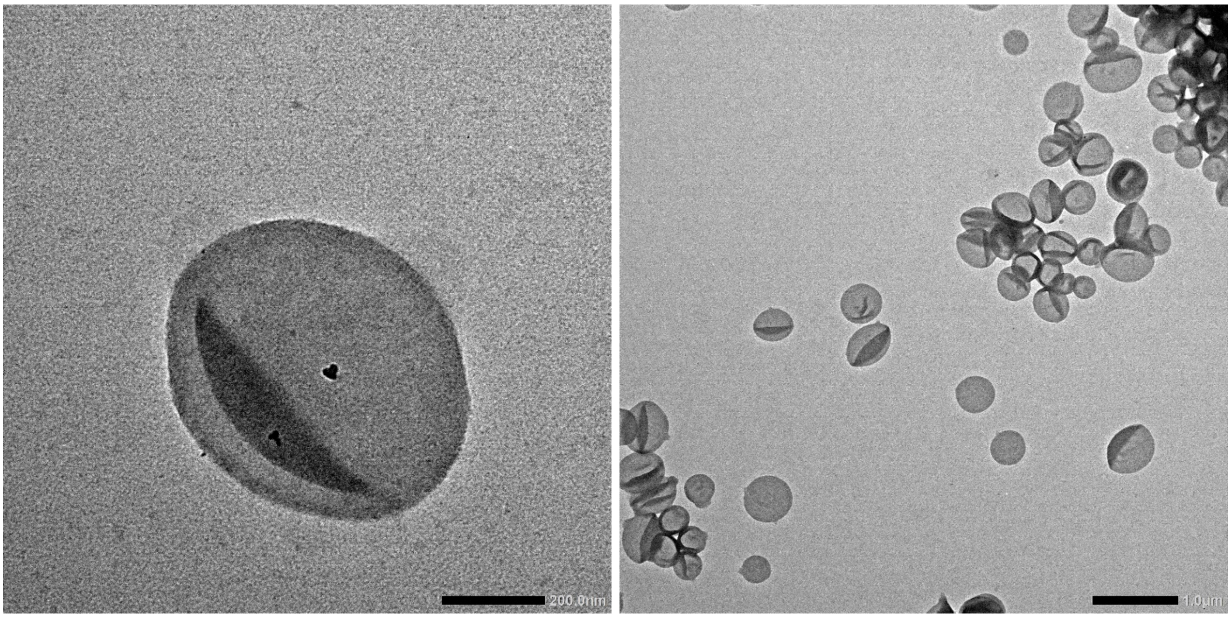

Figure S32: TEM images from toluene of MeO-PEG$_{44}$-*b*-P(S$_{141}$-*co*-4-VBA$_{18}$)$_{159}$ cross-linked polymersomes after 1 month. Scale bar: 200 nm (left), 1000 nm (right)

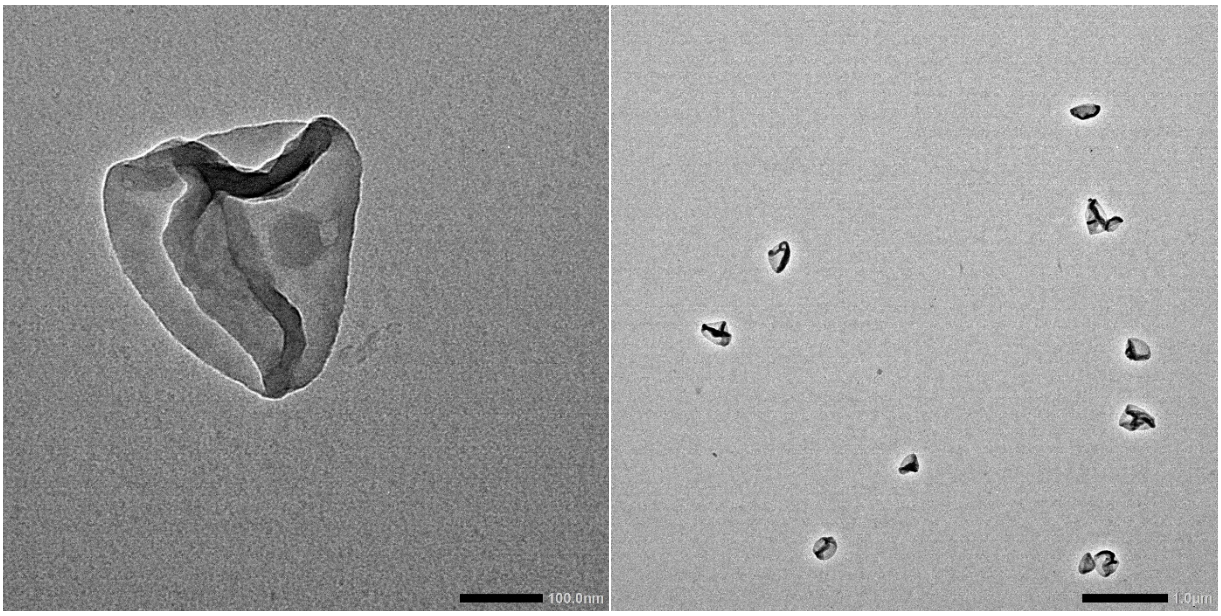

Figure S33: TEM images from ethanol of MeO-PEG$_{44}$-*b*-P(S$_{141}$-*co*-4-VBA$_{18}$)$_{159}$ cross-linked polymersomes after 1 hour. Scale bar: 100 nm (left), 1000 nm (right)

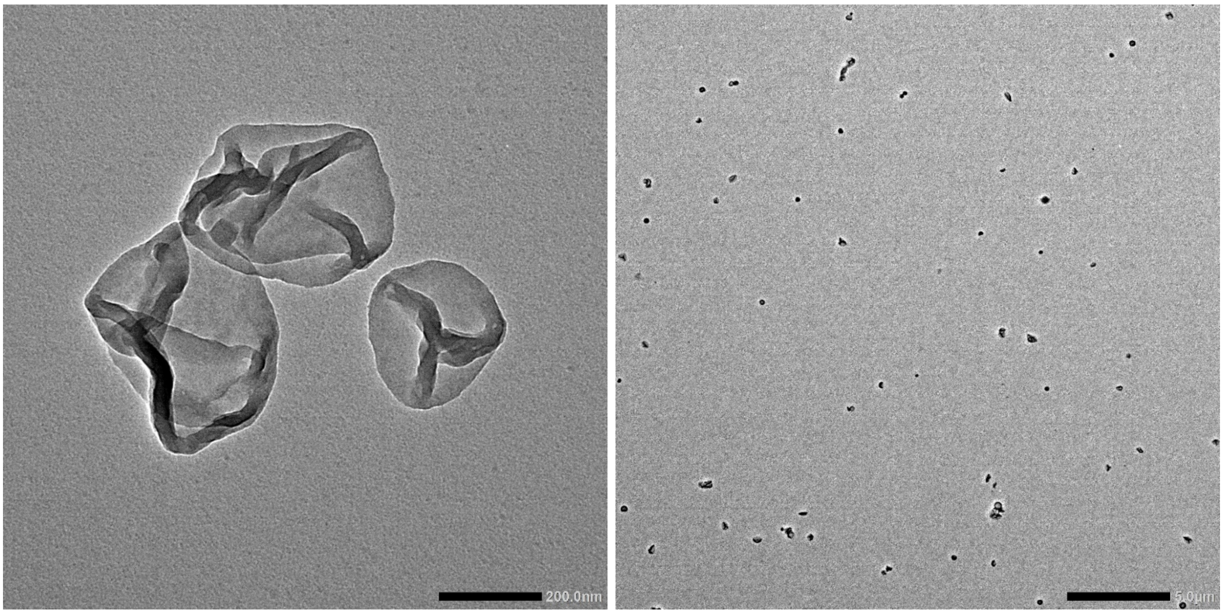

Figure S34: TEM images from ethanol of MeO-PEG$_{44}$-*b*-P(S$_{141}$-*co*-4-VBA$_{18}$)$_{159}$ cross-linked polymersomes after 1 day. Scale bar: 200 nm (left), 5000 nm (right)

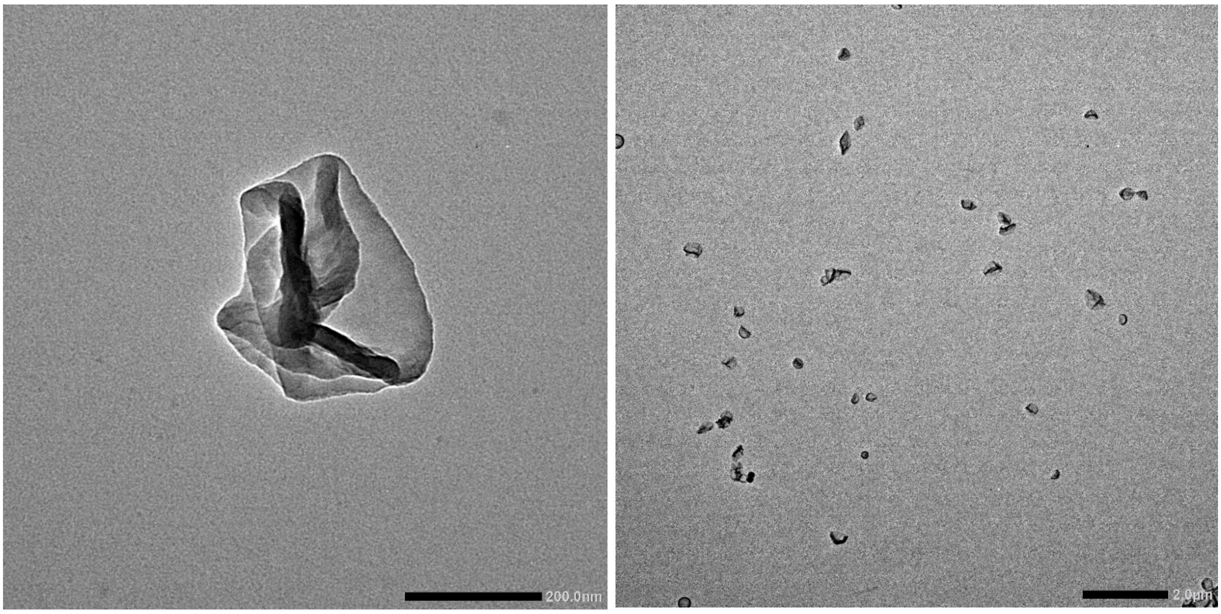

Figure S35: TEM images from ethanol of MeO-PEG$_{44}$-*b*-P(S$_{141}$-*co*-4-VBA$_{18}$)$_{159}$ cross-linked polymersomes after 1 week. Scale bar: 200 nm (left), 2000 nm (right)

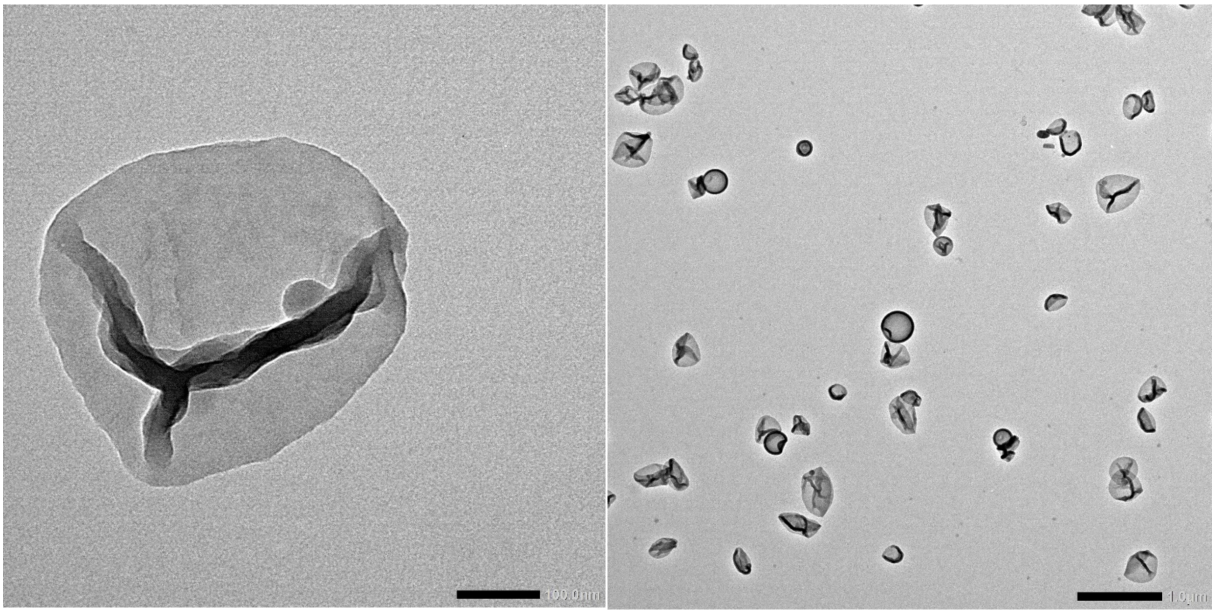

Figure S36: TEM images from ethanol of MeO-PEG$_{44}$-*b*-P(S$_{141}$-*co*-4-VBA$_{18}$)$_{159}$ cross-linked polymersomes after 1 month. Scale bar: 100 nm (left), 1000 nm (right)

7. DLS data of Polymersome Solvent Stability Tests

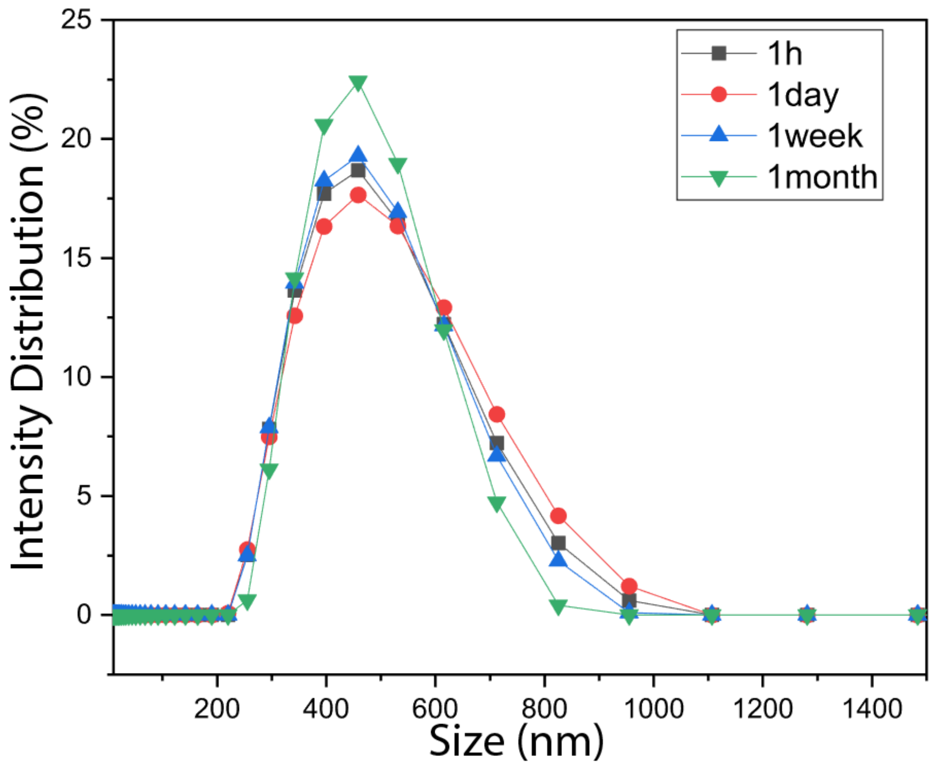

Figure S37: Size distribution of MeO-PEG$_{44}$-*b*-P(S$_{141}$-*co*-4-VBA$_{18}$)$_{159}$ cross-linked polymersomes in water over time, characterized by DLS.

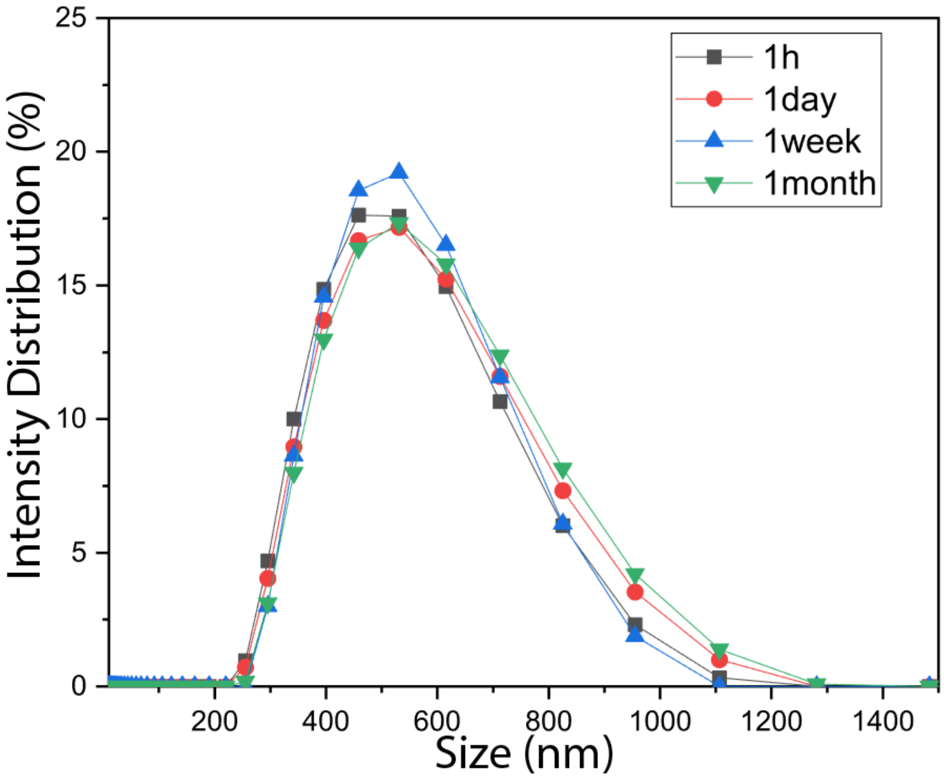

Figure S38: Size distribution of MeO-PEG$_{44}$-*b*-P(S$_{141}$-*co*-4-VBA$_{18}$)$_{159}$ cross-linked polymersomes in THF over time, characterized by DLS.

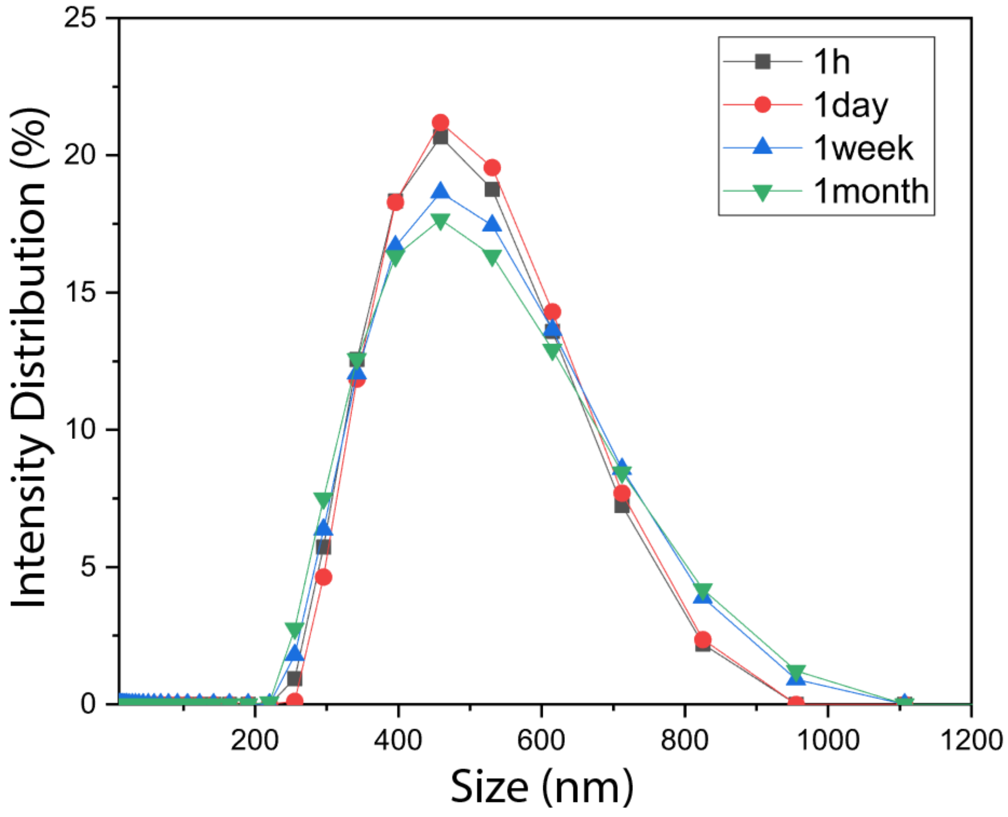

Figure S39: Size distribution of MeO-PEG$_{44}$-*b*-P(S$_{141}$-*co*-4-VBA$_{18}$)$_{159}$ cross-linked polymersomes in DCM over time, characterized by DLS.

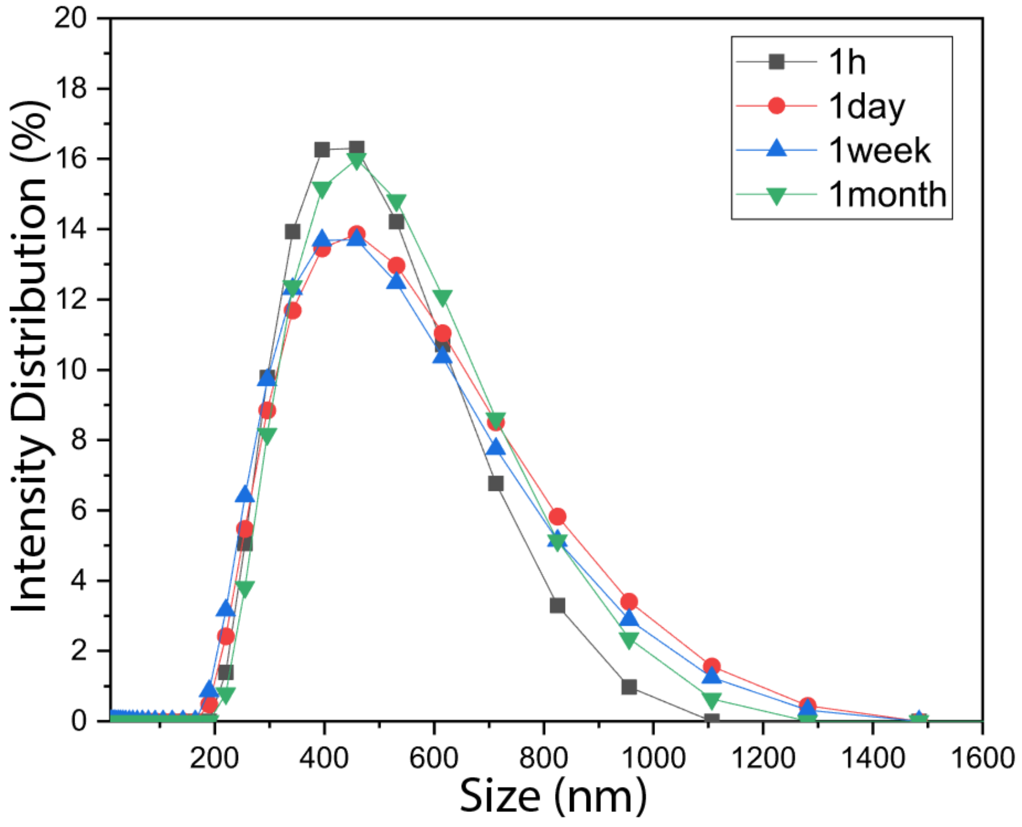

Figure S40: Size distribution of MeO-PEG$_{44}$-*b*-P(S$_{141}$-*co*-4-VBA$_{18}$)$_{159}$ cross-linked polymersomes in ethanol over time, characterized by DLS.

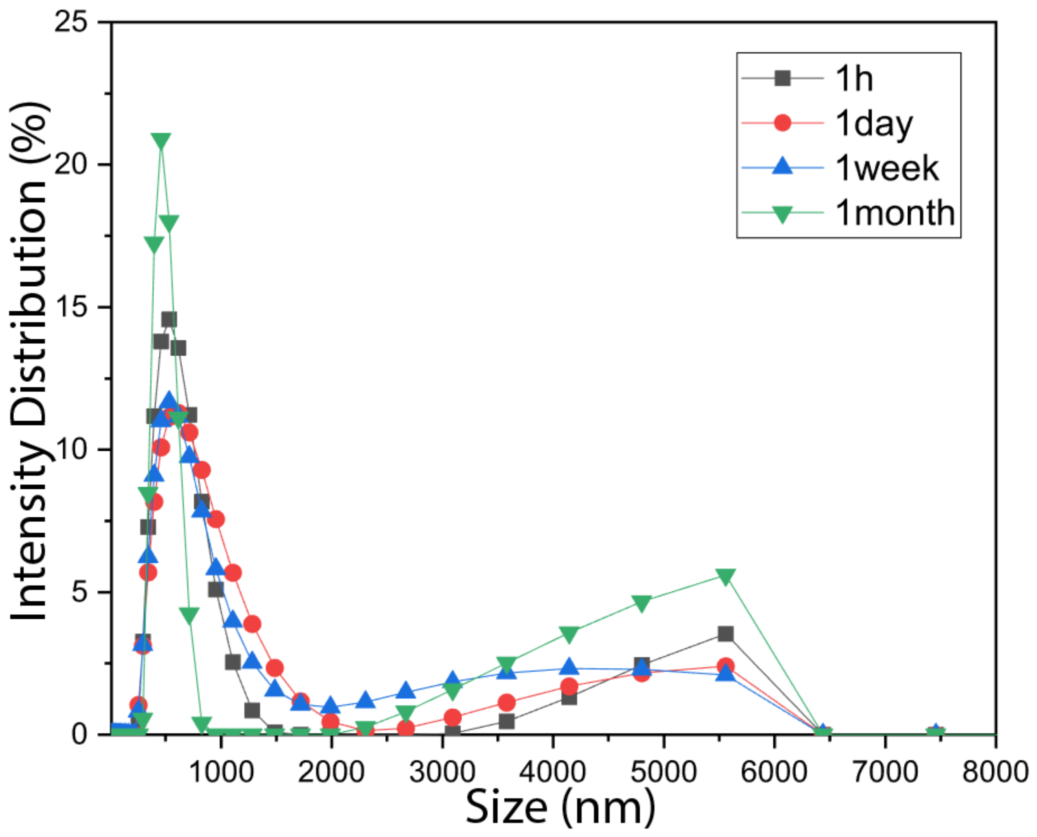

Figure S41: Size distribution of MeO-PEG$_{44}$-*b*-P(S$_{141}$-*co*-4-VBA$_{18}$)$_{159}$ cross-linked polymersomes in toluene over time, characterized by DLS.

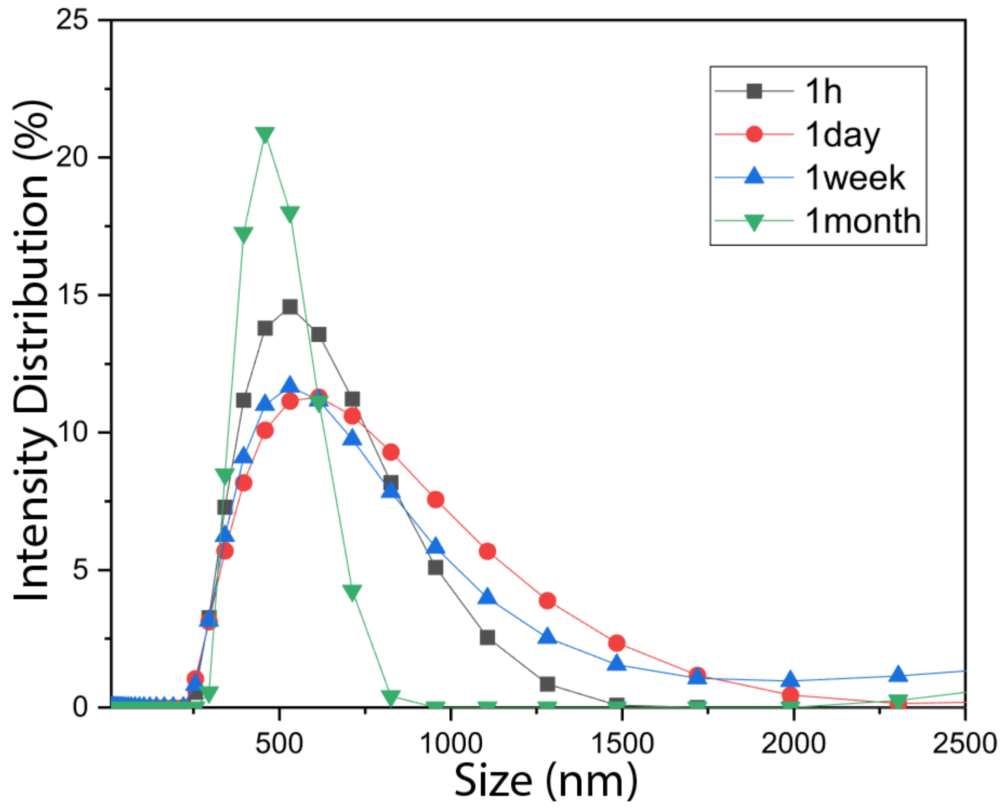

Figure S42: Size distribution of MeO-PEG$_{44}$-*b*-P(S$_{141}$-*co*-4-VBA$_{18}$)$_{159}$ cross-linked polymersomes in toluene over time, characterized by DLS. Zoom-in of Figure S38.

8. Cryo-TEM images of DBCO-containing Cross-Linked Polymersomes

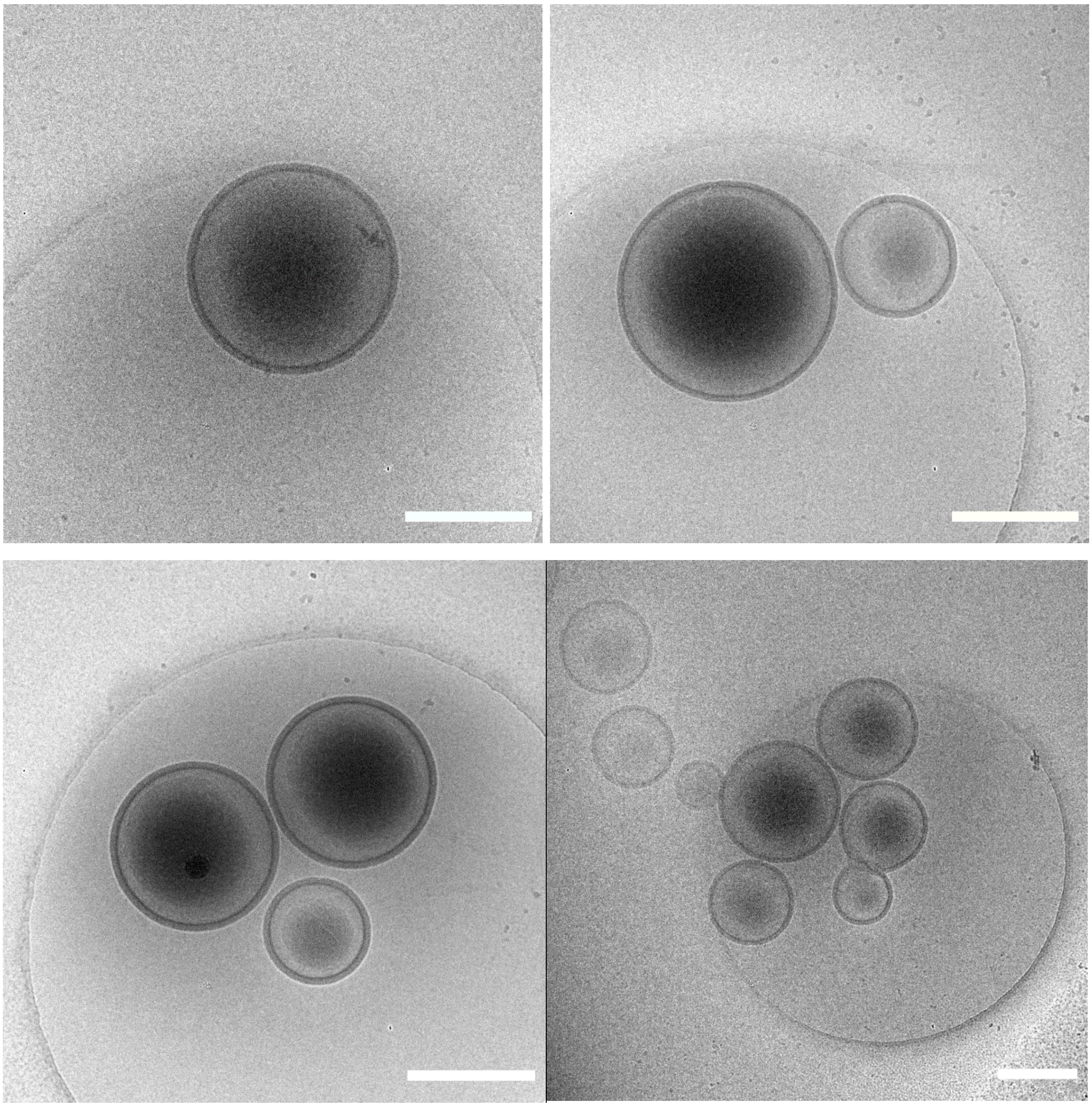

Figure S43: Cryo-TEM images of MeO-PEG$_{44}$-*b*-P(S$_{141}$-*co*-4-VBA$_{18}$)$_{159}$ cross-linked polymersomes containing 10% of DBCO functionality. Scale bar: 500 nm

9. Fluorescence data of DBCO + 3-azido-7-hydroxycoumarin Click Reaction

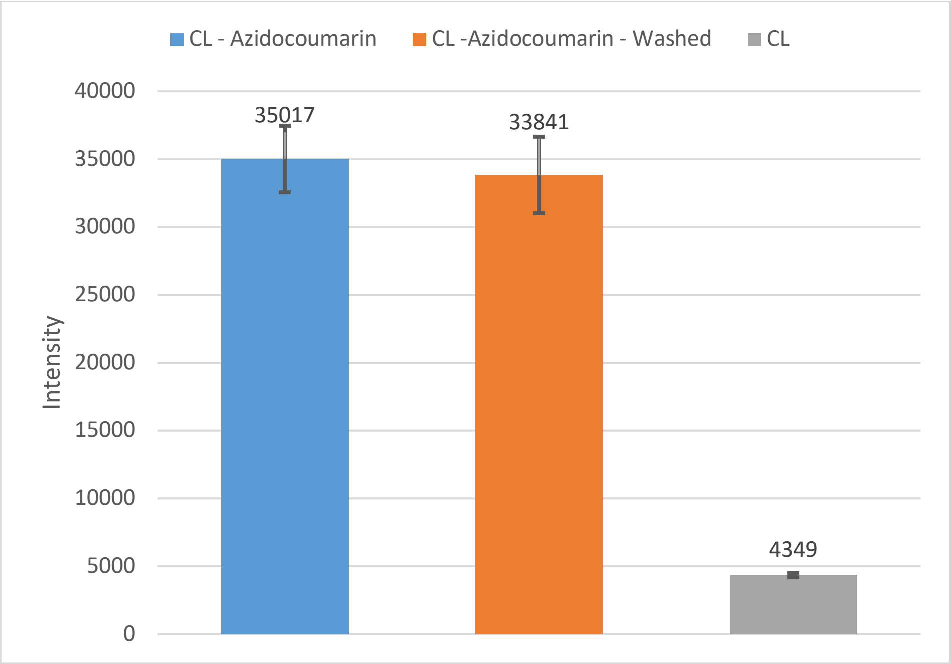

Figure S44: Plot of fluorescence measurements of cross-linked MeO-PEG$_{44}$-*b*-P(S$_{141}$-*co*-4-VBA$_{18}$)$_{159}$ polymersomes containing 10% of DBCO functionality from DBCO-PEG$_{44}$-*b*-PS$_{189}$ after overnight click reaction with 3-azido-7-hydroxycoumarin, diluted 50x from regular concentration. Comparison is made between polymersomes washed 3x with THF and unwashed, to potentially remove any DBCO-polymer in the self-assembly. Cross-linked polymersomes without reaction with 3-azido-7-hydroxycoumarin are used as a blank. Error bars are based on the standard deviation of ten measurements Excitation was performed using a filter for 430 nm (20 nm bandwidth) and emission was measured with a filter for 485 nm (20 nm bandwidth). Relative fluorescence is given, as a relative comparison was all that was desired. Similar fluorescence intensities between washed and unwashed indicate that DBCO-PEG$_{44}$-*b*-PS$_{189}$ remains in the polymersome membrane even without have cross-linkable units.

10. TEM images of Polymersomes with Holes

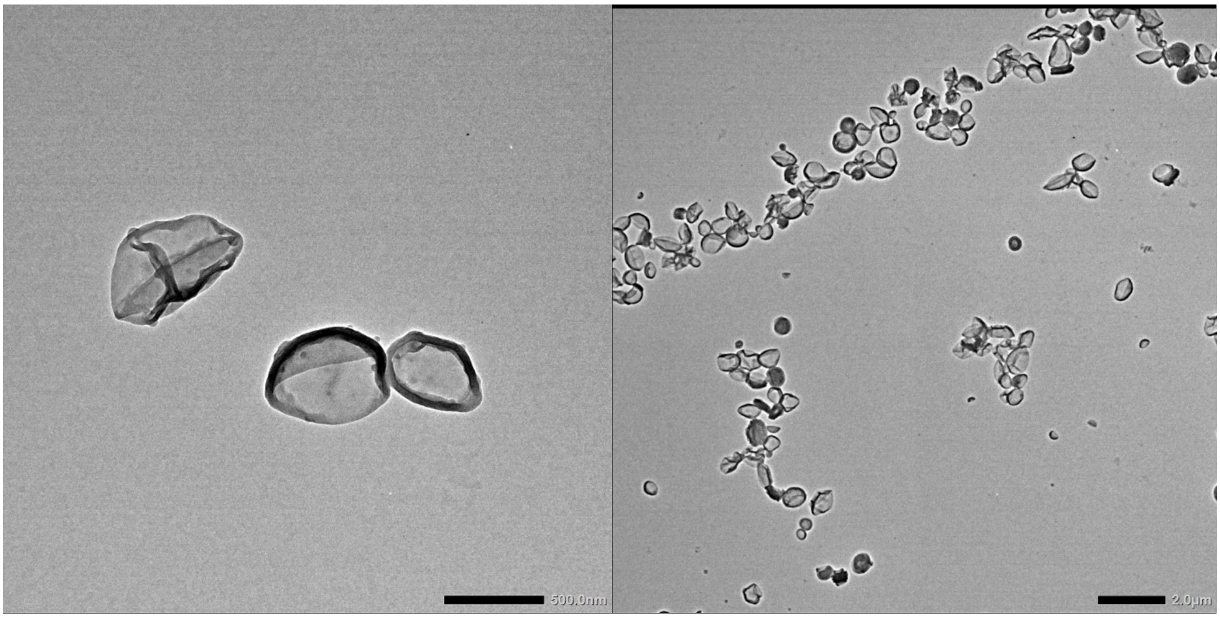

Figure S45: TEM images from water of 90% MeO-PEG$_{44}$-*b*-P(S$_{141}$-*co*-4-VBA$_{18}$)$_{159}$ cross-linked polymersomes containing 10% PEG-*b*-PS. Scale bar: 500 nm (left), 2000 nm (right)

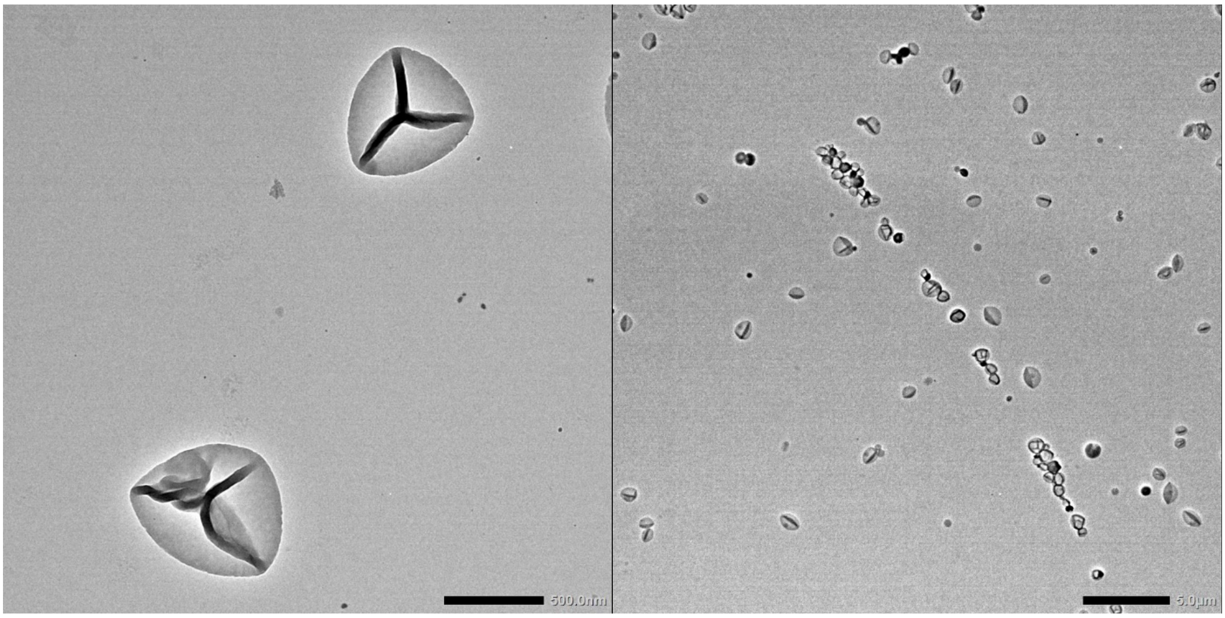

Figure S46: TEM images from THF of 90% MeO-PEG$_{44}$-*b*-P(S$_{141}$-*co*-4-VBA$_{18}$)$_{159}$ cross-linked polymersomes containing 10% PEG-*b*-PS. Scale bar: 500 nm (left), 5000 nm (right)

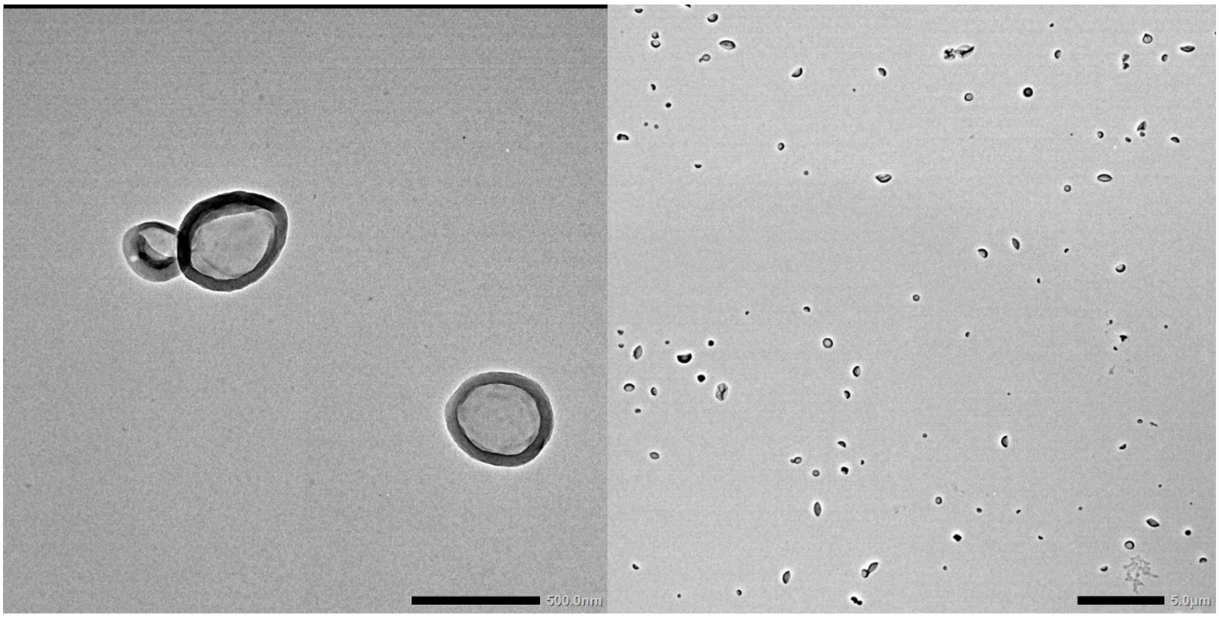

Figure S47: TEM images from water of 80% MeO-PEG$_{44}$-*b*-P(S$_{141}$-*co*-4-VBA$_{18}$)$_{159}$ cross-linked polymersomes containing 20% PEG-*b*-PS. Scale bar: 500 nm (left), 5000 nm (right)

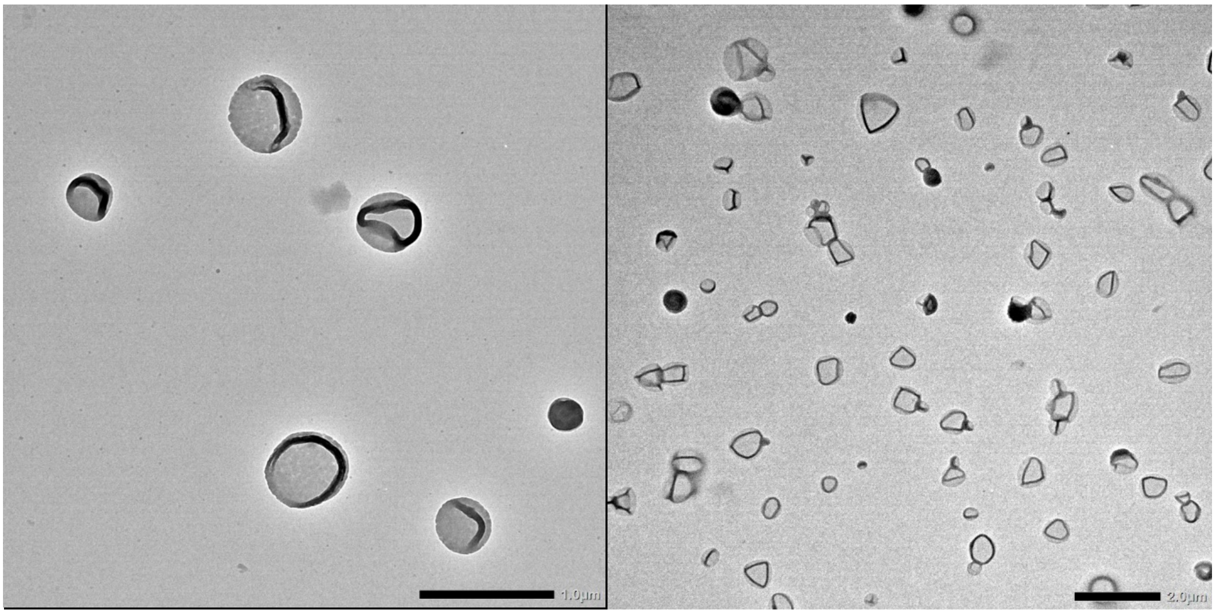

Figure S48: TEM images from THF of 80% MeO-PEG$_{44}$-*b*-P(S$_{141}$-*co*-4-VBA$_{18}$)$_{159}$ cross-linked polymersomes containing 20% PEG-*b*-PS. Scale bar: 1000 nm (left), 2000 nm (right)

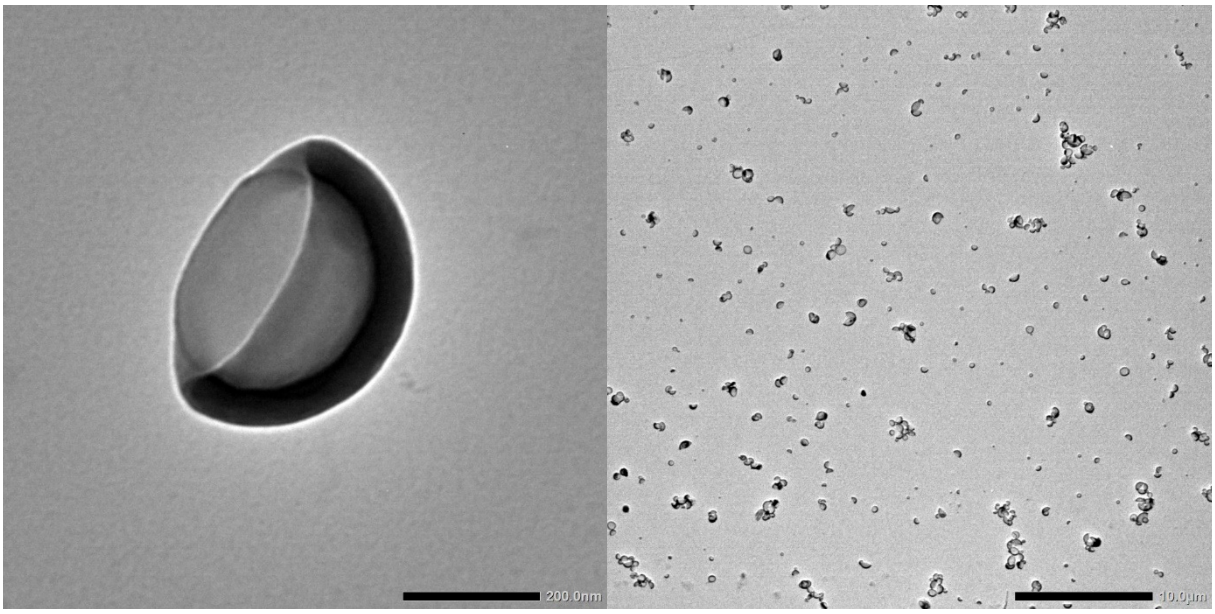

Figure S49: TEM images from water of 70% MeO-PEG$_{44}$-*b*-P(S$_{141}$-*co*-4-VBA$_{18}$)$_{159}$ cross-linked polymersomes containing 30% PEG-*b*-PS. Scale bar: 200 nm (left), 10000 nm (right)

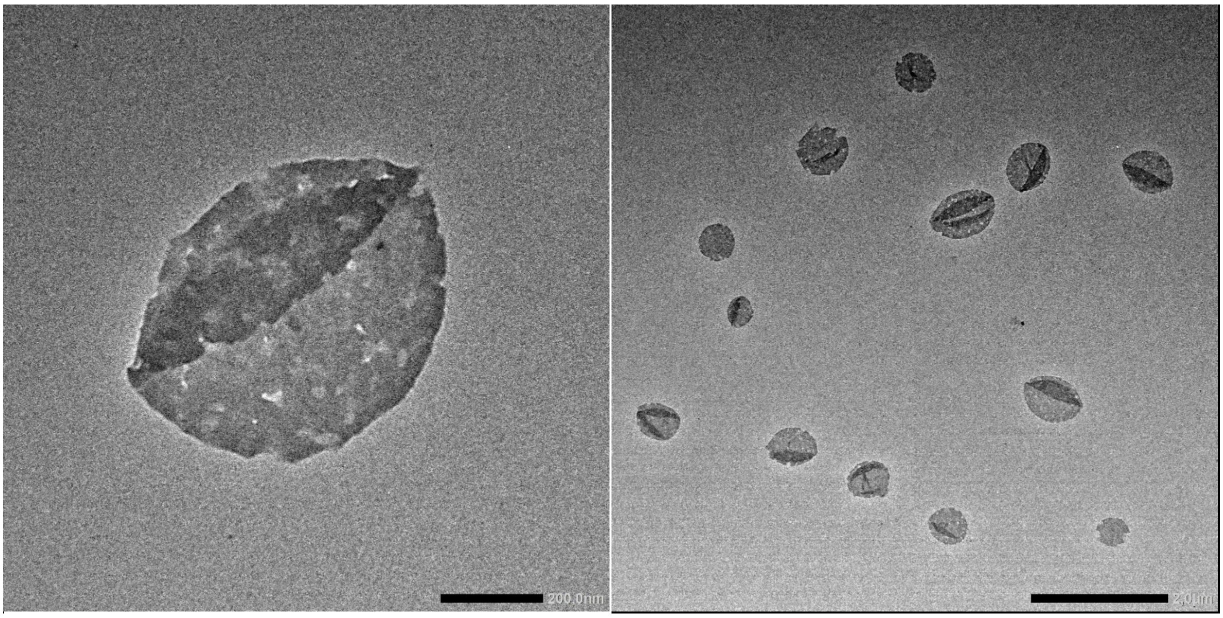

Figure S50: TEM images from THF of 70% MeO-PEG$_{44}$-*b*-P(S$_{141}$-*co*-4-VBA$_{18}$)$_{159}$ cross-linked polymersomes containing 30% PEG-*b*-PS. Scale bar: 200 nm (left), 2000 nm (right)

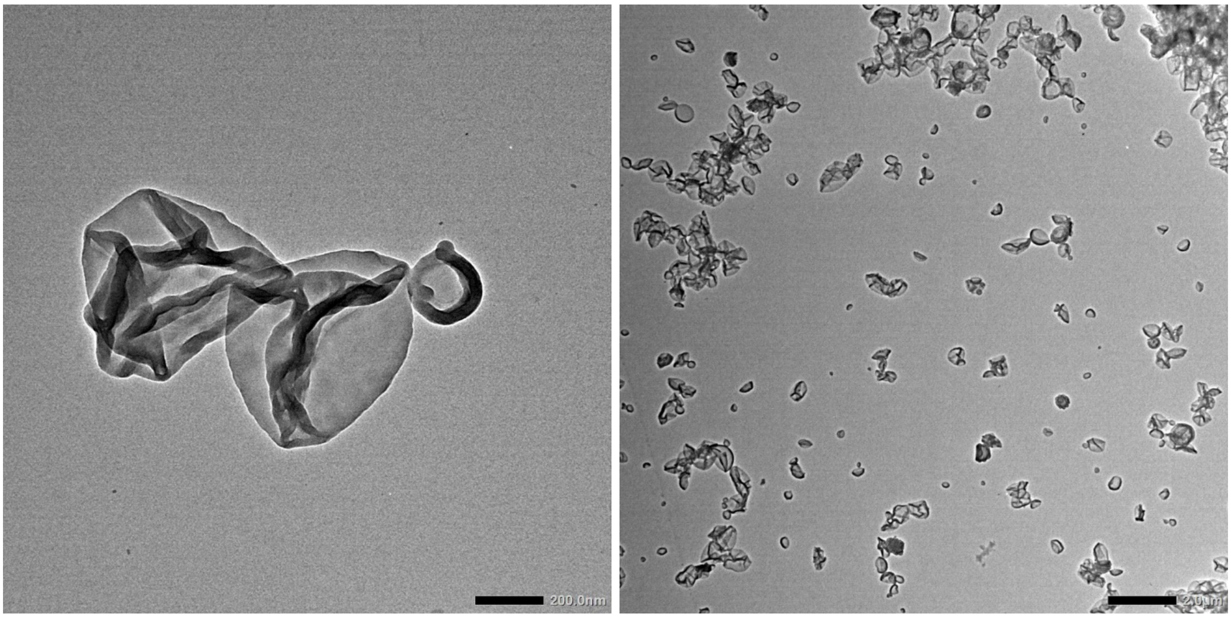

Figure S51: TEM images from water of 60% MeO-PEG$_{44}$-*b*-P(S$_{141}$-*co*-4-VBA$_{18}$)$_{159}$ cross-linked polymersomes containing 40% PEG-*b*-PS. Scale bar: 200 nm (left), 2000 nm (right)

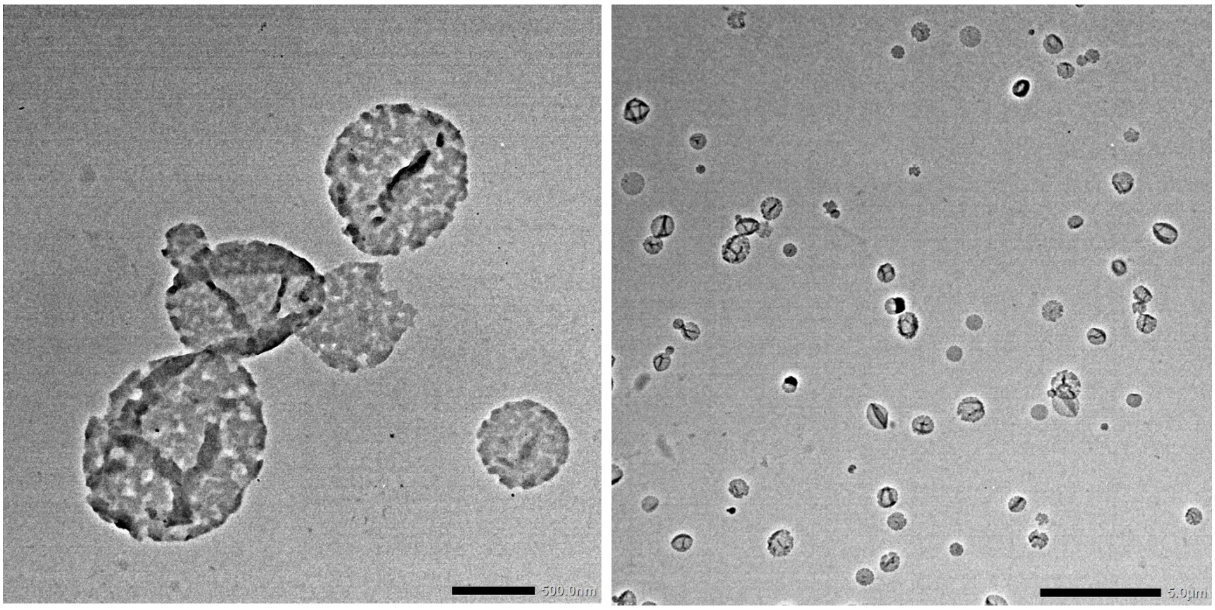

Figure S52: TEM images from THF of 60% MeO-PEG$_{44}$-*b*-P(S$_{141}$-*co*-4-VBA$_{18}$)$_{159}$ cross-linked polymersomes containing 40% PEG-*b*-PS. Scale bar: 500 nm (left), 5000 nm (right)

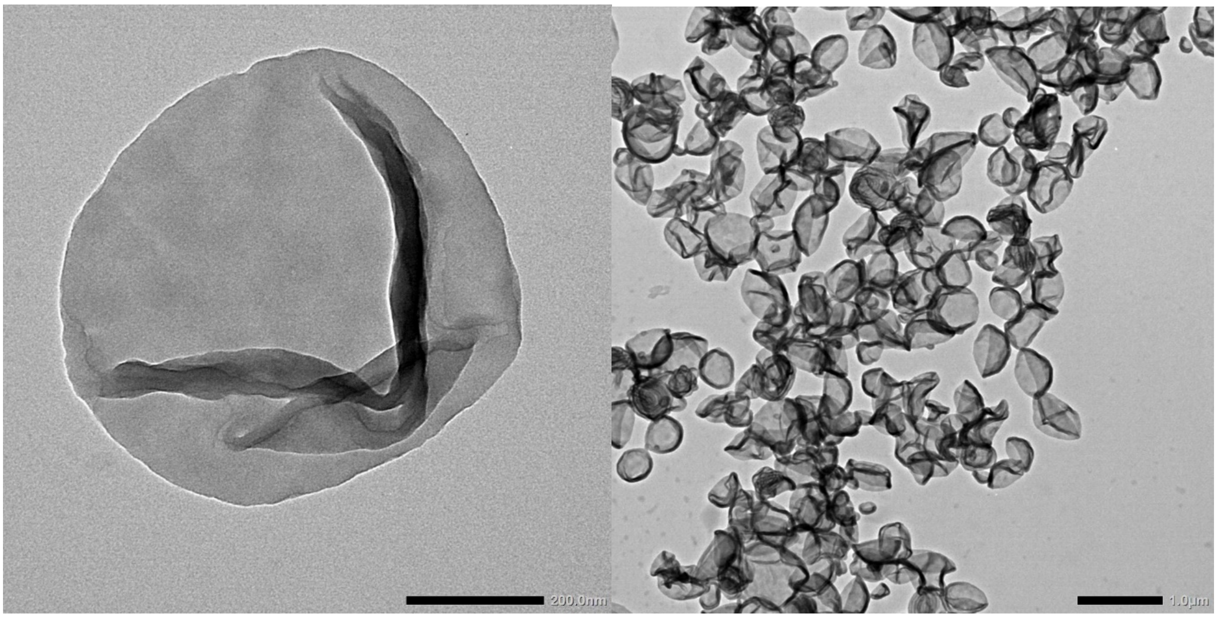

Figure S53: TEM images from water of 50% MeO-PEG$_{44}$-*b*-P(S$_{141}$-*co*-4-VBA$_{18}$)$_{159}$ cross-linked polymersomes containing 50% PEG-*b*-PS. Scale bar: 200 nm (left), 1000 nm (right)

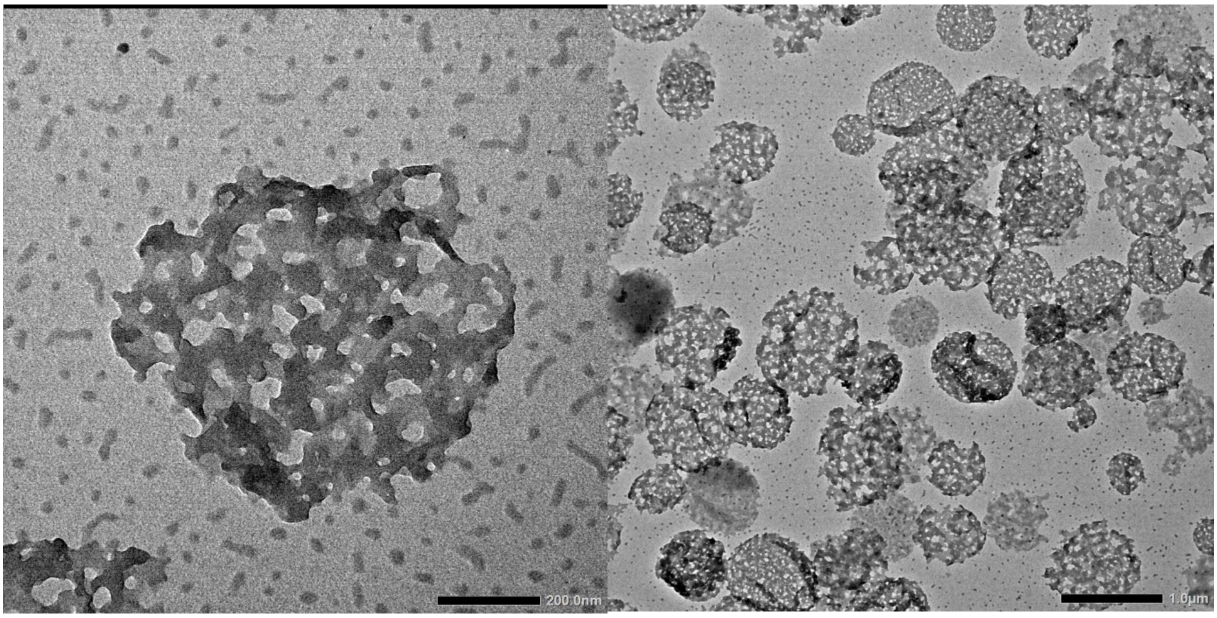

Figure S54: TEM images from THF of 50% MeO-PEG$_{44}$-*b*-P(S$_{141}$-*co*-4-VBA$_{18}$)$_{159}$ cross-linked polymersomes containing 50% PEG-*b*-PS. Scale bar: 200 nm (left), 1000 nm (right)

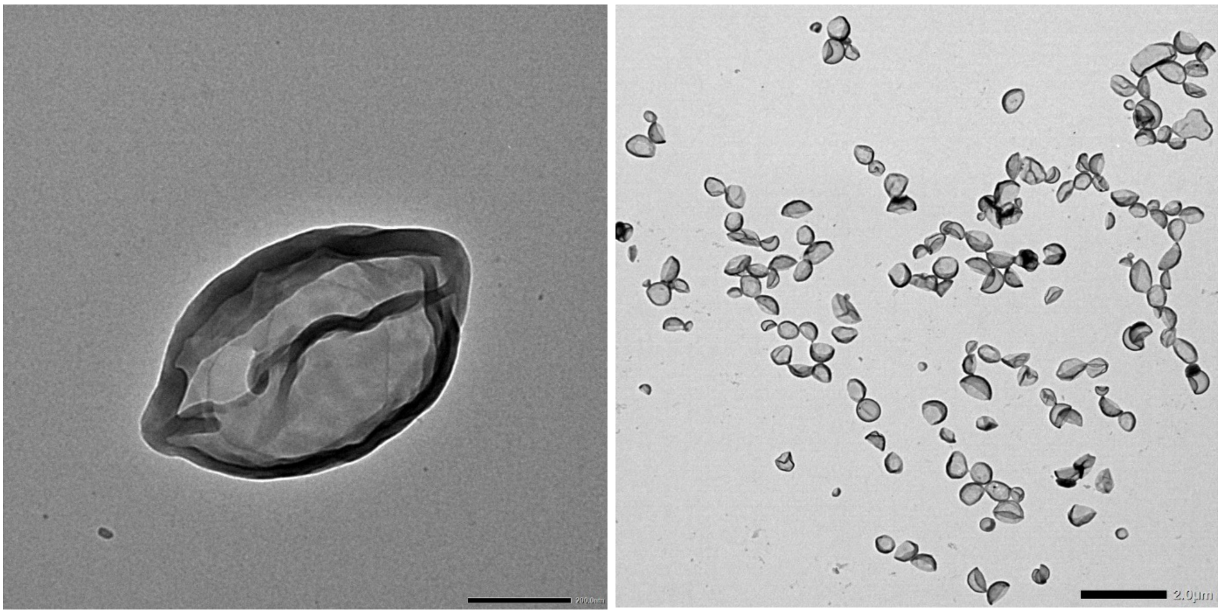

Figure S55: TEM images from water of 40% MeO-PEG_{44}-*b*-P(S_{141}-*co*-4-VBA_{18})$_{159}$ cross-linked polymersomes containing 60% PEG-*b*-PS. Scale bar: 200 nm (left), 1000 nm (right)

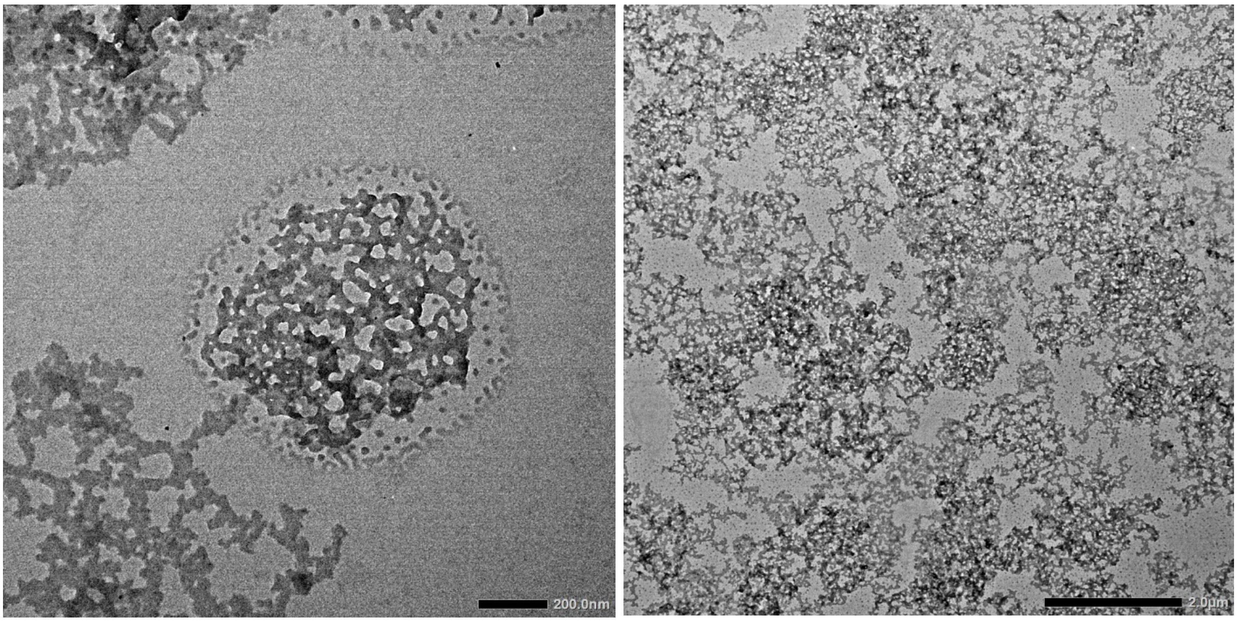

Figure S56: TEM images from THF of 40% MeO-PEG_{44}-*b*-P(S_{141}-*co*-4-VBA_{18})$_{159}$ cross-linked polymersomes containing 60% PEG-*b*-PS. Scale bar: 200 nm (left), 2000 nm (right)

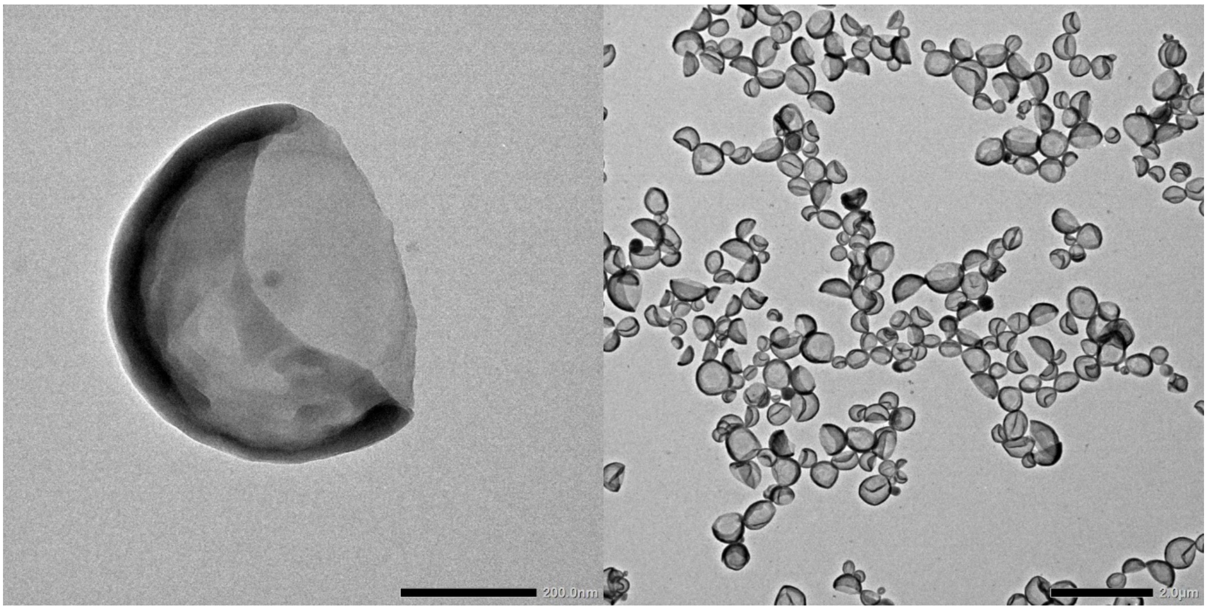

Figure S57: TEM images from water of 30% MeO-PEG$_{44}$-*b*-P(S$_{141}$-*co*-4-VBA$_{18}$)$_{159}$ cross-linked polymersomes containing 70% PEG-*b*-PS. Scale bar: 200 nm (left), 2000 nm (right)

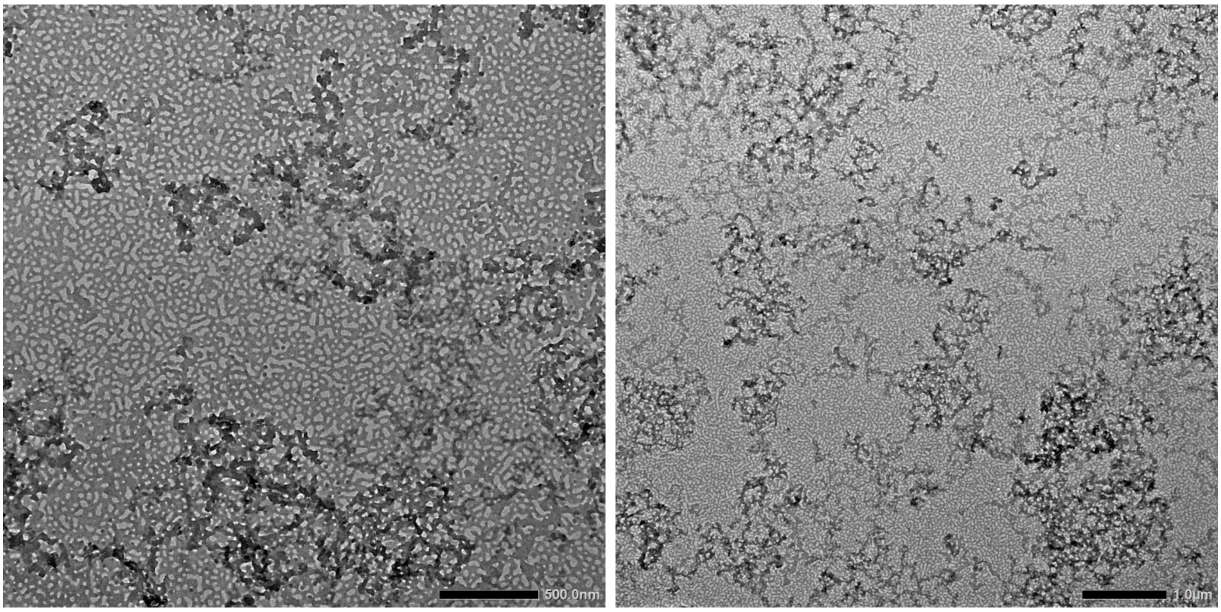

Figure S58: TEM images from THF of 30% MeO-PEG$_{44}$-*b*-P(S$_{141}$-*co*-4-VBA$_{18}$)$_{159}$ cross-linked polymersomes containing 70% PEG-*b*-PS. Scale bar: 500 nm (left), 1000 nm (right)

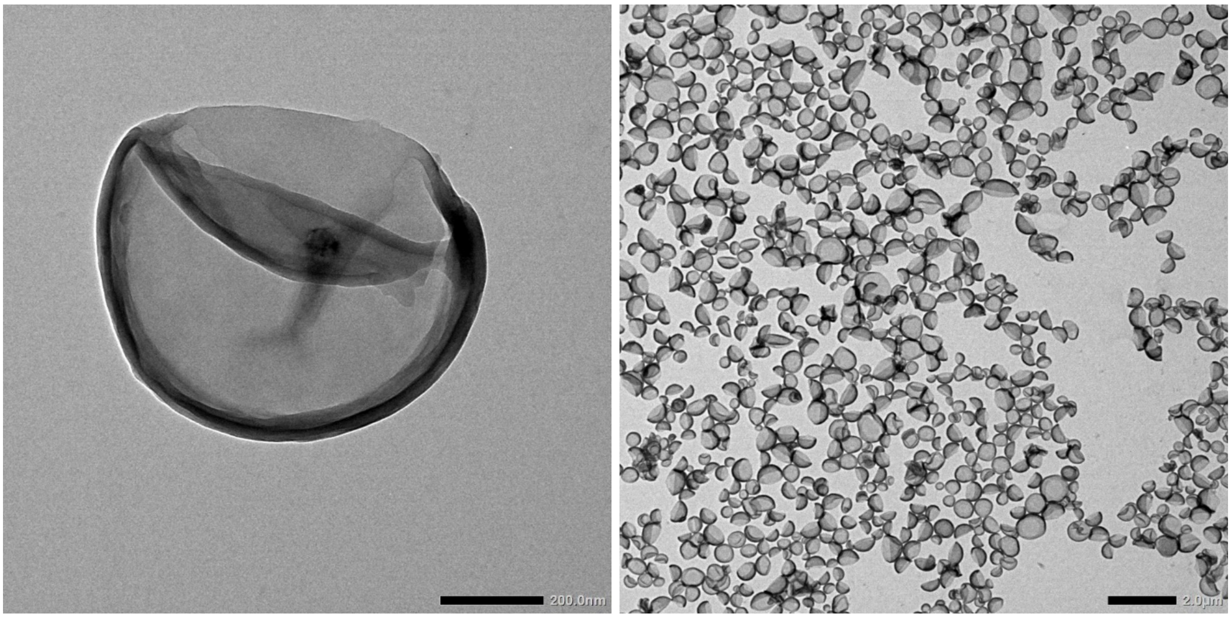

Figure S59: TEM images from water of 20% MeO-PEG$_{44}$-*b*-P(S$_{141}$-*co*-4-VBA$_{18}$)$_{159}$ cross-linked polymersomes containing 80% PEG-*b*-PS. Scale bar: 200 nm (left), 2000 nm (right)

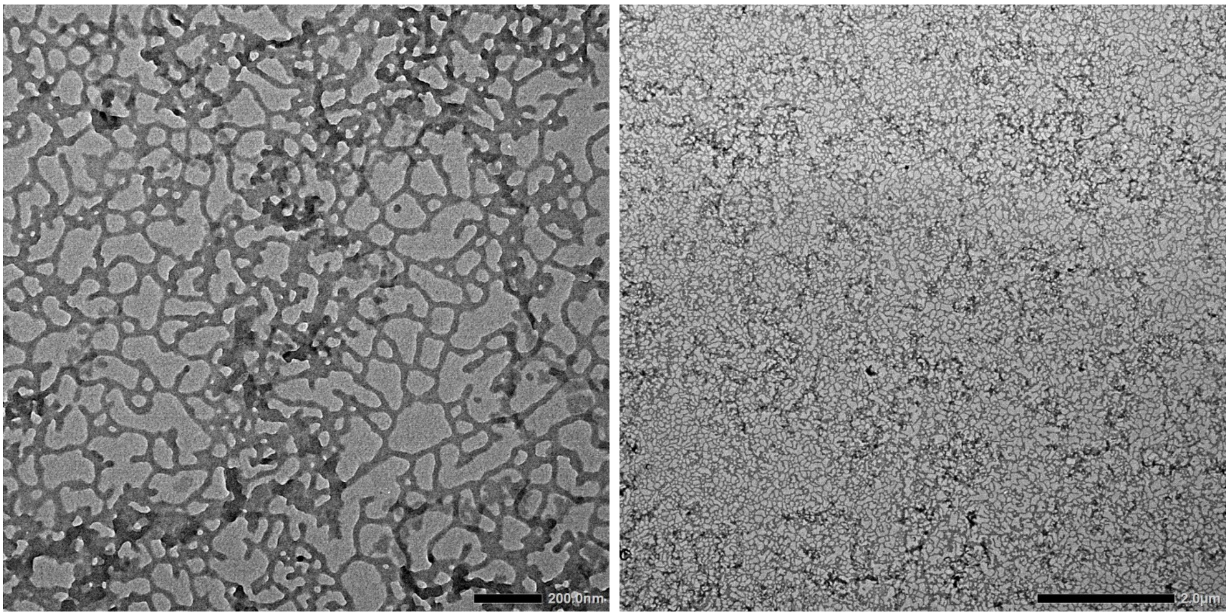

Figure S60: TEM images from THF of 20% MeO-PEG$_{44}$-*b*-P(S$_{141}$-*co*-4-VBA$_{18}$)$_{159}$ cross-linked polymersomes containing 80% PEG-*b*-PS. Scale bar: 200 nm (left), 2000 nm (right)

11. Pore Size of Cross-Linked Polymersomes with Holes

Table S1: Measured diameters (nm) of pores in the porous polymersomes. Diameters were measured from TEM images using ImageJ. n=20.

20	30	40	50	60	PEG-b-PS (%)
18.6	27.2	32.7	57.7	94.5	
19.6	20.3	31.6	43.9	49.7	
14.0	13.2	31.2	38.6	77.3	
13.1	18.7	30.2	34.7	49.4	
16.4	19.8	34.2	46.2	81.6	
18.4	20.6	35.7	42.6	53.2	
11.6	14.6	28.8	38.4	117.6	
19.1	19.7	32.0	63.8	46.3	
15.6	19.9	28.8	29.2	162.1	
16.8	16.8	27.6	29.3	86.0	
14.4	20.3	22.8	53.1	76.8	
13.7	19.0	28.3	32.7	74.2	
12.4	23.1	33.3	40.7	62.5	
14.5	20.2	30.2	25.6	48.8	
12.0	22.2	32.5	42.9	49.6	
11.6	18.8	33.3	32.8	60.2	
12.6	19.1	30.9	39.4	67.4	
11.7	21.9	32.9	92.2	111.5	
12.3	16.2	30.4	48.6	135.5	
15.3	19.3	32.6	29.3	67.0	
14.685	19.545	31	43.085	78.56	**Average**
14.2	19.75	31.4	40.05	70.8	**Median**
2.674298	3.008667	2.838087	15.25258	31.72016	**StDev**

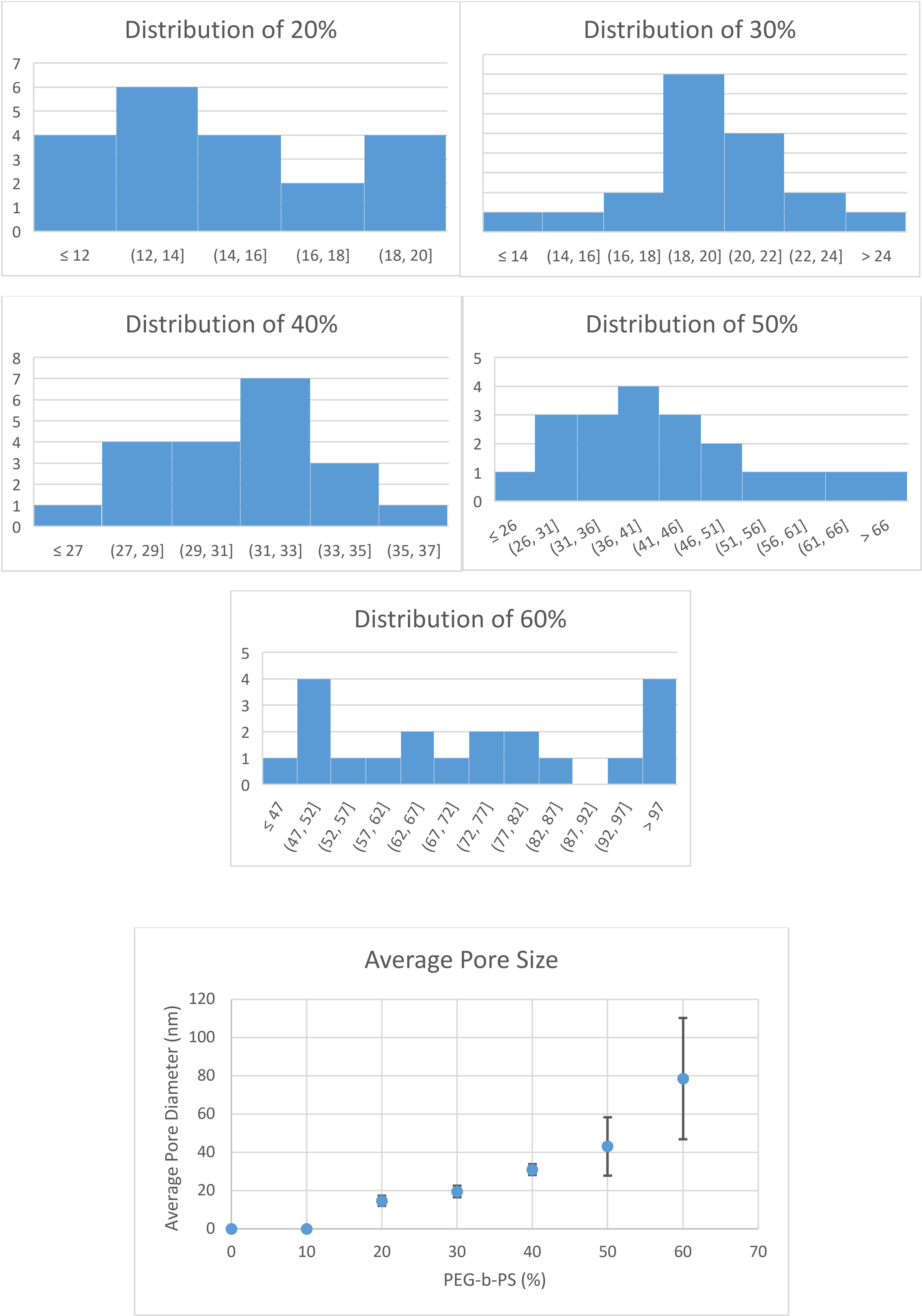

Figure S61: Plot of the average pore diameter (nm) for the different samples with holes. Hole size increases when more **6** is used. Starting from 50%, the deviation in pore size increases significantly.

12. Cryo-SEM image of Cross-Linked Polymersomes with Holes

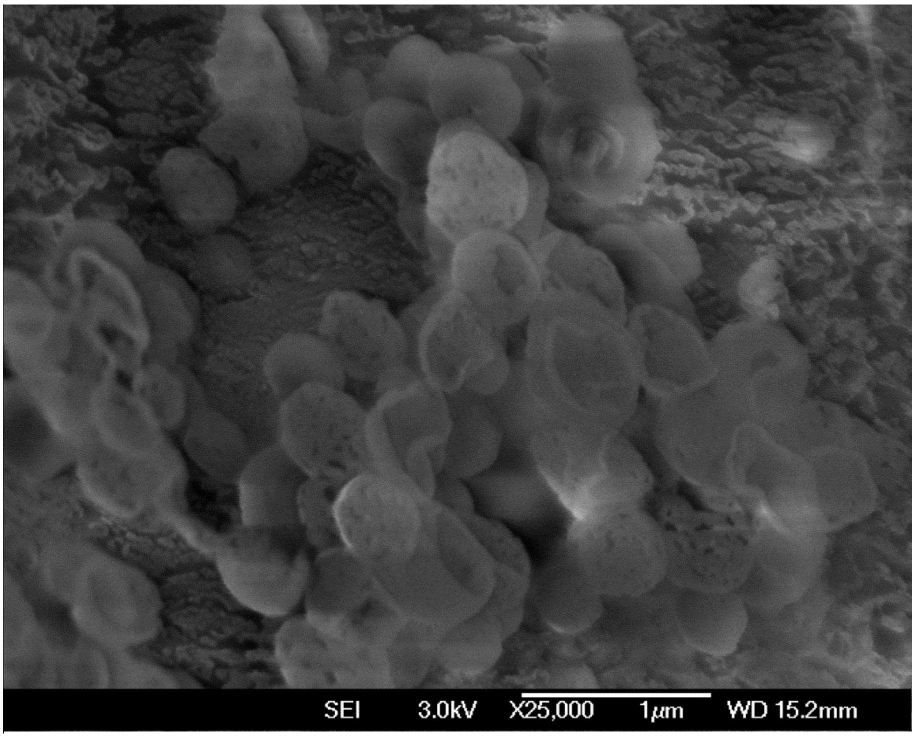

Figure S62: Cryo-SEM image of spherical 60% MeO-PEG-*b*-P(S_{141}-*co*-4-VBA_{18})$_{159}$ and 40% PEG-*b*-PS polymersomes. Full version of Figure 5C. Scale bar: 1000 nm

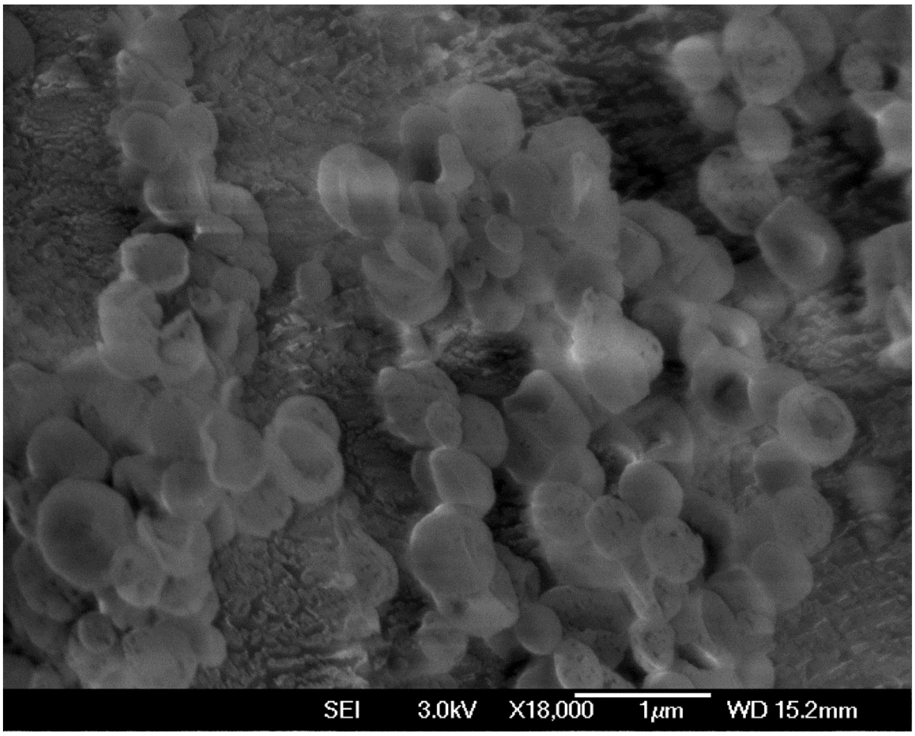

Figure S63: Cryo-SEM image of spherical 60% MeO-PEG-*b*-P(S_{141}-*co*-4-VBA_{18})$_{159}$ and 40% PEG-*b*-PS polymersomes. Scale bar: 1000 nm

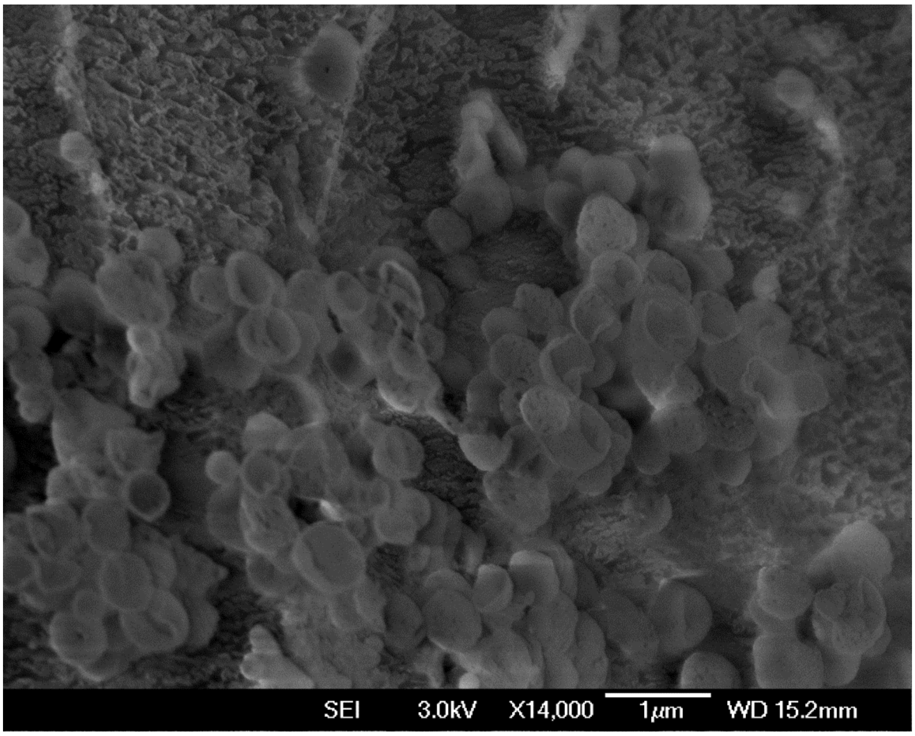

Figure S64: Cryo-SEM image of spherical 60% MeO-PEG-*b*-P(S_{141}-*co*-4-VBA_{18})$_{159}$ and 40% PEG-*b*-PS polymersomes. Scale bar: 1000 nm

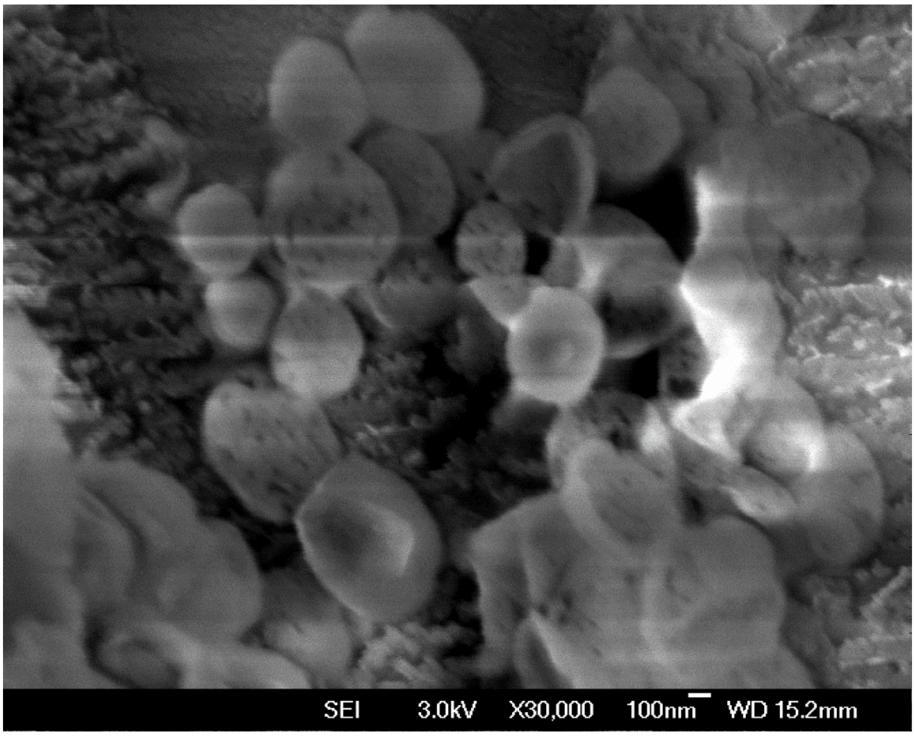

Figure S65: Cryo-SEM image of spherical 60% MeO-PEG-*b*-P(S_{141}-*co*-4-VBA_{18})$_{159}$ and 40% PEG-*b*-PS polymersomes. Scale bar: 100 nm

13. Fluorescence data of Pt NP + Propargyl-carbamate Masked Coumarin Reaction

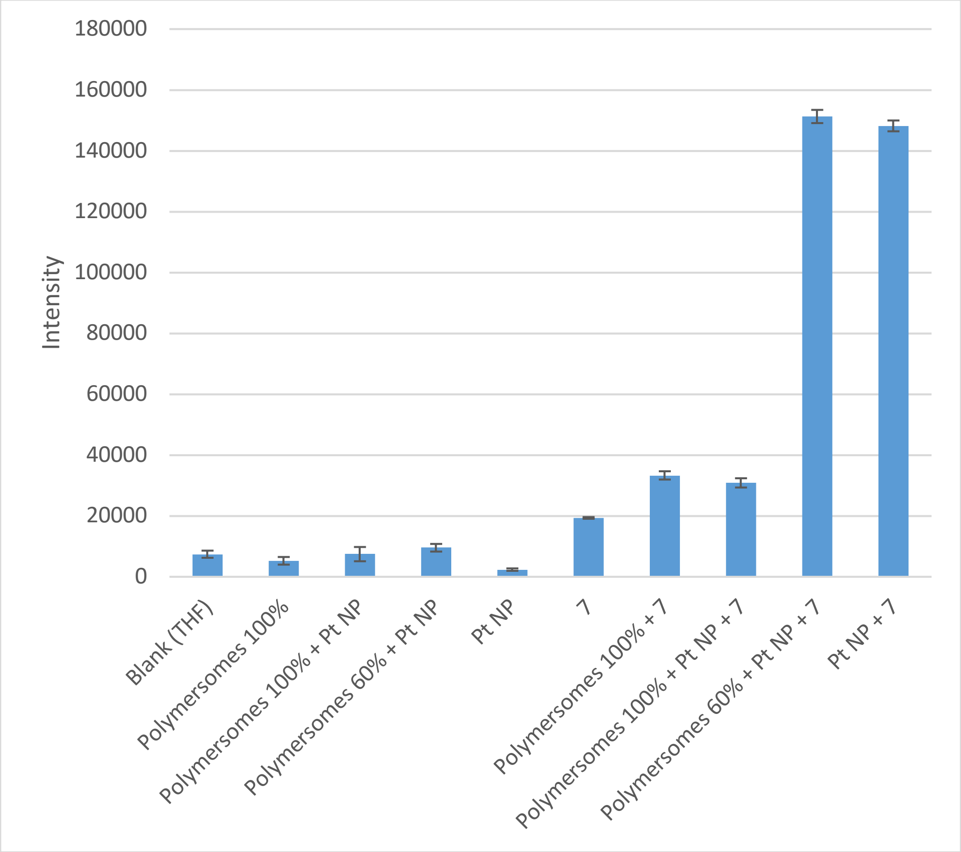

Figure S66: Plot of fluorescence measurements of various combinations of cross-linked MeO-PEG_{44}-*b*-P(S_{141}-*co*-4-VBA_{18})$_{159}$ polymersomes (100% and 60%), platinum nanoparticles and Prop-2-yn-1-yl (4-methyl-2-oxo-2H-chromen-7-yl)carbamate **7** after 15 minutes 37 °C, diluted 50x from regular concentration. Error bars are based on the standard deviation of nine measurements. All components without addition of **7** are taken as a blank. Excitation was performed using a filter for 365 nm (20 nm bandwidth) and emission was measured with a filter for 440 nm (20 nm bandwidth). Relative fluorescence is given, as a relative comparison was all that was desired. **7** gives a slightly higher fluorescence compared to the blank, most likely due to residual amounts of fluorescence despite the propargyl group. Addition of cross-linked polymersomes with and without encapsulated Pt NPs give a similar amount of fluorescence, indicating no reaction takes place. A fivefold increase is measured for the addition Pt NPs as well as for the addition of 60% cross-linked polymersomes containing Pt NPs, indicating the availability of the Pt NP catalyst for the substrate in the 60% polymersomes.

14. NMR and GPC spectra of polymers

1. Compound **1** 45

2. Compound **2** 47

3. Compound **3** 49

4. Compound **4** 51

5. Compound **5** 53

6. Compound **6** 55

7. Compound **7** 57

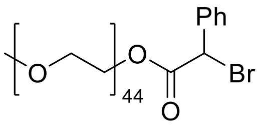

1

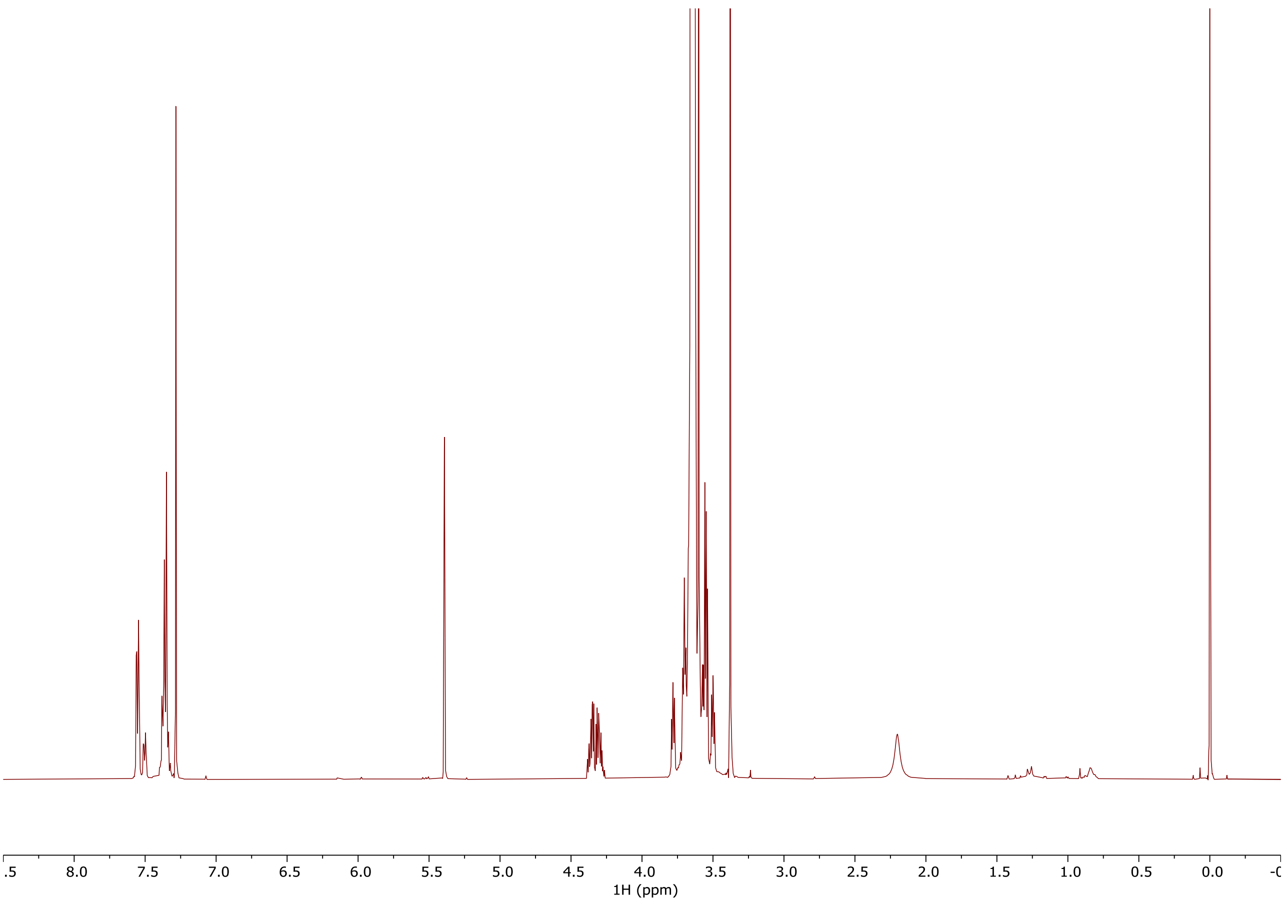

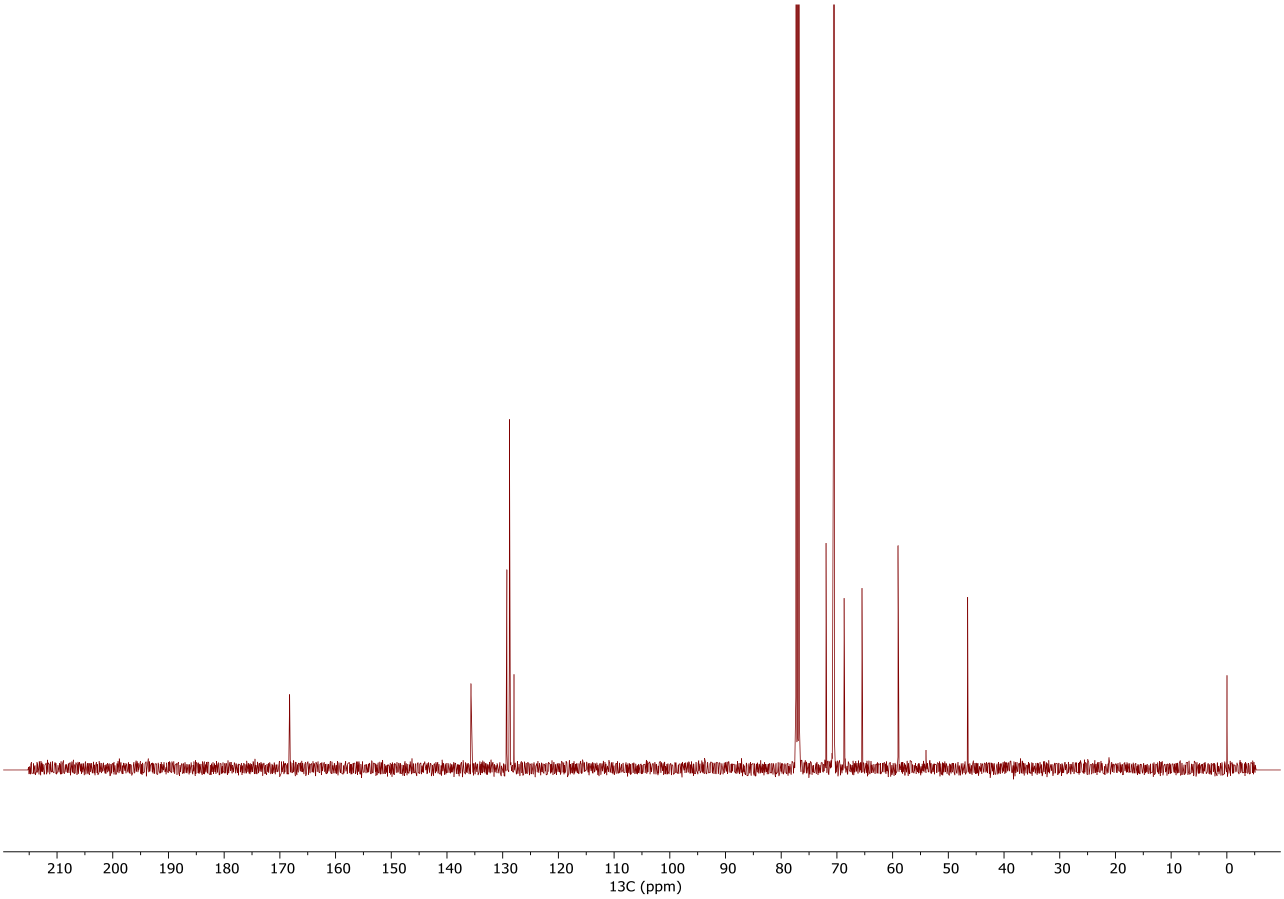
210 200 190 180 170 160 150 140 130 120 110 100 90 80 70 60 50 40 30 20 10 0
13C (ppm)

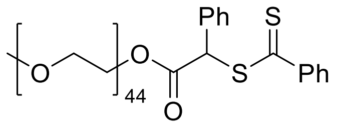

2

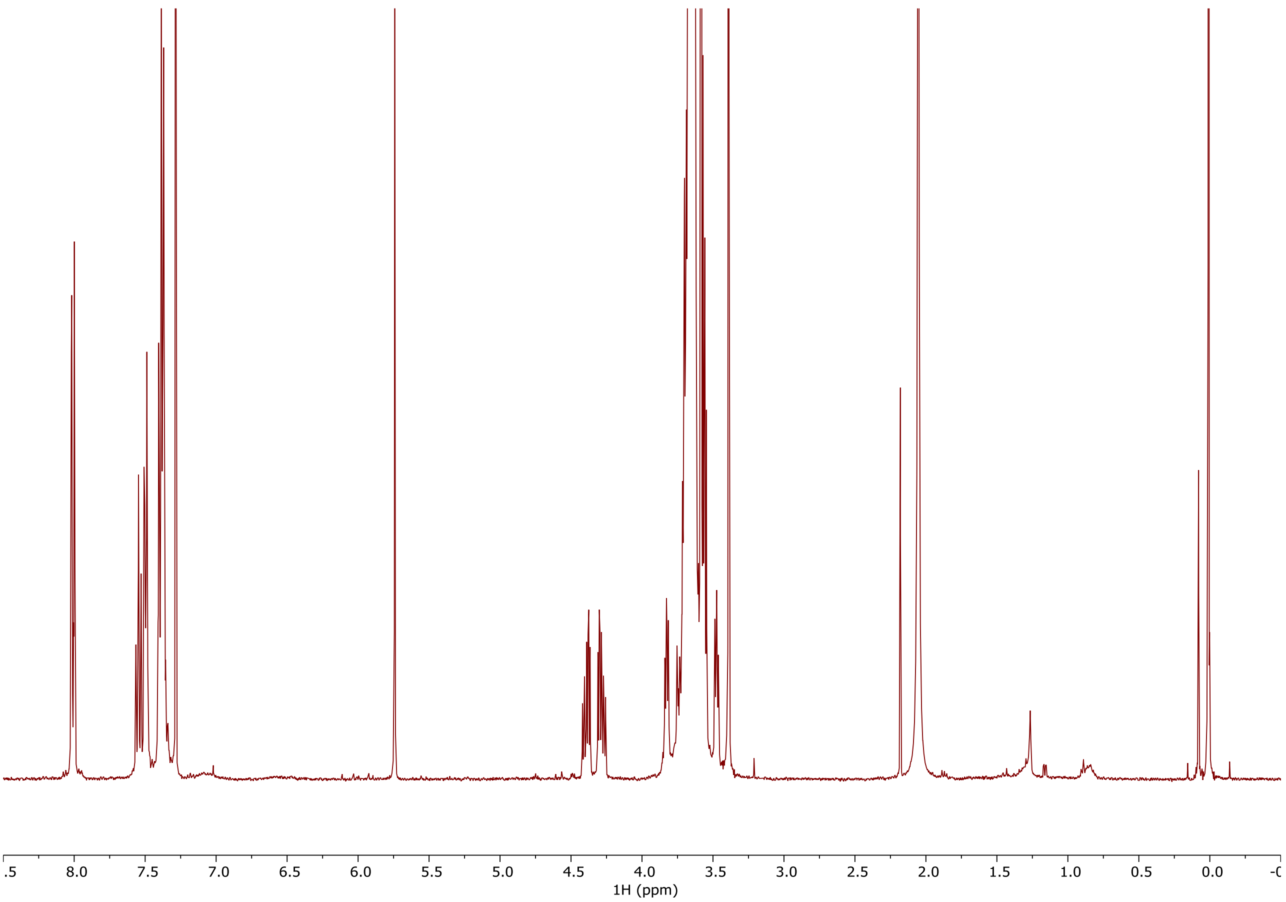

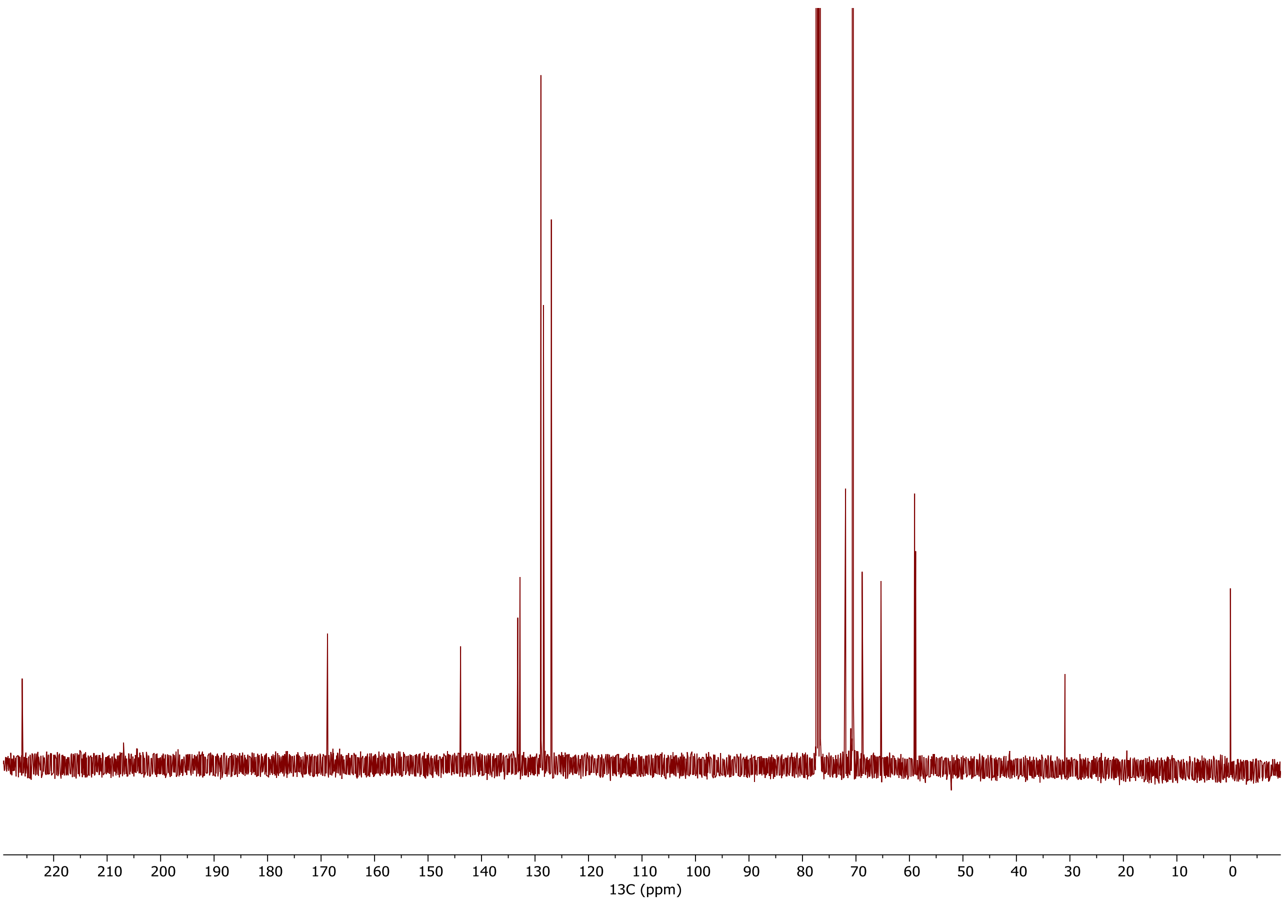
220 210 200 190 180 170 160 150 140 130 120 110 100 90 80 70 60 50 40 30 20 10 0
13C (ppm)

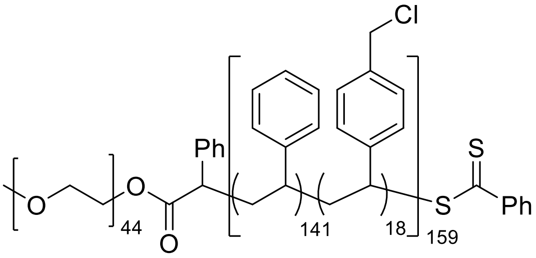
Cl
Ph
S
O
44
O
O
141
18
159
S
Ph

3

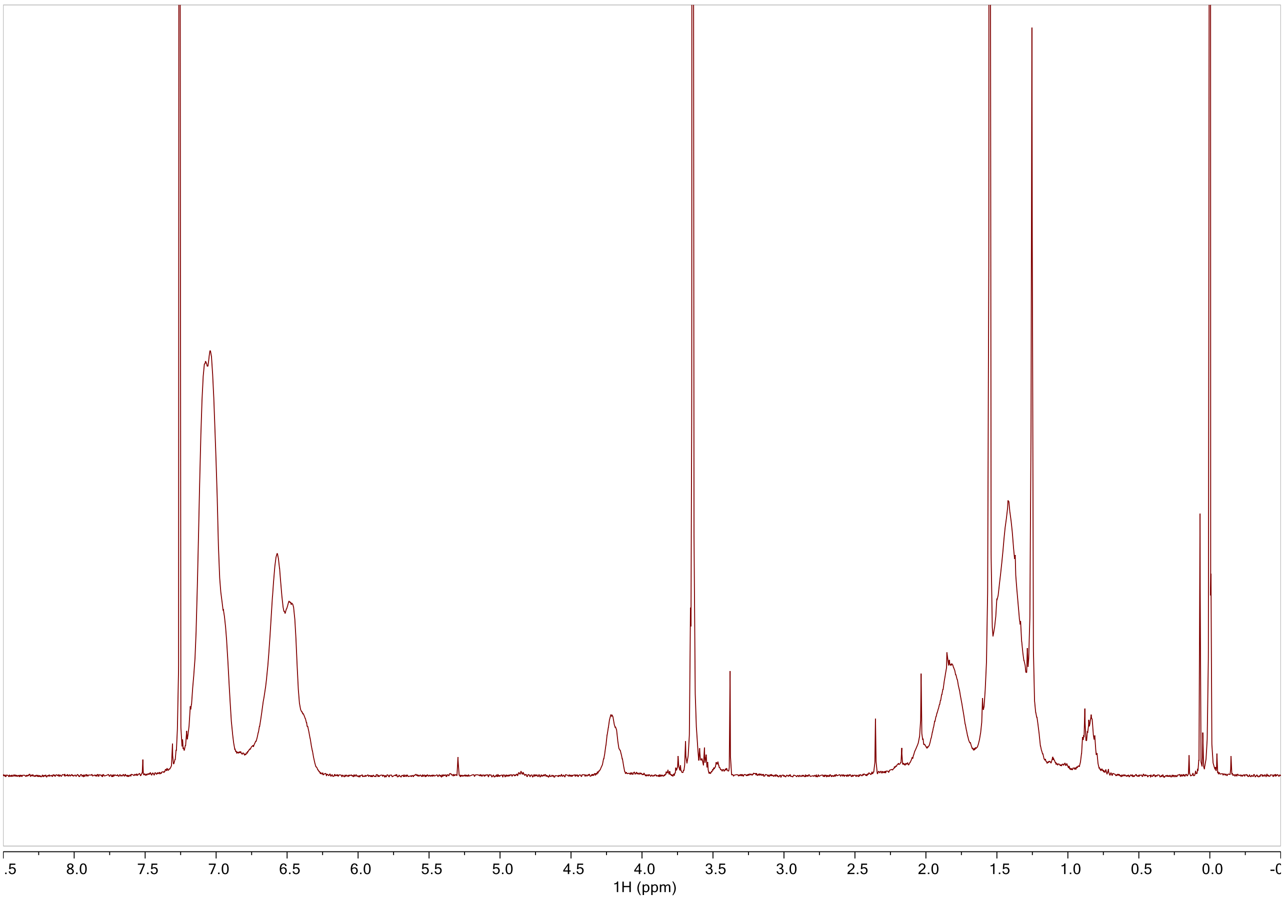
.5
8.0
7.5
7.0
6.5
6.0
5.5
5.0
4.5
4.0
3.5
3.0
2.5
2.0
1.5
1.0
0.5
0.0
1H (ppm)

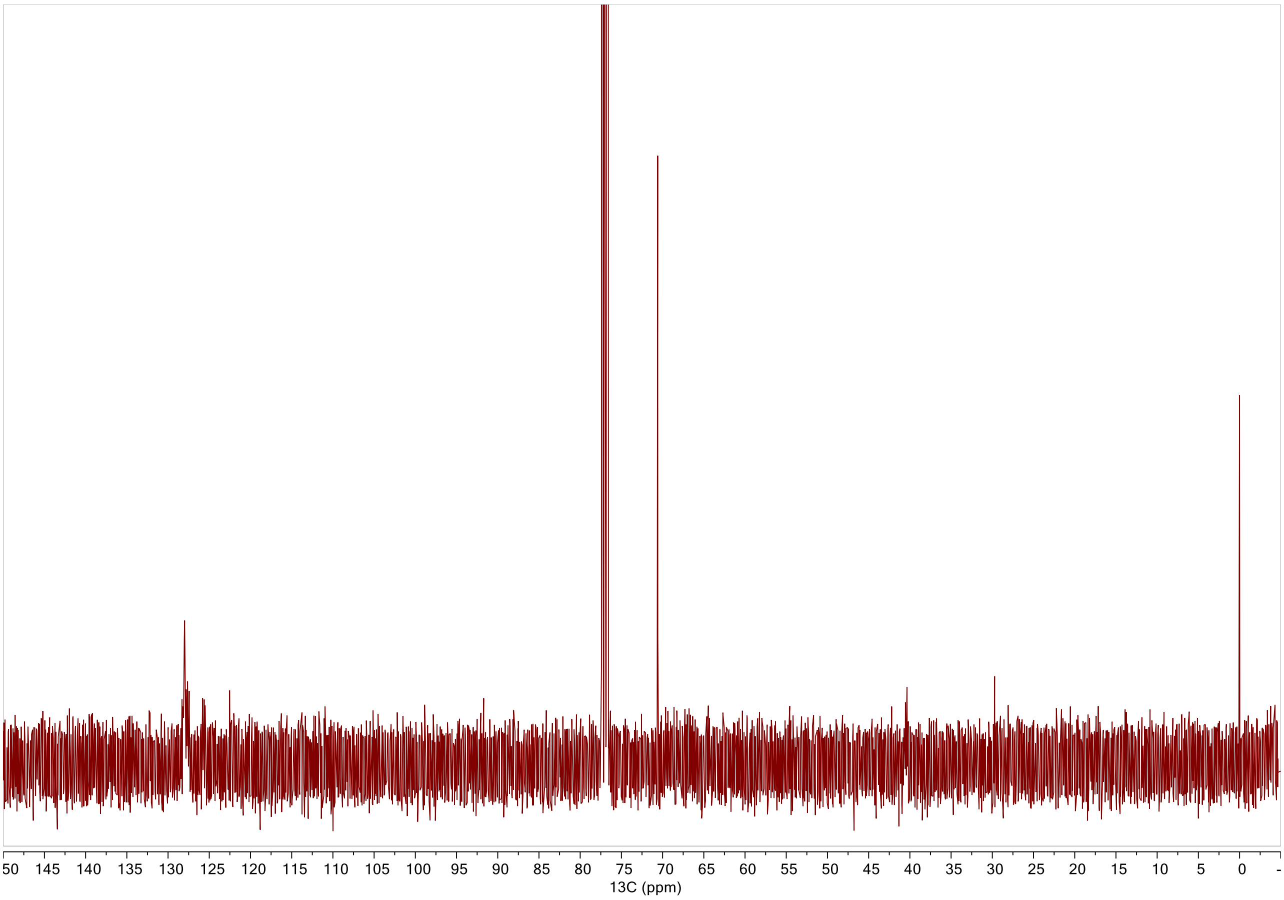
50 145 140 135 130 125 120 115 110 105 100 95 90 85 80 75 70 65 60 55 50 45 40 35 30 25 20 15 10 5 0
13C (ppm)

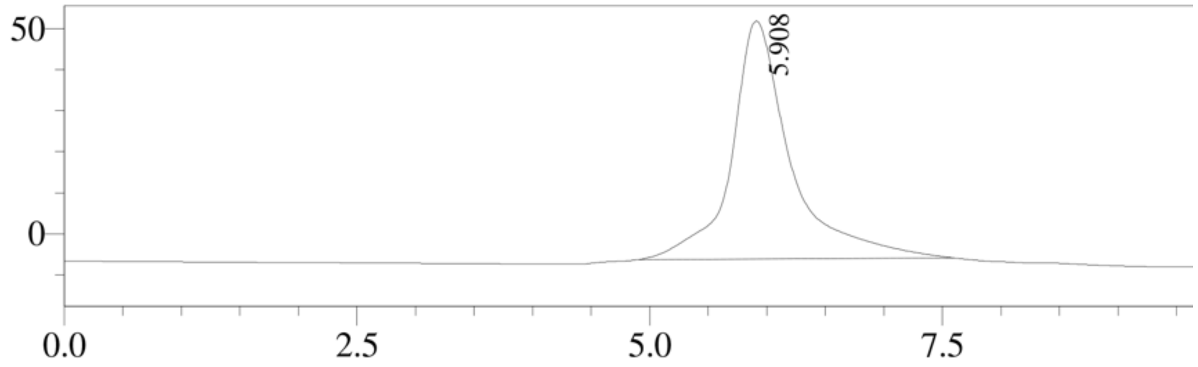
50
0
5.908
0.0 2.5 5.0 7.5

Mw/Mn 1.13383

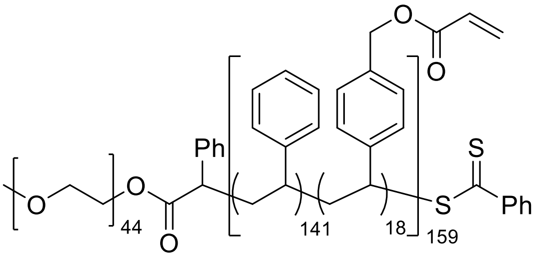

O
O
O
Ph
O
O
S
S
Ph
44
141
18
159

4

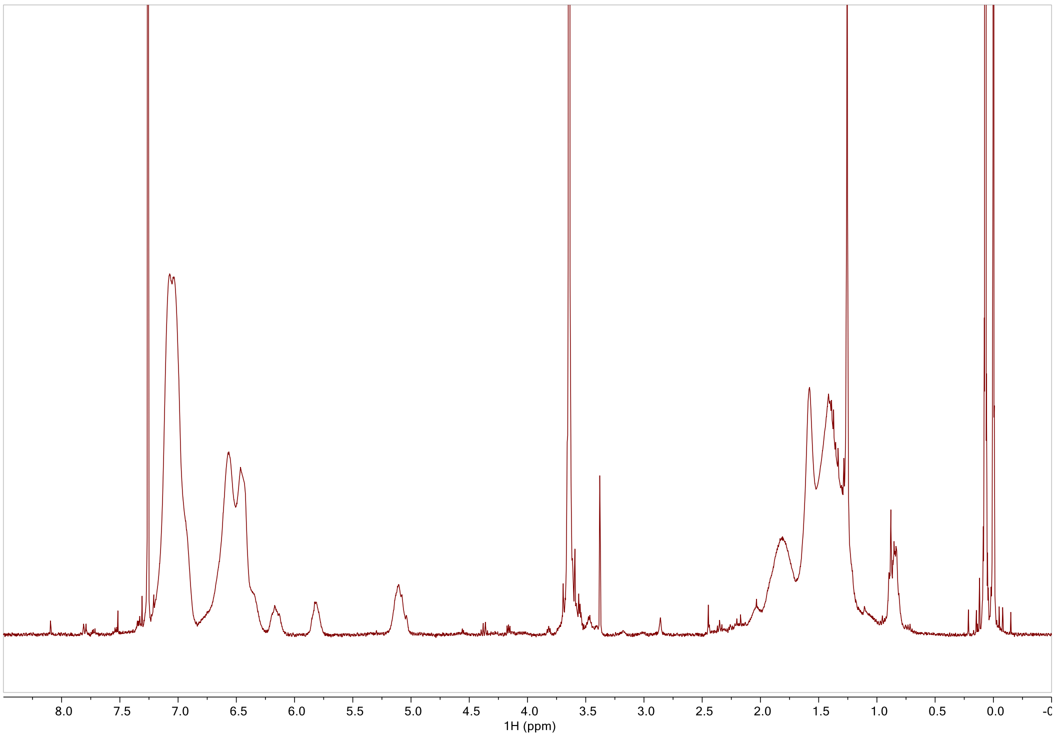

8.0
7.5
7.0
6.5
6.0
5.5
5.0
4.5
4.0
3.5
3.0
2.5
2.0
1.5
1.0
0.5
0.0
1H (ppm)

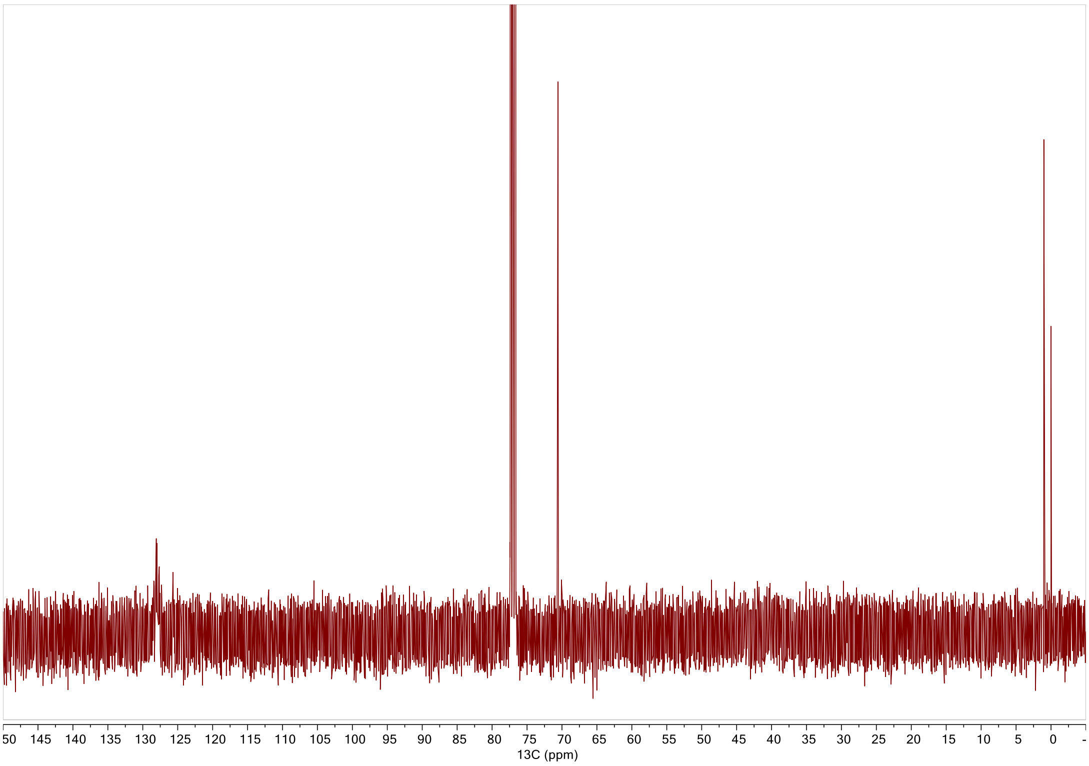
50
145
140
135
130
125
120
115
110
105
100
95
90
85
80
75
70
65
60
55
50
45
40
35
30
25
20
15
10
5
0
13C (ppm)

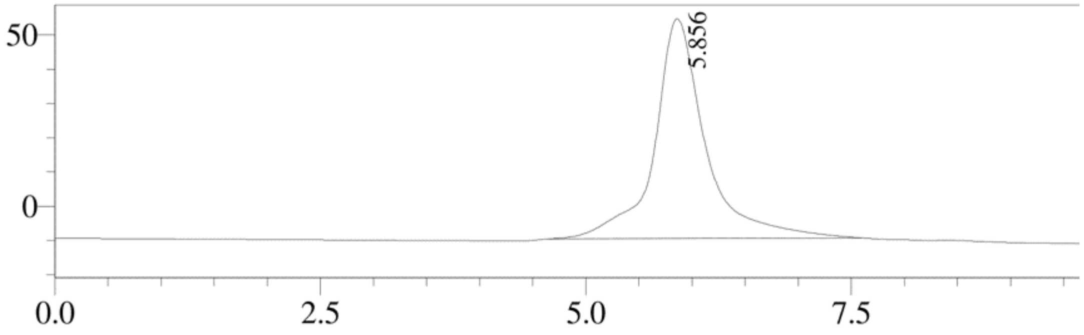
50
0
5.856
0.0
2.5
5.0
7.5

Mw/Mn 1.12637

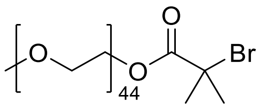

5

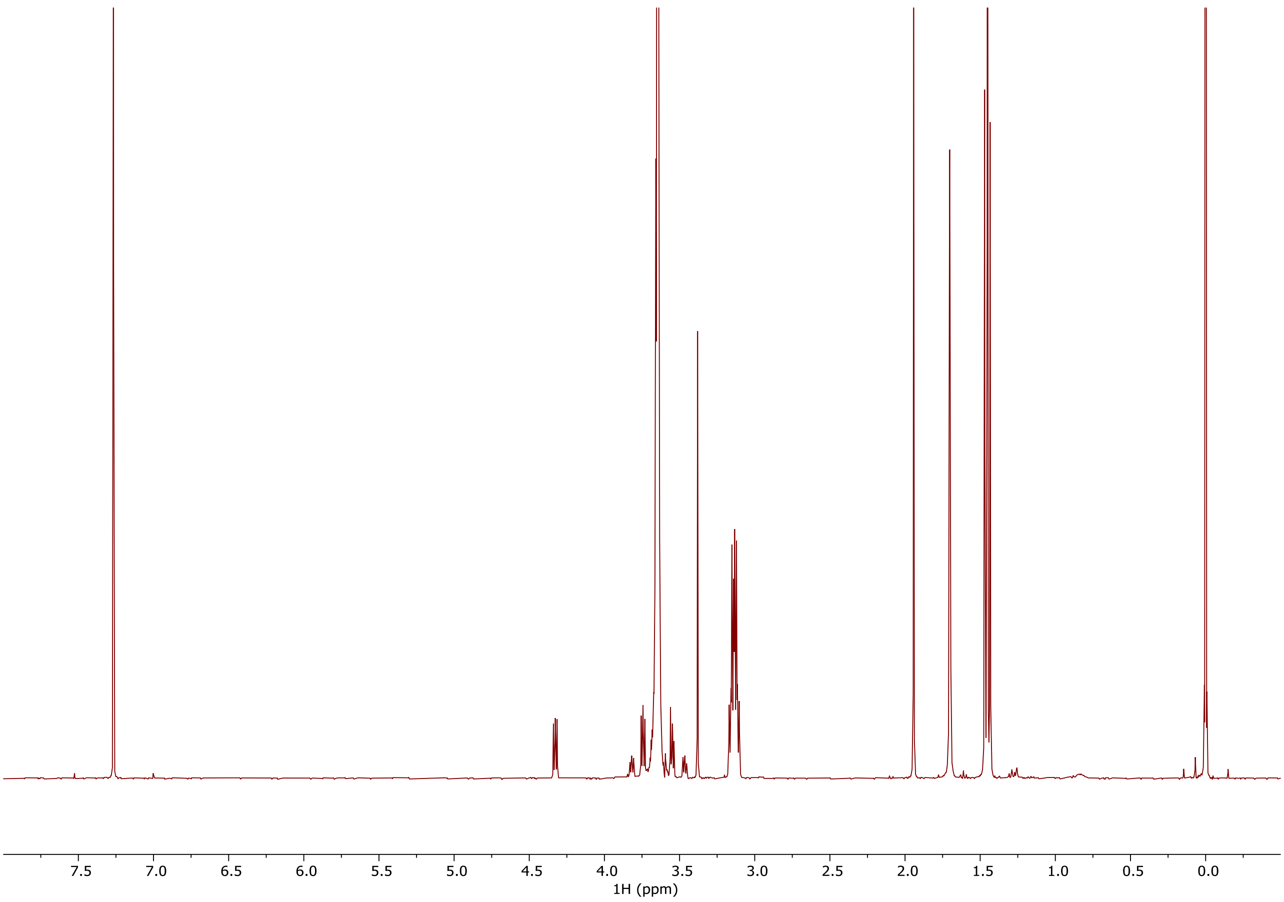

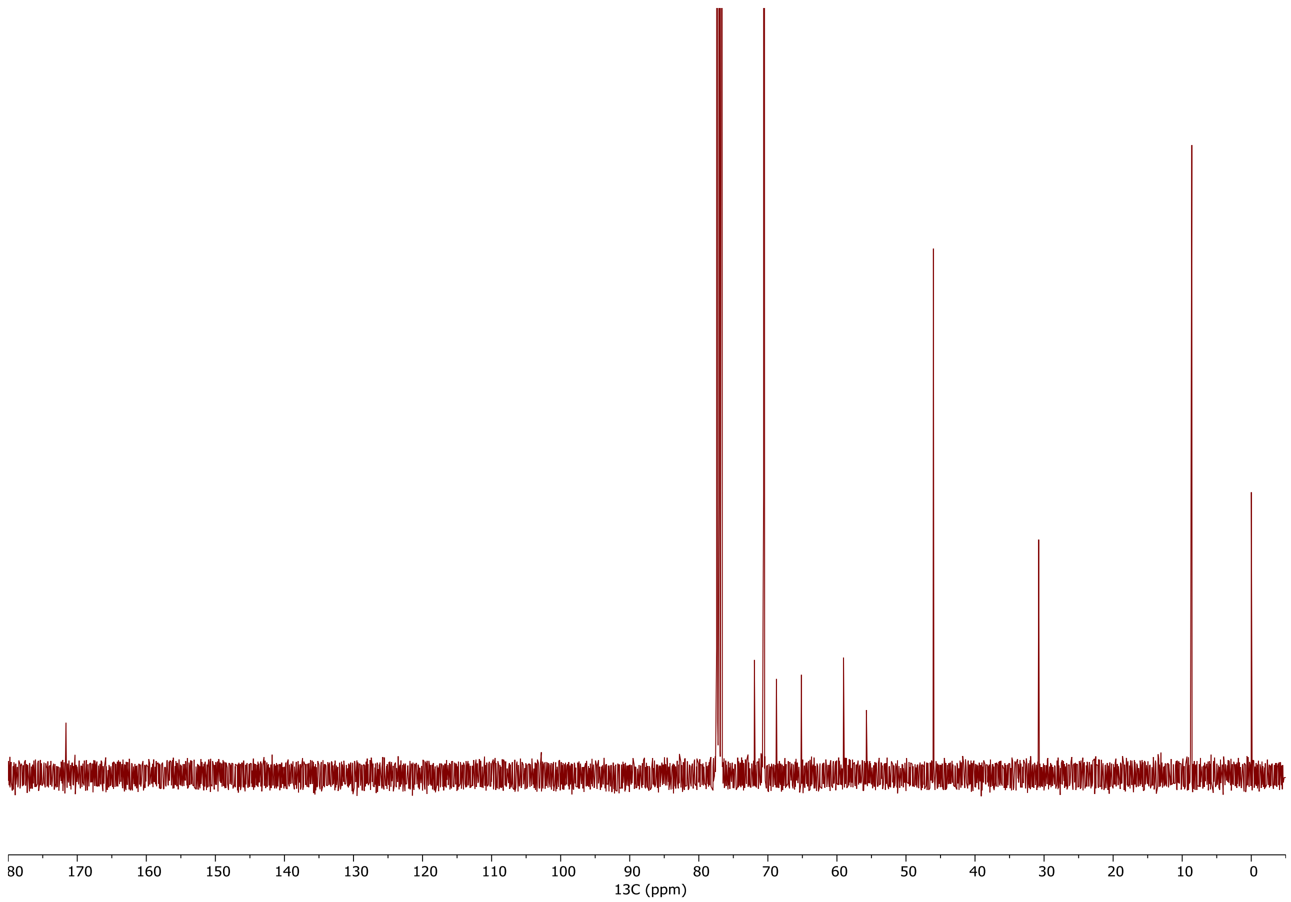
80
170
160
150
140
130
120
110
100
90
80
70
60
50
40
30
20
10
0
13C (ppm)

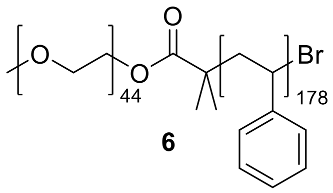

6

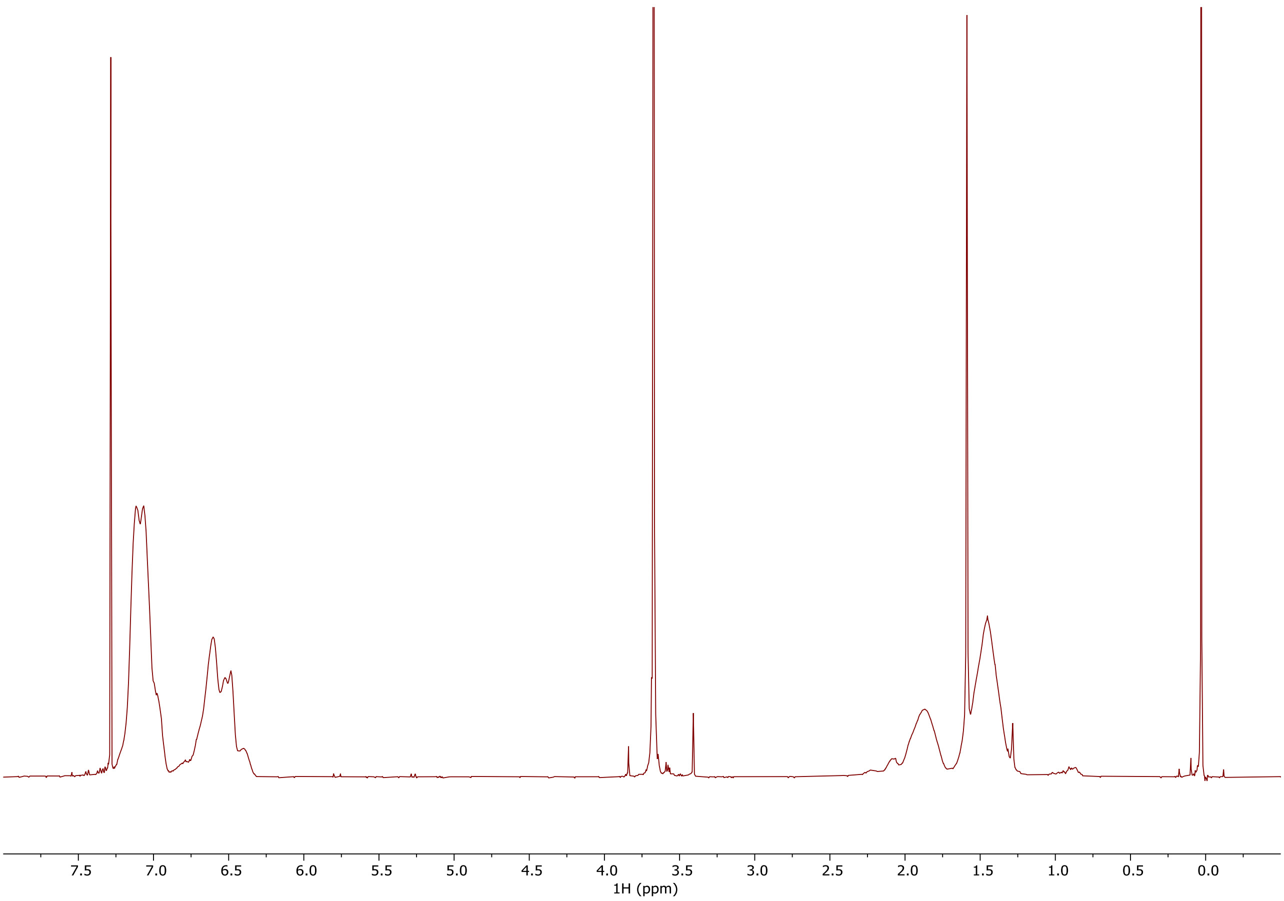

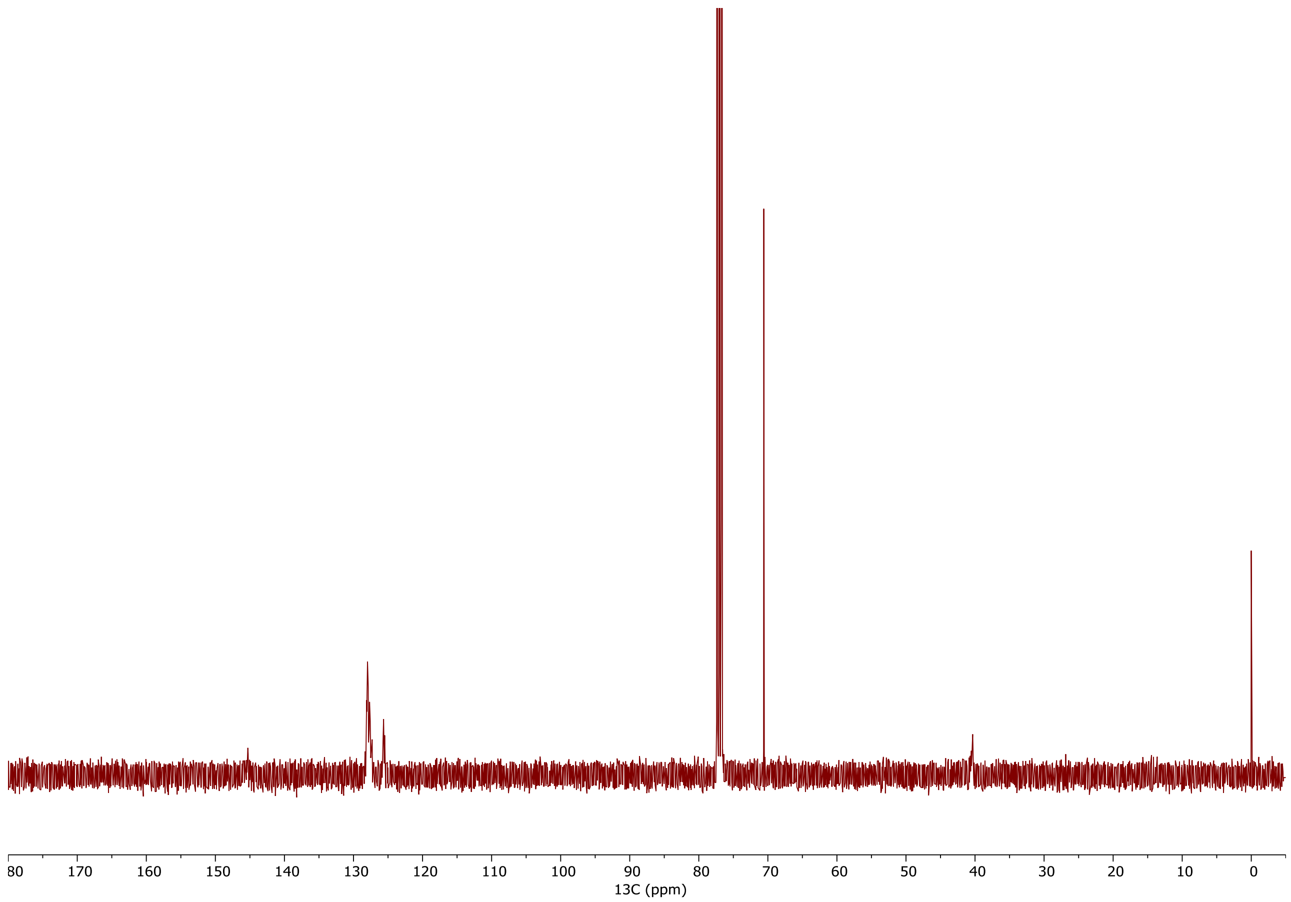
80
170
160
150
140
130
120
110
100
90
80
70
60
50
40
30
20
10
0
13C (ppm)

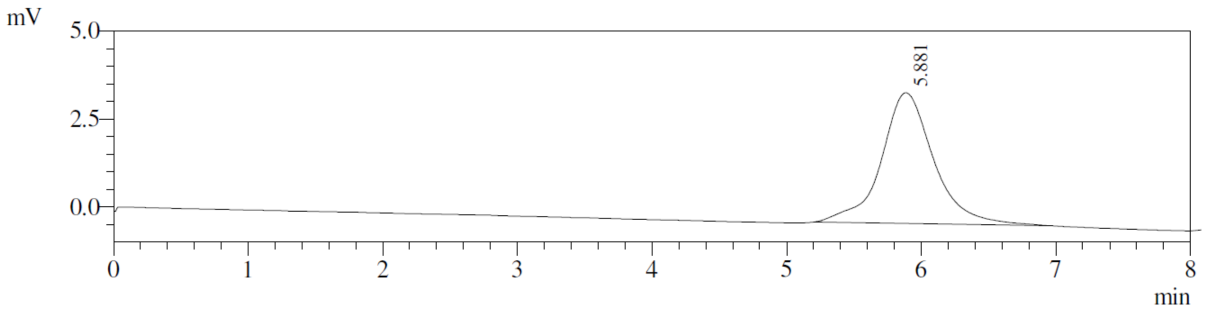
mV
5.0
2.5
0.0
5.881
0
1
2
3
4
5
6
7
8
min

Mw/Mn 1.05185

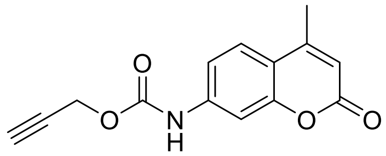

7

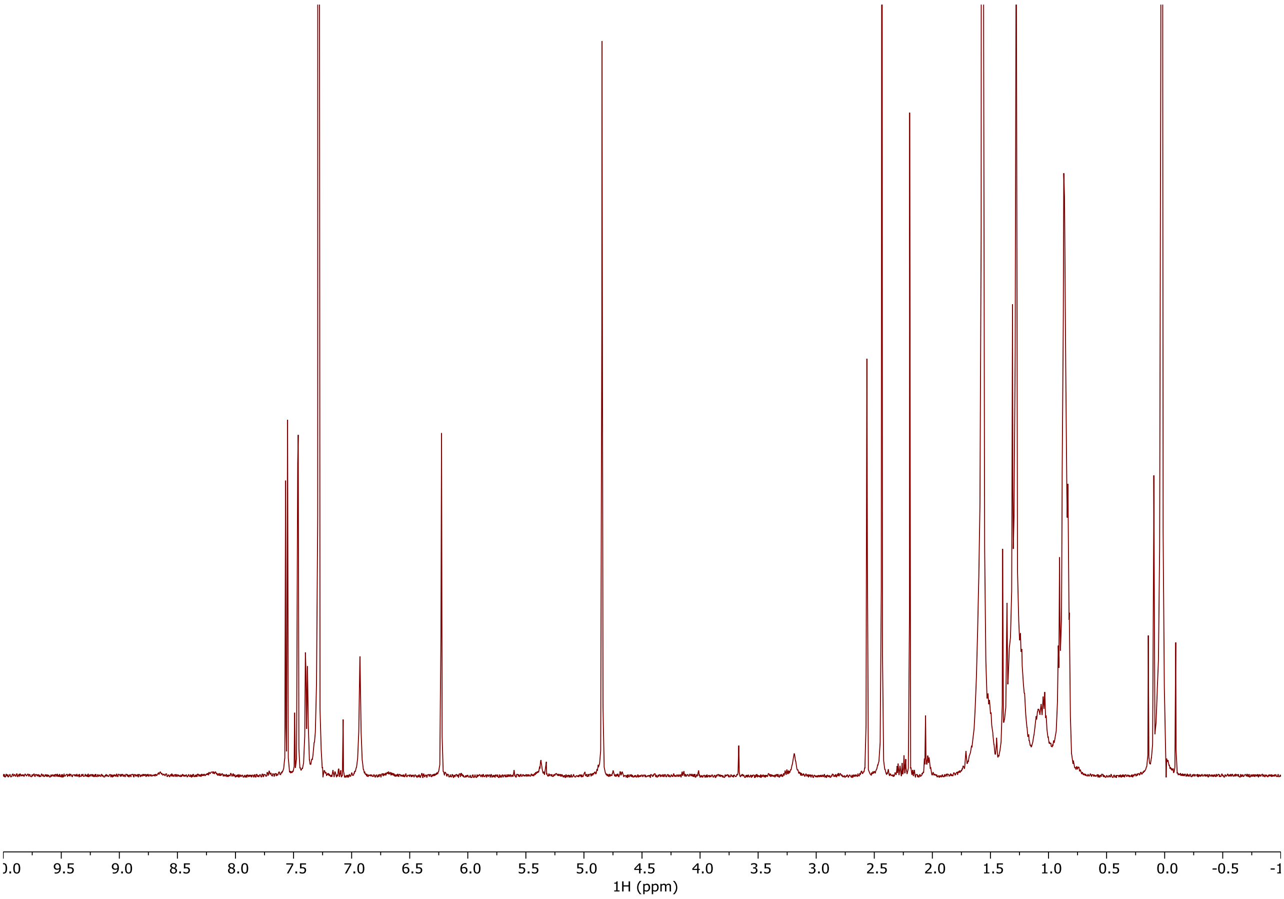

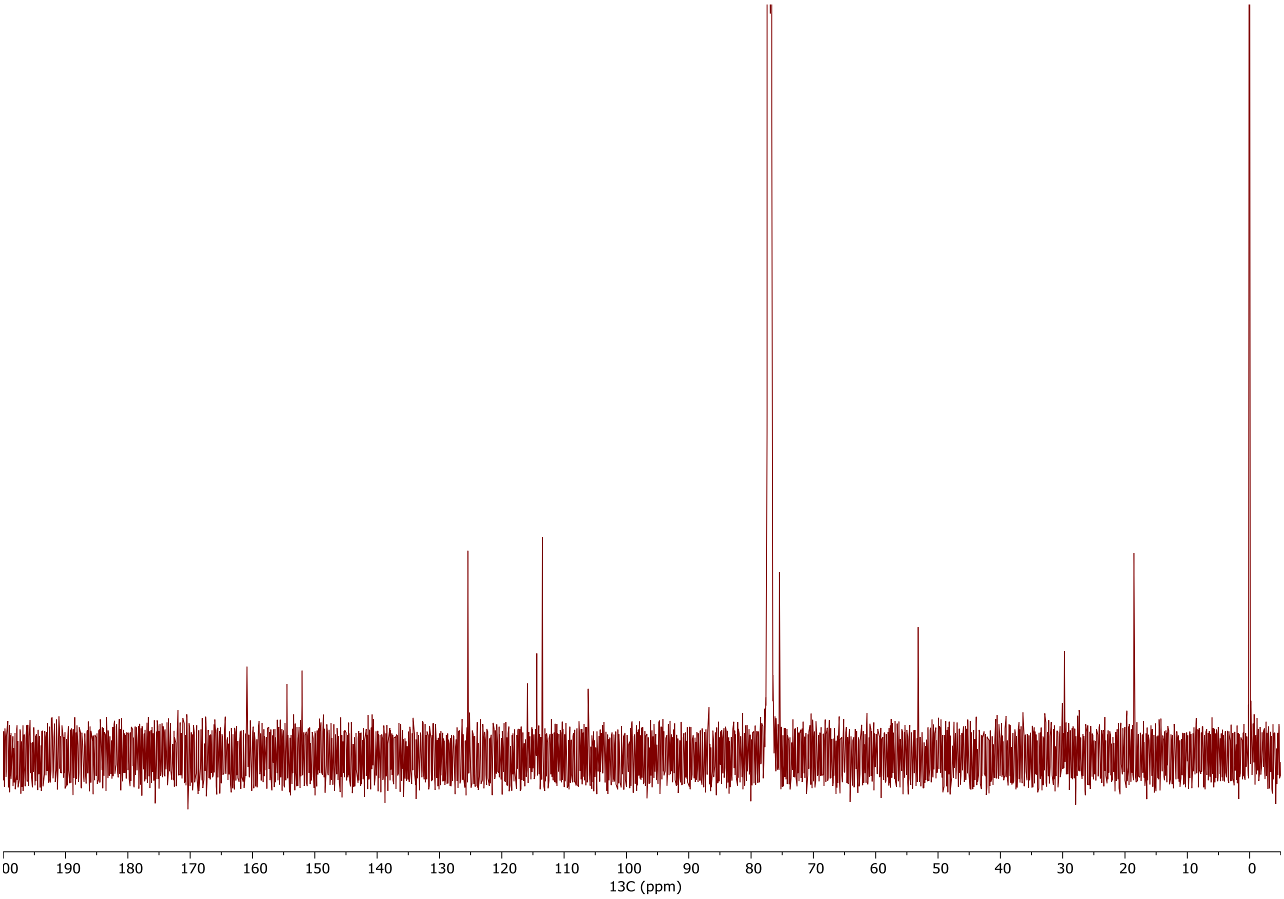

15. References

[1] Nishikubo, T.; Iizawa, T.; Kobayashi, K.; Okawara, M., Facile synthesis of p-chloromethylated styrene by elimination reaction of p-(2-bromoethyl)benzylchloride using potassium hydroxide as a base under phase transfer catalysis. *Tetrahedron Lett.* **1981,** *22* (39), 3873–3874.

[2] Tanimoto, S.; Miyake, T.; Okano, M., A Convenient Preparation of p-Chloromethyl-Styrene and its Reactions. *Synth. Commun.* **1974,** *4* (4), 193–197.

[3] Schindelin, J.; Arganda-Carreras, I.; Frise, E.; Kaynig, V.; Longair, M.; Pietzsch, T.; Preibisch, S.; Rueden, C.; Saalfeld, S.; Schmid, B.; Tinevez, J.-Y.; White, D. J.; Hartenstein, V.; Eliceiri, K.; Tomancak, P.; Cardona, A., Fiji: an open-source platform for biological-image analysis. *Nat. Methods* **2012,** *9*, 676.

[4] Rijpkema, S.J.; Langens, S.G.H.A.; van der Kolk, M.K.; Gavriel, K.; Toebes, B.J.; Wilson, D.A., Modular Approach to the Functionalization of Polymersomes, *Biomacromolecules* **2020**, *21* (5), 1853–1864.
